# Genotype imputation and error estimation in connected multiparental populations

**DOI:** 10.1093/genetics/iyag217

**Published:** 2026-08-17

**Authors:** Chaozhi Zheng, Eligio Bossolini, Nataša Formanová, Antje Rohde, Martin P Boer, Fred A van Eeuwijk

**Affiliations:** Mathematical & Statistical Methods—Biometris, Wageningen University & Research, Wageningen 6708PB, Gelderland, the Netherlands; BASF Belgium Coordination Center CommV, Technologiepark-Zwijnaarde 101, Ghent 9052, East Flanders, Belgium; BASF Belgium Coordination Center CommV, Technologiepark-Zwijnaarde 101, Ghent 9052, East Flanders, Belgium; BASF Belgium Coordination Center CommV, Technologiepark-Zwijnaarde 101, Ghent 9052, East Flanders, Belgium; Mathematical & Statistical Methods—Biometris, Wageningen University & Research, Wageningen 6708PB, Gelderland, the Netherlands; Mathematical & Statistical Methods—Biometris, Wageningen University & Research, Wageningen 6708PB, Gelderland, the Netherlands

**Keywords:** connected populations, MPP, multiparental populations, multiparent advanced generation intercross (MAGIC), next-generation sequencing, allelic bias

## Abstract

Multiparental populations have been produced for quantitative trait loci mapping in many crops, where next-generation sequencing has become a cost-effective tool for genotyping. Previously, we have developed a hidden Markov framework denoted by MagicImpute_mma for genotype imputation in a multiparental population, which was implemented in Mathematica. However, its computational time increases quickly with the number of founders. In this work, we extend MagicImpute_mma into MagicImpute for increasing computational efficiency and robustness to various types of errors. Particularly, it has the following novel features: (i) allowing for multiple multiparental populations that may be connected by sharing founders, (ii) allowing for many missing founders that are not available for sequencing, (iii) accounting for allelic bias and overdispersion in next-generation sequencing data, (iv) inferring marker-specific error rates and filtering for markers with low error rates, and (v) being implemented in the high-performance Julia language. Besides extensive simulation studies, we evaluate MagicImpute by 3 real datasets: the rice F2 population with sequence depth being low, the apple cross-pollinated population with parents being outbred, and the sorghum multiparent advanced generation intercross population with 10 male sterile lines (out of 29 founders) missing. The results show that MagicImpute is well capable of imputing many missing founders if there are sufficient offspring (eg 20 per founder) and that MagicImpute is accurate for genotype imputation in connected bi- or multiparental populations with various types of sequence errors, and it opens up new opportunities for quantitative trait loci mapping after imputing many missing founders.

## Introduction

Multiparental populations are experimental crosses usually produced by first outcrossing many inbred or outbred parents, then intercrossing systematically or randomly for some generations, and finally and optionally inbreeding by several generations of selfing or sibling mating. In comparison with traditional biparental populations, multiparental populations have high genetic diversity, and thus many such populations have been recently produced for quantitative trait loci (QTL) mapping in crops such as maize (Dell’Acqua et al. 2015) and sorghum (Ongom and Ejeta 2018). For a given species, particularly in commercial-scale plant breeding, many bi- or multiparental populations are sometimes available, and they are often connected by sharing parents.

Missing genotypic data in connected multiparental populations pose challenges for haplotype reconstruction that is usually required for downstream QTL mapping or genomic prediction. Although there are packages allowing for some missing data, including R/HAPPY (Mott et al. 2000) and MagicReconstruct_mma (Zheng et al. 2015), they do not work well when there is a large fraction of missing data, particularly when there are some connecting founders with all genotypes missing. The focus of this work is methodology development for genotype imputation in connected bi- or multiparental populations.

Genotype imputation methods have been originally developed for applications in human genetics (eg the review by Sun and Li 2025), which can be generally categorized into population-based imputation using linkage disequilibrium information and pedigree-based imputation using linkage information. However, these methods are not optimized for experimental crosses in plant breeding. For example, the pedigree-based methods such as Merlin (Abecasis et al. 2002) and GIGI (Cheung et al. 2013) are not efficient for breeding pedigrees with selfing and ungenotyped members in intermediate generations, whereas the population-based methods such as Beagle 5.5 (Browning et al. 2018) and IMPUTE2 (Howie et al. 2009) are tailored to work with a reference haplotype panel and they cannot utilize pedigree information.

There are several existing methods for imputation in biparental populations, including FSFHap (Swarts et al. 2014), LB-Impute (Fragoso et al. 2016), and AlphaFamImpute (Whalen et al. 2020). In comparison, methods for multiparental populations are relatively few, including mpimpute (Huang et al. 2014), R/qtl2 (Broman et al. 2019), and MagicImpute_mma (Zheng et al. 2018a). However, mpimpute and R/qtl2 work only for specific mating designs and do not allow for missing data in parents. Although MagicImpute_mma has no such limitations, it is not computationally efficient in the case of many parents. Specifically, MagicImpute_mma performs founder imputation by analyzing all possible founder haplotypes along chromosomes, and thus the computational time increases quickly with the number of missing founder genotypes at each marker.

The primary aim of this work is to extend MagicImpute_mma (Zheng et al. 2018a) for increasing computational efficiency in the case of many parents. Besides implementing MagicImpute in the high-performance Julia language (Bezanson et al. 2017) instead of Mathematica (Wolfram Research 2016) for MagicImpute_mma (Zheng et al. 2018a), we extend the founder imputation algorithm for connected multiparental populations, particularly allowing for some or all founders’ genotypes being completely missing. Specifically, we partition founders into blocks and impute founder blocks alternately, and thus computational time depends mainly on the founder block sizes rather than the total number of founders.

The second aim of this work is to increase imputation robustness and heterozygous genotype calling accuracy for low coverage genotyping by sequencing (GBS) data. Incorrect heterozygous calling can be due to low read depth and sequence errors. Allelic bias is a typical source of error, possibly because the polymerase chain reaction (PCR) amplification efficiency depends on many factors such as GC content and the length of restriction fragments in sequence technologies (Schirmer et al. 2015; Salk et al. 2018; Qin et al. 2023). Genotype-Corrector (Miao et al. 2018) and GBScleanR (Furuta et al. 2023) have developed a binomial model for GBS data with the probability parameter accounting for allelic bias. We further develop a beta-binomial model to account for both allelic bias and overdispersion.

This paper is organized as follows. We first describe the MagicImpute algorithm for connected multiparental populations. Then we evaluate MagicImpute through extensive simulation studies, including benchmarking MagicImpute against previous software such as Beagle 5.5 (Browning et al. 2018), MagicImpute_mma (Zheng et al. 2018a), and GBScleanR (Furuta et al. 2023), and testing the imputation of many ungenotyped founders and the estimation of various error rates. Furthermore, we evaluate MagicImpute on the real sorghum multiparent advanced generation intercross (MAGIC) with 10 out of 29 founders being ungenotyped (Ongom and Ejeta 2018) and 2 heterozygous populations: the rice F2 (Furuta et al. 2017) and the apple cross-pollinated (CP) population (Gardner et al. 2014) with low coverage GBS data. In addition, we compare MagicImpute with GBScleanR (Furuta et al. 2023) using the rice F2. Finally, we discuss the MagicImpute algorithm from the aspects of founder imputation and error estimation.

## Methods

### Overview of MagicImpute

The target population of MagicImpute is a population consisting of one or more subpopulations. Each subpopulation is derived from one or multiple founders via a few generations of mating crosses, and offspring in each subpopulation are exchangeable and thus they have the same prior genomic contributions from founders. A subdivided population is also termed a connected population if its subpopulations are connected by sharing founders. Figure 1a-c shows 3 example target populations. MagicImpute requires 2 input files: a variant call format (VCF) (Danecek et al. 2011) file for the genotypic data of founders and sampled offspring and a text file for the pedigree information.

**Fig. 1.**
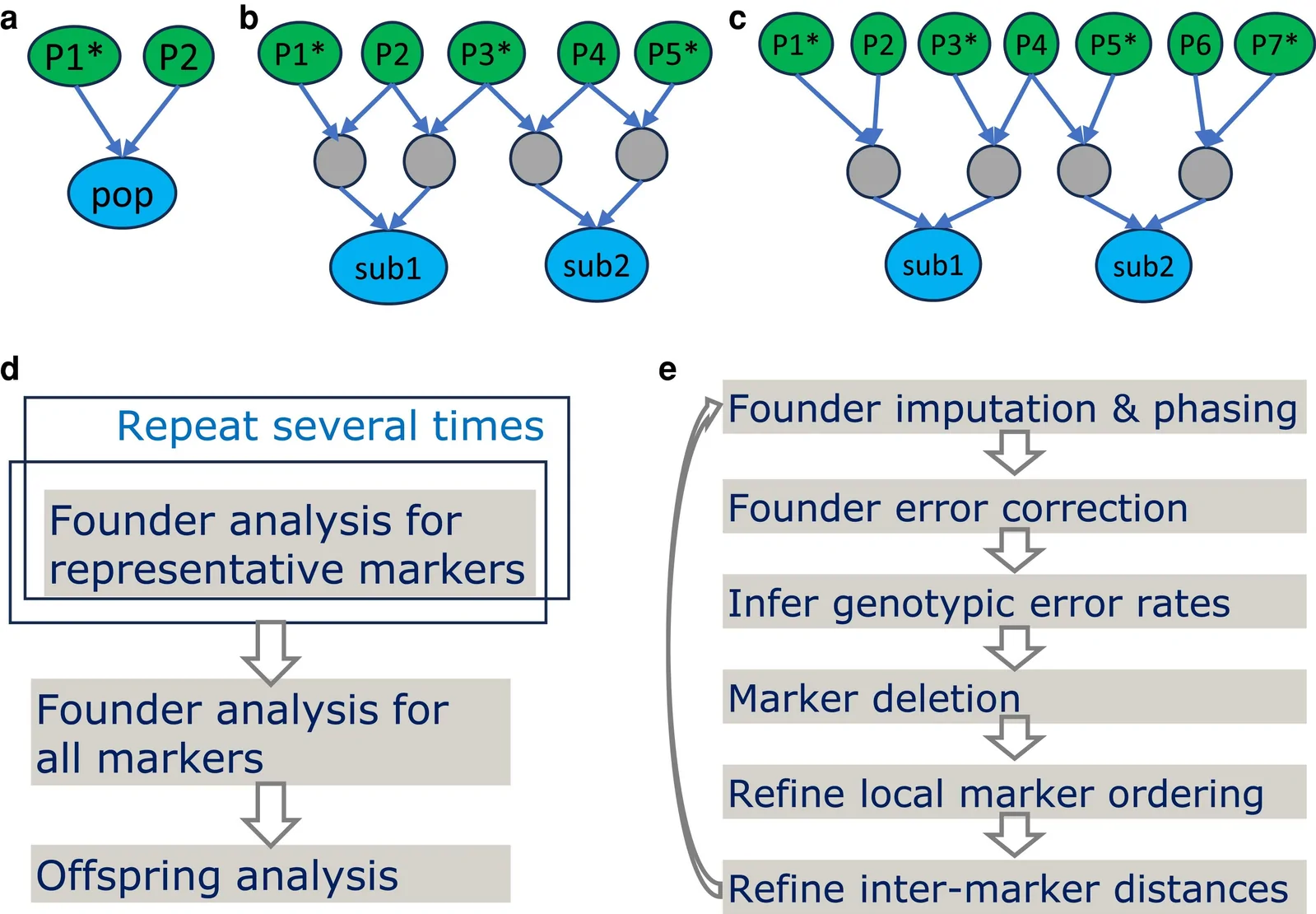
An overview of MagicImpute. a to c) Three example target populations with increasing number of outbred founders, which were used for benchmarking MagicImpute against previous methods. The superscript ^*^ denotes the founders with all genotypes missing. d) The workflow of MagicImpute. e) The workflow of one-time founder analysis.

Figure 1d shows the workflow of MagicImpute for a subdivided population, consisting of founder and offspring analyses. Marker binning is performed only if the average number of markers per bin is greater than the default 1.5, so that successive markers with distances less than the default 0.001 cM are binned. In the case of marker binning, the founder analysis is first performed for representative markers and then for all markers. To increase the robustness of founder genotype estimation, the founder analysis is repeated when it is challenging to find the global optimal imputation. By default, founder analysis is repeated from 3 to 6 times in the following situations: founders are partitioned into at least 2 blocks, and either founders are outbred or there is at least 1 founder with a large missing fraction (>0.6). While repeating we keep track of the repeat with the largest posterior probability and stop repeating if there are at least 2 other repeats satisfying either the log posterior probability difference with the largest one is less than ln(10) or the founder genotype difference is less than 0.1%.

The notations for describing the MagicImpute model and algorithm are listed in Supplementary Table S1. The MagicImpute algorithm builds on the hidden Markov model framework described in detail in Zheng et al. (2014, 2015, 2018a, 2018b), consisting of 3 components: initial, transition, and emission probabilities. The initial and transition probabilities describe the hidden origin process along chromosomes and are calculated by a recursive algorithm using pedigree information (Zheng et al. 2014, 2018b). The emission probability describes the genotypic data model (Zheng et al. 2015, 2018a). In the following, we first extend the data model of Zheng et al. (2018a) to account for additional types of errors. Then we describe each substep of the founder analysis in Fig. 1e, whereas MagicImpute_mma contains only the first substep. After briefly explaining the offspring analysis, we describe the evaluation of MagicImpute by simulations and real data.

### The genotype model

The genotype model calculates the probability of observed genotypic data given hidden states, which is also termed emission probability in the hidden Markov model (HMM). We assume markers are biallelic. For offspring *i* at marker *t*, we denote by $y_{ti}$ the genotypic data, $\epsilon_{ti}$ the allelic error probability, and $z_{ti}$ the hidden true offspring genotype. For founder *f* at marker *t*, we denote by $y_{t}^{f}$ the genotypic data and $h_{t}^{\;f}$ the hidden founder haplotype accounting for missing data and unknown phases. In addition, let $y_{t}^{\Omega }=\{y_{t}^{f}{\}}_{\;f\in \Omega }$ be the genotypic data for a subset *Ω* of founders at marker *t*, and $h_{t}^{\Omega }=\{h_{t}^{f}{\}}_{\;f\in \Omega }$ the hidden haplotypes, where Ω=F denotes the full set. We denote by $\epsilon^{F}$ the allelic error probability for all founders and set $\epsilon^{F}=0.005$ by default.

**Called genotype (GT):** We first consider that $y_{ti}$ is represented by a called genotype, corresponding to GT in the FORMAT field of a VCF file. The likelihood $P(y_{ti}|z_{ti},\epsilon_{ti})$ is determined by an error model. We assume a random allelic error model: typing errors occur independently for each allele, and the resulting allele is the other if an error occurs. We obtain the marginal likelihood $l_{ti}=P(y_{ti}|x_{ti},\epsilon_{ti},\epsilon^{F},h_{t}^{F})$ by summing out the hidden variable $z_{ti}$


(1) $$l_{ti}=\sum_{z_{ti}}P(y_{ti}|z_{ti},\epsilon_{ti})P(z_{ti}|h_{t}^{F},x_{ti},\epsilon^{F}),$$

where $x_{ti}$ is the hidden ancestral origin state, and the prior probability $P(z_{ti}|h_{t}^{F},x_{ti},\epsilon^{F})$ is given in Supplementary Table S2 of Zheng et al. (2015). Here an ancestral origin state denotes a pair of ancestral origins, corresponding to the maternally and paternally derived alleles, respectively. The possible ancestral origins are given by the set of founder labels if founders are inbred, and they are given by the set of founder gamete labels if founders are outbred (Zheng et al. 2015).

To reduce the number of error parameters, the allelic error probability $\epsilon_{ti}$ is modeled in terms of marker-specific error probability $\epsilon_{t}$ and offspring-specific error probability $\xi_{i}$,


(2) $$1-\epsilon_{ti}=(1-\epsilon_{t})(1-\xi_{i}),$$

assuming that marker-specific errors occur independently of offspring-specific errors. The error probabilities $\epsilon_{t}$ and $\xi_{i}$ will be estimated.

**Allelic depth (AD):** We next consider that $y_{ti}$ is represented by allelic depths in GBS data, corresponding to AD in the FORMAT field of a VCF file. We denote $y_{ti}=(r_{0},r_{1})$, where $r_{0}$ and $r_{1}$ are the numbers of reads for alleles 0 and 1, respectively. The read counts are modeled by the following binomial distributions:


(3) $$\begin{array}{cc} P((r_{0},r_{1})|z^{\prime }=0|0,\eta_{t}) & \propto (1-\eta_{t}{)}^{r_{0}}\eta_{t}^{r_{1}},\\ P((r_{0},r_{1})|z^{\prime }=0|1,\eta_{t}) & \propto q_{t}^{r_{0}}(1-q_{t}{)}^{r_{1}},\\ P((r_{0},r_{1})|z^{\prime }=1|0,\eta_{t}) & =\;\;\;P((r_{0},r_{1})|z^{\prime }=0|1,\eta_{t}),\\ P((r_{0},r_{1})|z^{\prime }=1|1,\eta_{t}) & \propto \eta_{t}^{r_{0}}(1-\eta_{t}{)}^{r_{1}}, \end{array}$$

conditional on hidden true genotype $z^{\prime }$ (Xie et al. 2010; Miao et al. 2018). Here phased genotype $z^{\prime }$ is given in format of $a_{0}|a_{1}$, where $a_{0}$ (or $a_{1}$) denotes alleles 0 or 1. $\eta_{t}$ denotes sequencing base error probability and $q_{t}$ denotes allelic balance given the true $z^{\prime }$ being heterozygous at marker *t*; $q_{t}=1/2$ corresponds to no allelic bias.

To account for the variation of allelic balance among offspring, $q_{t}$ is further modeled by a beta distribution


(4) $$q_{t}∼Beta(\alpha ,\beta ),\;\alpha =\mu_{t}/\phi_{t},\;\beta =(1-\mu_{t})/\phi_{t}$$

where shape parameters *α* and *β* are reparameterized by mean $\mu_{t}$ and overdispersion $\phi_{t}$. Thus, allelic depths $y_{ti}$ is modeled by a beta-binomial distribution that approaches the binomial model with $q_{t}=\mu_{t}$ if $\phi_{t}$ goes to 0.

We introduce $\epsilon_{ti}$ to account for depth-independence errors such as misalignment, and the likelihood is given as follows:


(5) $$P(y_{ti}=(r_{0},r_{1})|z_{ti},\epsilon_{ti},\eta_{t},\mu_{t},\phi_{t})=\sum_{z_{ti}^{\prime }}P(y_{ti}|z_{ti}^{\prime },\eta_{t},\mu_{t},\phi_{t})P(z_{ti}^{\prime }|z_{ti},\epsilon_{ti}),$$

where $P(z_{ti}^{\prime }|z_{ti},\epsilon_{ti})$ is similar to $P(y_{ti}|z_{ti},\epsilon_{ti})$ in equation (1) and $\epsilon_{ti}$ is given by equation (2). The final marginal likelihood is given by equation (1) with $P(y_{ti}|z_{ti},\epsilon_{ti})$ replaced by $P(y_{ti}|z_{ti},\epsilon_{ti},\eta_{t},\mu_{t},\phi_{t})$.

**Prior distribution:** At each marker locus, there are 2 error parameters, $\xi_{i}$ and $\epsilon_{t}$ for called genotypes, and 5 parameters $\xi_{i}$, $\epsilon_{t}$, $\eta_{t}$, $\mu_{t}$, and $\phi_{t}$ for allelic depths. We assign a weakly informative Beta prior distribution Beta(1.01,1.01) for $\mu_{t}$ such that the distribution is symmetric at 0.5 (no allelic bias). We assign a Beta distribution $\text{Beta}(1,1/\bar{m}-1)$ for each of the error parameters $\xi_{i}$, $\epsilon_{t}$, and $\eta_{t}$ and a prior exponential distribution with mean $\bar{m}$ for $\phi_{t}$, where $\bar{m}$ is the corresponding average over markers in the previous iteration during founder analysis.

### Founder imputation

To increase computational efficiency, we partition a set of founders into blocks such that each block must be a subset of the founders of a subpopulation, and the block size must be no greater than a prespecified upper bound $N_{b}$. The founder partition is randomized in each iteration. We first sample a subpopulation and then sample a block of founders from the subpopulation without replacement and repeat until there are no founders left. The sampling of a subpopulation is weighted by the total number of distinct offspring descended from the founders of the subpopulation.

The founder imputation starts from initializing founder haplotypes, where missing founder genotypes are randomly imputed and heterozygous genotypes for outbred founders are randomly phased. Specifically, let $h^{\Omega_{b}}=\{h_{t}^{\Omega_{b}}{\}}_{t}$ be the haplotypes for the *b*th subset of founders at all loci. We impute $h^{\Omega_{b}}$ using the algorithm of Zheng et al. (2018a), conditional on the current haplotypes in all the other blocks, and loop over each subset in each iteration. The founder imputation stops if the likelihood does not decrease for several successive iterations.

In each iteration, marker *t* is detected to be monomorphic in founders if there is only 1 allele in the current founder haplotypes $h_{t}^{F}$. In the initial 5 iterations, the observed founder genotypic data $y_{t}^{F}$ at each monomorphic marker *t* will be set to missing, aiming to reduce false detection because of errors in $y_{t}^{F}$. After 5 iterations, the monomorphic markers detected in each iteration are deleted.

### Founder error correction

In contrast to estimating the offspring error rates, we fix founder error rate $\epsilon^{F}$ to a small value (0.005 by default) and perform error correction. This is because offspring analysis is conditional on estimated founder haplotypes and remaining erroneous genotypes in founders can greatly decrease the estimation accuracy of offspring genotypes.

In each iteration, founder error correction is performed in several steps, and it stops in the subsequent iterations if the number of corrections is 0. We first estimate the hidden ancestral origin states $x_{ti}$ using the HMM Viterbi algorithm (Rabiner 1989; Zheng et al. 2015). Second, we obtain the best offspring genotypes by combining the estimated $x_{ti}$ and the current founder haplotype $h^{F}$. Third, we perform single site genotype calling if input data are allelic depths and compare the results with the estimated best genotypes. Here single site genotype calling is based on equations (1) and (5), where markers are assumed to be independent. Finally, at each marker, for each founder, we calculate the number of mismatches for each possible founder genotype and perform correction if it reduces at least 2 mismatches.

To minimize false corrections, flagged founder genotypes are set to missing, forcing imputation in subsequent iterations in the founder analysis (Fig. 1e).

### Offspring error estimation

We first estimate the hidden ancestral origin states $x_{ti}$ using the HMM Viterbi algorithm (Zheng et al. 2015; Rabiner 1989). Then we estimate each error parameter alternately, conditional on all the other parameter values. For example, at each marker *t* we estimate $\epsilon_{t}$ by maximizing the conditional posterior distribution $\prod_{i}l_{ti}p(\epsilon_{t})$, where $p(\epsilon_{t})$ is the prior distribution and $l_{ti}$ is given by equations (1) and (5). Similarly, we estimate the other marker-specific parameters $\eta_{t}$, $\mu_{t}$, and $\phi_{t}$ for allelic depths. Analogously, we estimate $\xi_{i}$ by maximizing the posterior distribution $\prod_{t}l_{ti}p(\xi_{i})$.

We delete markers with too large error rates after 5 iterations. For each marker-specific error parameter, a marker is labeled if its error rate is larger than the corresponding hard threshold, or if its error rate is outlier and is larger than the corresponding soft threshold. Similarly, an offspring is labeled if its error rate is larger than the hard threshold of $\xi _i$, or if its error rate is outlier and is larger than the soft threshold of $\xi _i$. The set of labeled markers is deleted, whereas the set of labeled offspring is temporarily excluded in the founder imputation and map refinement. The default hard thresholds for $\xi_{i}$, $\epsilon_{t}$, $\eta_{t}$, $\mu_{t}$, and $\phi_{t}$ are 0.25, 0.25, 0.05, 0.9, and 1.0, respectively; and the soft thresholds are 0.025, 0.025, 0.01, 0.67, and 0.01, respectively. An additional procedure for $\mu_{t}$ is performed to label markers with too small allelic balances. Outliers are detected based on the interquartile range using Tukey’s fence 2 (Tukey 1977). According to our preliminary simulation studies, the commonly used fence 1.5 resulted in a few too many deletions, while the far outlier fence 3 resulted in too few deletions. The error estimation stops if the change of average error rates is very small.

In short, markers with an error rate being greater than its soft threshold are deleted only if they are also outliers, and thus the soft threshold–based marker deletion can be disabled by setting Tukey’s fence to be infinitely large. In comparison, markers with an error rate being greater than its hard threshold are always deleted, and the hard threshold–based marker deletion can be disabled by setting hard thresholds to be infinitely large.

### Marker deletion

Besides the marker deletion based on the estimated error rates, we perform marker deletion using Vuong’s closeness test, a likelihood ratio–based test that can be used for comparing 2 non-nested models (Vuong 1989). Specifically, we first calculate Vuong’s test statistic for each marker with the alternative hypothesis that the marker is removed. Then we remove markers using significance level 0.01. The level is somewhat arbitrary, and we did not observe a large effect of multiple testing in the simulation studies. The marker deletion stops if there is no marker deleted in the current iteration.

### Map refinement

The map refinement includes refining local marker ordering and intermarker distances, which has been described in detailed by Zheng et al. (2019). It has been rearranged from the map construction package MagicMap (Zheng et al. 2019), so that the input marker map in MagicImpute can either be a physical map or be replaced by an optional map file. If the map file is provided by the output of MagicMap, it also contains a list of nearest markers for each marker. The neighborhoods can be used for neighbor-based order refining, which is much more effective than the default random order refining (Zheng et al. 2019). Thus, the order refinement is not performed by default if the optional map file is not provided.

### Offspring analysis

The offspring analysis has been described (Zheng et al. 2018a) under the same assumption: offspring are independent conditional on the estimated phased founder genotypes ${\hat{h}}^{F}$. For each offspring, we first calculate the marginal posterior probability $P(x_{ti}|\cdot )$ at each marker using the HMM forward–backward algorithm (Rabiner 1989), and then calculate the marginal genotype probability $P(z_{ti}|\cdot )=\sum_{x_{ti}}P(z_{ti}|{\hat{h}}_{t}^{F},x_{ti},\epsilon^{F})P(x_{ti}|\cdot )$. A missing genotype is imputed or a observed genotype is corrected if its maximum posterior genotype probability of $z_{ti}$ is greater than the default threshold 0.9.

As a metric for imputation accuracy at each marker, we calculate squared allelic correlation based on posterior genotype probability $P(z_{ti}|\cdot )$, that is, the allelic R-squared (AR2) generated by Beagle 4.1 (Browning and Browning 2016). We do not calculate the dosage R-squared (DR2) generated by Beagle 5 and the later versions, because it assumes the Hardy–Weinberg equilibrium that generally does not hold for experimental crosses (Browning et al. 2018).

### Evaluation by simulation

We evaluated MagicImpute by 3 groups of simulations. First, we simulated 3 populations with increasing number of outbred founders (Fig. 1a to c), aiming to benchmark MagicImpute against previous software such as Beagle 5.5 (Browning et al. 2018), MagicImpute_mma (Zheng et al. 2018a), and GBScleanR (Furuta et al. 2023). The subpopulations of a benchmarking population had equal sizes. For a benchmarking population with a given size, we simulated 3 types of genotypic data: called genotypes, GBS with allelic bias but no overdispersion, and GBS with both allelic bias and overdispersion. For the sake of comparison, we deleted monomorphic markers prior to imputation. Second, we simulated called genotypes in *n*-way recombinant inbred lines (RILs) (n= 2, 4, 8, and 16) by 6 generations of selfing, aiming to evaluate founder genotype estimation with or without repeating; each population was simulated with 2 sizes: 200 and 500. Third, we simulated sequence data in heterozygous populations F2 and CP, aiming to evaluate the estimation of error parameters; each population was simulated with sizes ranging from 5 to 1,000. For all groups of simulations, we assumed 5 linkage groups with genetic length 100 cM for each. The number of markers per linkage group was set to 200 in the first 2 groups of simulations, and it ranged from 100 to 2,000 in the last group. Data simulation has been described in previous studies (Zheng et al. 2015, 2018a).

The simulation parameters for genotype missing were specified as follows. Independently for each marker, a true founder genotype was set to missing according to a Bernoulli distribution with probability parameter *p*, where *p* was sampled from beta distribution Beta(1, 1/${\bar{\;p}}_{f}-1$) with mean ${\bar{\;p}}_{f}$, and similarly for a true offspring genotype with ${\bar{\;p}}_{o}$. We set ${\bar{\;p}}_{f}={\bar{\;p}}_{o}=0.05$ for all groups of simulations except for the following. For the benchmarking populations, we set all genotypes for some founders to missing (Fig. 1a to c) and ${\bar{\;p}}_{o}=0.2$ for all offspring. For an RIL population with a given *n* founders and a given population size, we performed n+1 independent simulations and set all the first *k* founders’ genotypes to missing in the (k+1)th simulation, where k=0,…, *n*.

The simulation parameters for error rates were specified as follows. The genotypic error model in simulation was the same as the genotype model for imputation. For simplicity, offspring-specific error rate $\xi_{i}$ was not simulated. At marker *t*, $\epsilon_{t}$ for offspring was sampled from Beta(1, 99) with mean 0.01, and similarly $\epsilon_{t}^{F}$ for founders was sampled from Beta(1, 99). For simulating sequence data, $\eta_{t}$ was sampled from Beta(1, 499) with mean 0.002, $\mu_{t}$ was sampled from Beta(3, 3) with mean 0.5 (no allelic bias), and $\phi_{t}$ was sampled from an exponential distribution with mean 0.3. The Beta distributions for $\epsilon_{t}^{F}$, $\epsilon_{t}$, and $\eta_{t}$ were set such that their means were twice as large as the default values of MagicImpute (see Table 1). In addition, the number $D_{it}$ of reads (read depth) for offspring *i* at marker *t* was sampled from a Poisson distribution with mean $D_{t}$, where $D_{t}$ was sampled from an exponential distribution with mean $\bar{D}$. Here $\bar{D}$ was set to 20 for the benchmarking populations and set to 5, 10, 20, and 40 for the heterozygous populations in the last group of simulations.

**Table 1.** List of nested error models for sequence data.

Model	Size	Parameters to be estimated	Incremental parameter
M0	0	Null	Null
M1	1	$\epsilon_{t}$	$\epsilon_{t}$ marker-specific offspring error
M2	2	$\epsilon_{t}$ , $\mu_{t}$	$\mu_{t}$ measure of allelic bias
M3	3	$\epsilon_{t}$ , $\mu_{t}$, $\phi_{t}$	$\phi_{t}$ allelic overdispersion
M4	4	$\epsilon_{t}$ , $\mu_{t}$, $\phi_{t}$, $\eta_{t}$	$\eta_{t}$ sequence base error
M5	5	$\epsilon_{t}$ , $\mu_{t}$, $\phi_{t}$, $\eta_{t}$, $\xi_{i}$	$\xi_{i}$ individual-specific offspring error

The default values for parameters that are not estimated: $\epsilon_{t}=0.005$, $\mu_{t}=0.5$, $\phi_{t}=0$, $\eta_{t}=0.001$, and $\xi_{i}=0$. The size of a model is defined as the number of error parameters in the model.

In the first group of benchmarking studies, GBScleanR and MagicImpute_mma were performed using parallel threading with 5 threads, whereas MagicImpute was performed using parallel processing with 5 workers in the case of 5 linkage groups. All analyses were performed using a desktop computer with Intel Xeon W-2235 CPU @ 3.80 GHz and 64 GB RAM memory.

### Evaluation by real data

We evaluated the founder genotype imputation by the sorghum MAGIC (Ongom and Ejeta 2018) with 29 founders and 194 offspring, where 10 of 29 founders were unavailable for sequencing. The population was developed by crossing 19 founders with 10 random sterile plants out of a genetic male sterile source, followed by 10 generations of random mating and then 7 generations of selfing. Unexpectedly, around 4% genotypes were heterozygous, and they were set to missing in our study. The genotypic data were filtered for markers with minor allele frequency (MAF) MAF≥0.05, founder missing fraction $p_{f}<0.8$, and offspring missing fraction $p_{o}<0.8$, resulting in 44,767 markers. After filtering, the missing fraction in the 19 genotyped founders was 0.11. Since the breeding pedigree was unknown, MagicImpute used the prior average number 11 of recombination breakpoints per sampled offspring per Morgan as an input, according to the mating design.

In addition, we examined a series of nested error models (Table 1) by stepwise inclusion of the inferring error parameters $\epsilon_{t}$, $\mu_{t}$, $\phi_{t}$, $\eta_{t}$, and $\xi_{i}$. We evaluated the impact of the error models by sequence data in 2 heterozygous biparental populations: rice F2 (Furuta et al. 2017) and apple CP (Gardner et al. 2014) with the number of offspring being 813 and 87, respectively. The genotypic data for the rice F2 were filtered for markers with MAF≥0.1 and $p_{o}<0.95$, resulting in 8,372 markers; and the genotypic data for the apple CP were filtered for markers with MAF≥0.1 and $p_{o}<0.8$, resulting in 48,713 markers.

MagicImpute was performed with and without inferring intermarker distances for each error model. The input physical maps were transformed into genetic maps using the constant recombination rates of 4.0 and 2.5 cM/Mbp for rice (Furuta et al. 2017) and apple (Gardner et al. 2014; Di Pierro et al. 2016), respectively. The imputation results were evaluated in 2 quantities: genotypic discordance was calculated by randomly masking 10% input genotypic data with read depth ≥10 and using them as pseudo-true genotypes; the number of recombination breakpoints is estimated by haplotype reconstruction using the imputed genotypic data. Here the haplotype reconstruction was performed using MagicReconstruct, a Julia version of MagicReconstruct_mma (Zheng et al. 2015) that was implemented in Mathematica.

See Supplemental File S1 for technical details on data preparation and analyses.

## Results

### Comparison with previous methods

First of all, it is noteworthy to mention that Beagle 5.5 works only for called genotypes, whereas GBScleanR works only for allelic depths. For fair comparisons, MagicImpute was set such that there were no markers deleted, and all output founder genotypes were imputed and phased.

In comparison with MagicImpute and MagicImpute_mma, Beagle does not correct input called genotypes and thus resulted in large genotypic discordances in both founders and offspring; see the left panels in Fig. 2 and Supplementary Figs. S1 and S2. In addition, Beagle does not incorporate pedigree information and thus is incapable of imputing missing founders. On the other hand, Beagle was computationally superefficient, and it was 2 to 3 orders of magnitude faster than the others (Fig. 2 and Supplementary Fig. S1).

**Fig. 2.**
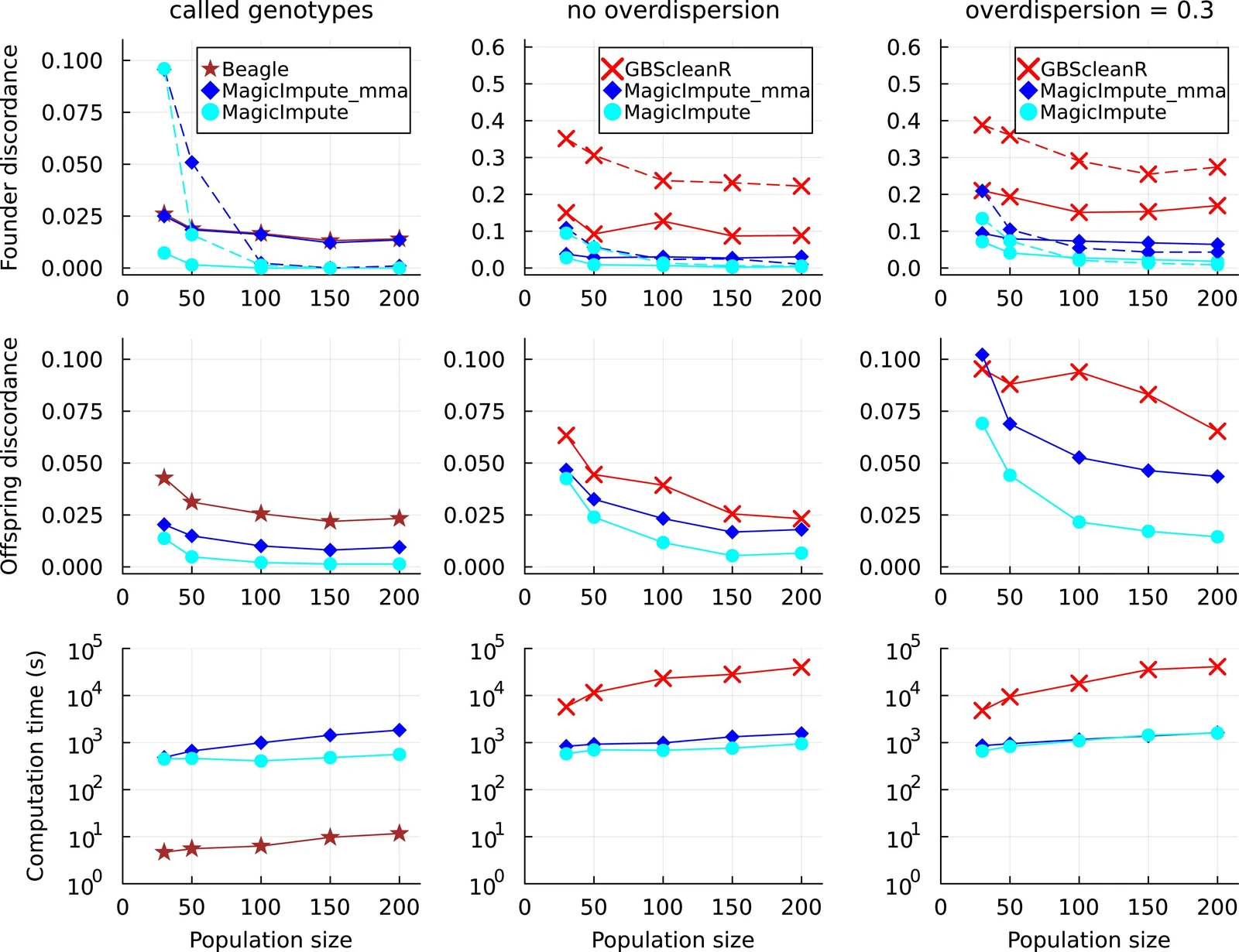
Comparisons of MagicImpute with MagicImpute_mma, Beagle, and GBScleanR in the simulated populations with 5 founders (Fig. 1b). Beagle worked only for called genotypes in the left panel and GBScleanR worked only for GBS data in the middle and right panels. In the top panels, the dashed lines refer to the founders with all genotypes missing, and the solid lines refer to the other founders. Note that there are no dashed lines for Beagle in the first panel since Beagle was incapable of imputing missing founders.

In contrast to MagicImpute_mma, GBScleanR was developed to account for allelic bias. GBScleanR resulted in slightly smaller genotypic discordances than MagicImpute_mma in the benchmarking CP populations without overdispersion (Supplementary Fig. S1). However, it underperformed in the benchmarking populations with increased numbers of founders (Fig. 2 and Supplementary Fig. S3). In addition, GBScleanR resulted in much larger fractions of nonimputed genotypes and phasing error rates in all benchmarking populations (Supplementary Figs. S1 to S3).

MagicImpute consistently resulted in smaller genotypic discordances and phasing error rates than the other methods for all 3 types of genotypic data (Figs. 2 and Supplementary Figs. S1 to S3). In comparison with GBScleanR and MagicImpute_mma, the computation time and peak memory usage of MagicImpute increased much more slowly with the number of founders (Supplementary Fig. S3). For the benchmarking population with 7 founders and 1 linkage group, GBScleanR took 62 h, much longer than the 0.29 h of MagicImpute, and MagicImpute_mma used 18 Gibibyte (GiB) memory, much more than the 3.5 GiB of MagicImpute (Supplementary Fig. S3).

### Evaluation by simulated RILs

Figure 3 shows that founder imputation was quite accurate even when some or all founders’ genotypes were completely missing in the 16-way RILs of size 200. The founder genotypic discordance was close to 0 when the number of missing founders was no larger than 5, and then it increased roughly with the number of missing founders (Fig. 3a). The fluctuation was mainly because the one-time founder analysis algorithm was very greedy and usually finished within around 10 iterations such that it sometimes converged to local maxima. Further examinations showed that the founder discordances often occurred in blocks, and these haplotype blocks were often switched with each other. Repeating founder imputation several times reduced the magnitude and fluctuation of discordances at the cost of computational time (Fig. 3).

**Fig. 3.**
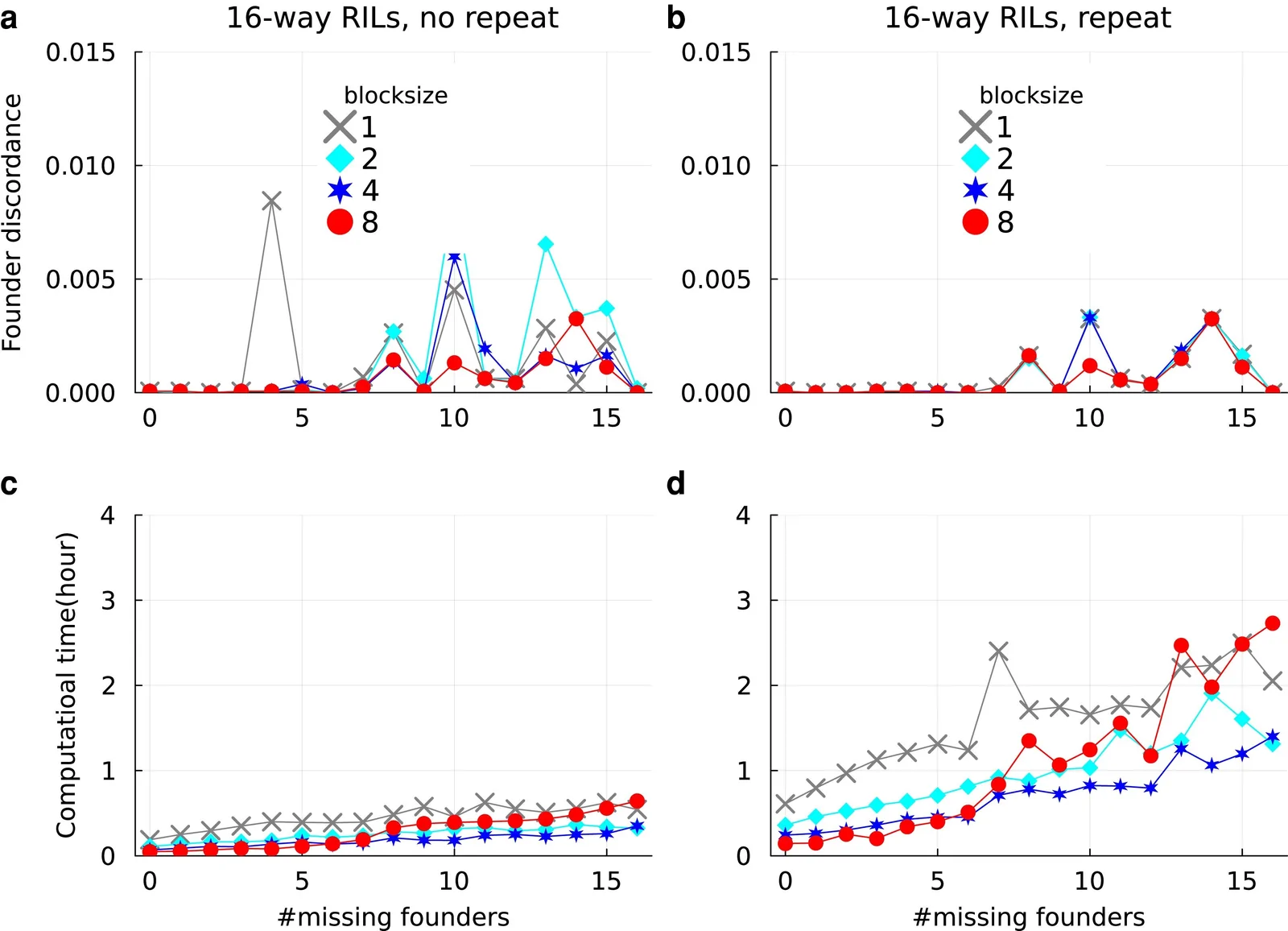
Founder imputation in the simulated 16-way RILs with population size 200. The *x*-axis denotes the number of founders with all genotypes missing. The left panels (a & c) and the right panels (b & d) show the results without and with repeating founder analyses, respectively.

The founder genotypic discordance decreased with population size. As shown in Supplementary Fig. S4 for the 16-way RILs, the founder discordances for population size 500 were generally smaller than those for population size 200, and they were mostly 0 when repeating founder imputation. Whether founder imputation was repeated or not, the founder discordances for the *n*-way RILs with n≤8 were mostly 0 except for block size $N_{b}=1$. Therefore, repeating founder imputation is not very necessary if the size of each subpopulation is large with respect to the number of founders for the subpopulation.

The founder genotypic discordance depended little on block size $N_{b}$ ( Fig. 3 and Supplementary Fig. S4), except that $N_{b}=1$ often did not work well (Supplementary Fig. S5). However, the computational time decreased with $N_{b}$ up to 4, and for $N_{b}=8$ it increased quickly when the number of missing founders was larger than 6 (Fig. 3c-d and Supplementary Fig. S4c-d). By default, MagicImpute set $N_{b}=2$ for outbred founders and $N_{b}=4$ for inbred founders.

### Evaluation by simulated F2 and CP

The estimation accuracy of the error rates, the correlation between estimated and true values, generally increased with read depth and population size (Fig. 4). As shown in Supplementary Fig. S6, the estimation bias, the average of the estimates minus the true values, decreased asymptotically into 0 with population size and read depth. The negative estimation biases were mainly because of markers with large error rates being deleted, whereas the positive estimation biases were mainly because of the large prior means. As expected, the estimations in small populations of size <100 were not accurate and biased. The error estimations for the F2 were more accurate and less biased than that for the CP, probably because the latter retained more erroneous founder genotypes after imputation (Fig. 5).

**Fig. 4.**
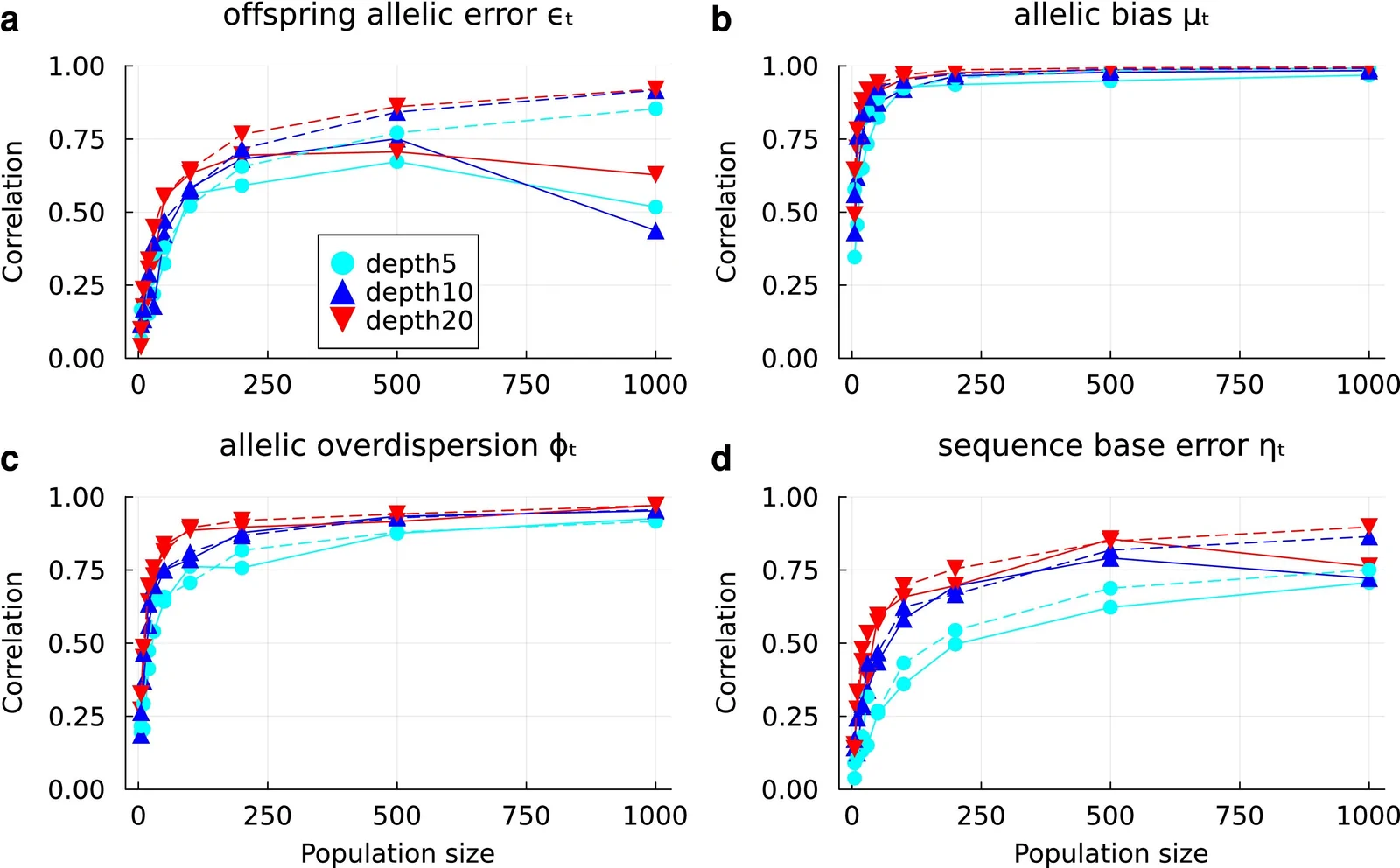
Estimation accuracy of the marker-specific error parameters in the simulated F2 (dashed lines) and CP (solid lines). The accuracy was measured by the correlation between estimated and true values among markers. For the sake of visualization, results for depth 40 are not shown. a-d) refer to offspring allelic error, allelic bias, allelic overdispersion, and sequence base error, respetively.

**Fig. 5.**
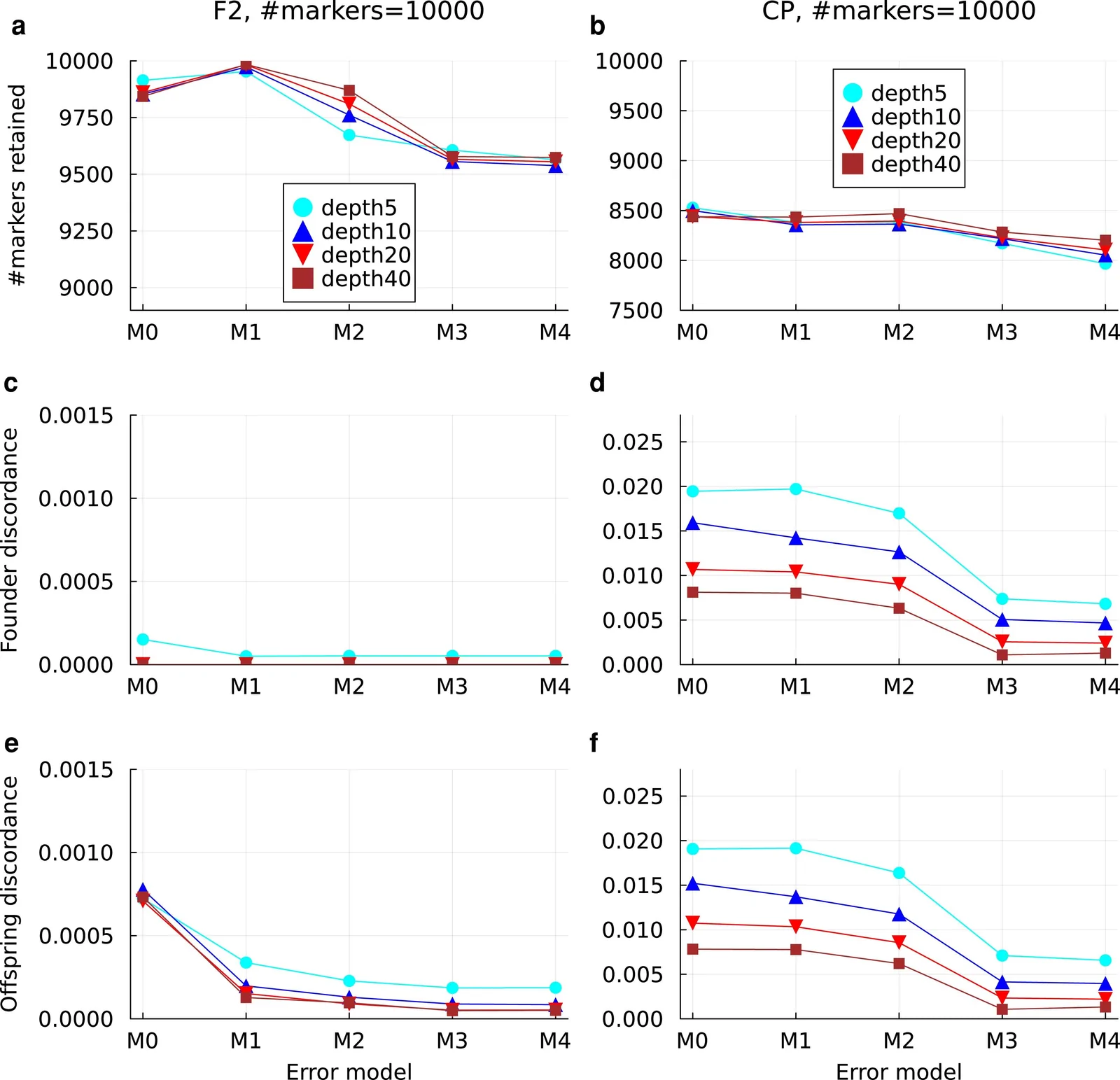
Genotype estimation in simulated F2 (a, c, and e) and CP (b, d, and f). The results were obtained with the default founder error correction and the soft and hard threshold–based marker deletion. The error models are defined in Table 1. The discordance refers to the fraction of estimated genotypes being different from true values.

There were 3 actions affecting the impact of the nested error models (Table 1) on genotypic discordance: the founder error correction, the soft threshold–based marker deletion, and the hard threshold–based marker deletion. Figure 5 shows the results with all the 3 default actions, and Supplementary Figs. S7 to S9 show the results obtained by forward stepwise disabling the actions. In the simulated F2, the hard threshold–based deletion removed almost all founder genotypic errors, and the subsequent soft threshold–based deletion and founder error correction were ineffective. In the simulated CP, the hard threshold–based deletion removed roughly half of founder genotypic errors (except null model M0); the subsequent soft threshold–based deletion greatly reduced genotypic errors for models M3 and M4, but they had essentially no effect for M0 and M2; and the further founder error correction reduced genotypic errors for all the models, meanwhile it increased the number of retained markers. Briefly, a larger error model resulted in smaller genotypic discordances by deleting more markers with large estimated error rates.

On the other hand, the nested error models had negligible impact on the estimated number of recombination breakpoints, except that the null model resulted in more breakpoints; see Supplementary Fig. S10 with all the 3 actions. The estimated numbers of breakpoints for the non-null error models were almost independent of read depth (Supplementary Fig. S10a and b), but they asymptotically increased with the number of markers to the prior expected values (Supplementary Fig. S10c and d). Stepwise disabling the 3 actions resulted in no noteworthy differences with Supplementary Fig. S10.

Sensitivity analyses were performed for 2 options affecting marker deletion. The soft threshold–based marker deletion detected outlier markers according to the default Tukey’s fence 2. As shown in Supplementary Figs. S11 and S12, decreasing Tukey’s fence from 3 to 1.5 generally resulted in increasing marker deletions mainly for models M3 and M4, which in turn resulted in decreasing genotypic discordances in the CP but not in the F2. In addition, sensitivity analyses were performed for the default significance level 0.01 used in Vuong’s test-based marker deletion. As shown in Supplementary Figs. S13 and S14, increasing significance level from 0.001 to 0.05 resulted in slightly increasing marker deletions mainly for null model M0.

### Evaluation by sorghum MAGIC

The genotypic discordance for the 19 genotyped founders ranged from 0.026 to 0.34 with mean 0.15, whereas the discordance for offspring was 0.0068. After imputation, the average missing fraction was 0.25 for the 10 ungenotyped founders and 0.068 for the other 19 founders (Fig. 6a), whereas it was 0.018 for the offspring. The missing genotypes in the 10 ungenotyped founders did not occur at random but in segments, probably because these segments were not inherited by any offspring.

**Fig. 6.**
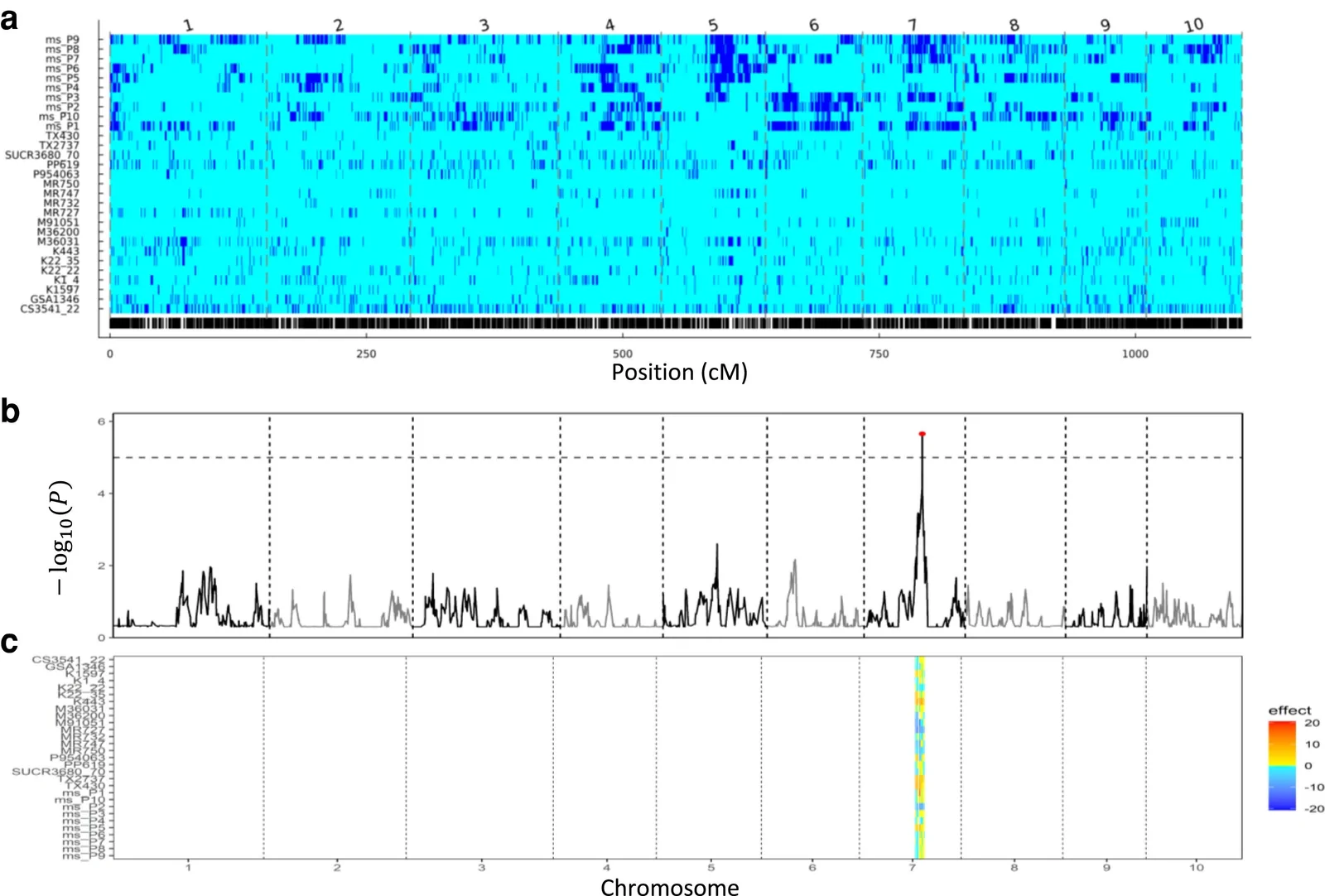
Founder imputation and QTL mapping in the real sorghum MAGIC. The dashed vertical lines indicate the chromosome boundaries. a) The missing pattern of founder genotypes after imputation. Blue and aqua denote missing and nonmissing, respectively. The founders from ms_P1 to ms_P10 were completely missing before imputation. The bottom vertical lines denote marker positions. b) QTL profile of plant height based on the posterior origin–genotype probabilities calculated from the imputed genotypic data. The horizontal dashed line denotes the threshold. c) The effect of founder origins at detected QTLs.

The large fraction of nonimputed founder genotypes had little effects on the estimated number of recombination breakpoints per offspring. The estimated genetic length was 1,103 cM (Supplementary Fig. S15a), slightly smaller than the previous estimations ranging from 1,192 to 1,369 cM (de Souza et al. 2021; Jin et al. 2021; Somegowda et al. 2022; Guden et al. 2023). In return, the estimated average breakpoint density was 13.1 per Morgan per offspring, slightly larger than the prior expected value 11 (Supplementary Fig. S15b).

We further evaluated imputation by QTL mapping for plant height. The QTL profile in Fig. 6b was obtained using the statgenMPP package (Li et al. 2022) with inputs being the posterior origin–genotype probabilities for all offspring and the average plant heights between years 2013 and 2014. In comparison with the previous genome-wide association study (GWAS) (Ongom and Ejeta 2018), the major QTL in chromosome 7 was recovered, whereas the 2 other minor QTLs in chromosomes 6 and 9 were absent in current study (Fig. 6b).

There are several possible reasons for the differences between current QTL mapping and the previous GWAS. The latter was based on the plant heights in the 2 yr rather than the average values, whereas our statgenMPP analysis was applied to the height means across the 2 yr. More likely, the population size 194 is not sufficient for accurately imputing the 29 founders, as shown in Fig. 6a. On the other hand, founder imputation allows for QTL mapping that in turn allows for estimating the effects of founder origins (Fig. 6c).

### Evaluation by rice F2 and apple CP

Figure 7 shows the effects of the nested error models (Table 1) in the real rice F2 and the apple CP. The genotypic discordances generally decreased with error model size except for M0 (Fig. 7a to d), although the decreases were marginal for the rice F2. In contrast to the simulation studies, the number of recombination breakpoints decreased with error model size (Fig. 7e and f). As shown in Supplementary Figs. S16 and S17, the excessive breakpoints mostly resulted from tiny segments, which might explain that the error models had only small effects on imputation accuracy.

**Fig. 7.**
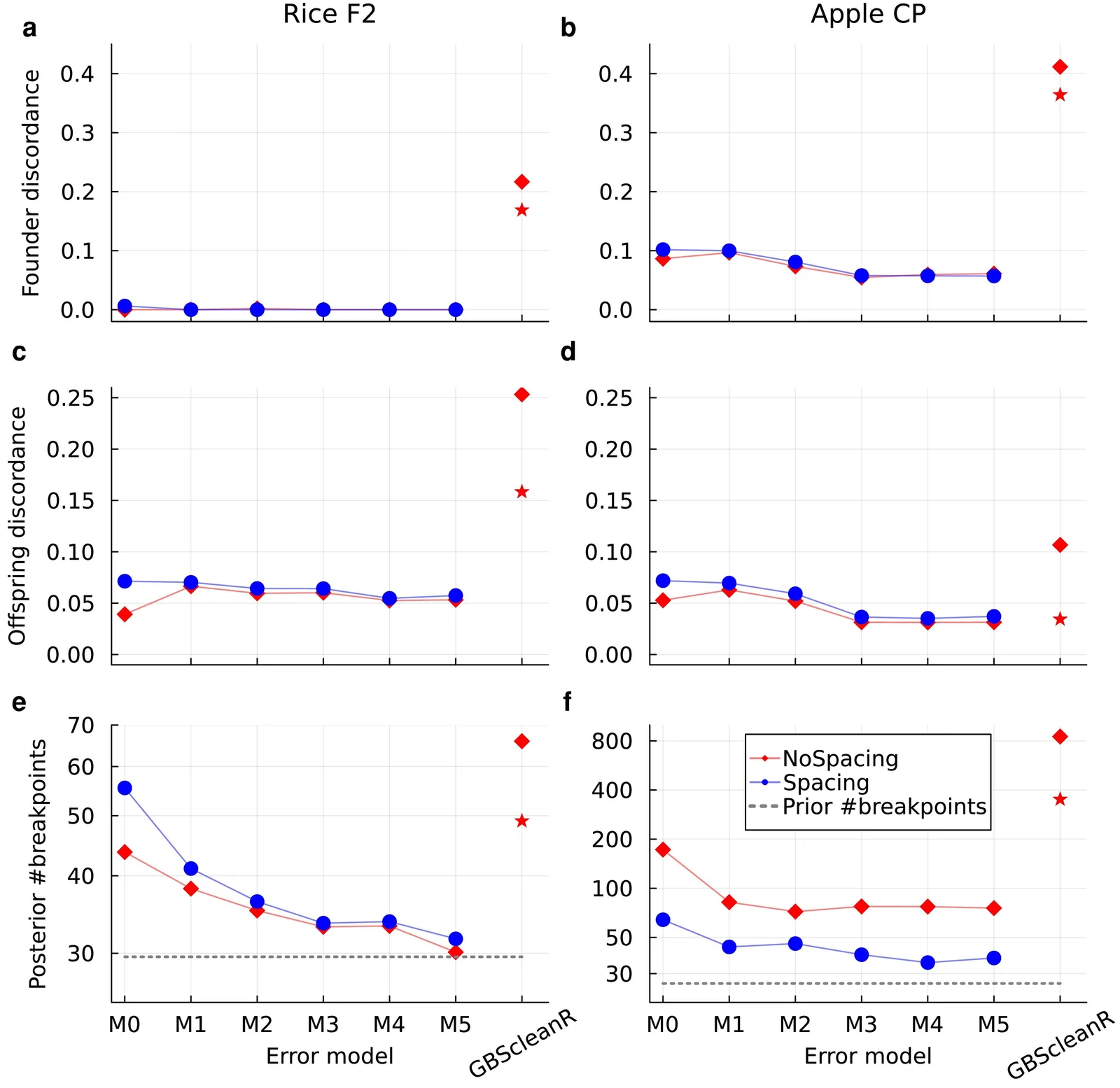
Impact of error models in the real rice F2 (a, c, and e) and apple CP (b, d, and f). The error models are defined in Table 1. The blue and red lines denote the results with and without inferring intermarker distances, respectively. The dotted lines in the bottom panels denote the prior number of recombination breakpoints per offspring. For GBScleanR, the red diamonds refer to the summaries based on all the markers, whereas the red stars refer to the summaries based on only the markers that remained by MagicImpute using model M5 without marker spacing.

To account for recombination rate varying along chromosomes, intermarker distances were inferred during genotype imputation. Broadly, it had negligible effects on genotypic discordance but a considerable influence on the number of breakpoints (Fig. 7). Specifically, it resulted in longer genomic lengths and more recombination breakpoints in the rice F2, but the differences diminished with increasing error model size (Fig. 7e). For the rice F2 using model M5, the estimated genetic length was 1,503 cM, falling in the range of previous estimations from 1,390 to 1,635 cM (Bai et al. 2010; Wen et al. 2020; Fekih et al. 2023); the estimated average breakpoint density was 2.1 per Morgan per offspring, similar to the prior expected value 2 (Supplementary Fig. S18).

In contrast, inferring distances resulted in fewer recombination breakpoints than those without inferring distances in the apple CP (Fig. 7f). However, the estimated number of breakpoints was still larger than the prior expectation. Consistently, the estimated genetic length using model M5 was 1,865 cM, longer than the 1 267 cM obtained by Di Pierro et al. (2016). Supplementary Figure S18 shows that chromosomes 9 and 15 had much longer genetic lengths and much more breakpoints than the others. This is probably because the input physical map for the apple CP has low quality (Di Pierro et al. 2016; Peace et al. 2019) and the errors due to marker misordering played a dominant role.

In the rice F2 and the apple CP, there were typically around 2 markers in a chromosome stretch of length 75 bp. The genotypic discordances and the number of breakpoints became larger when nearby markers were not binned during imputation (Supplementary Fig. S19), indicating that nearby co-segregating markers had large influence.

As shown in Fig. 7 and Supplementary Fig. S19, MagicImpute outperformed GBScleanR by deleting about half of erroneous markers during imputation, whereas GBScleanR retained all markers and resulted in much larger genotypic discordances and much more recombination breakpoints. Considering only the markers retained by MagicImpute in the rice F2, the offspring discordance for GBScleanR decreased from 0.25 to 0.16 (Fig. 7c), close to 0.12 obtained by Furuta et al. (2023) under stringent criteria of selecting markers for genotype masking (missing fraction <0.2 and minor allele frequency >0.4). Supplementary Figure S20 shows that MagicImpute performed much worse when marker deletion was disabled, but it still did mostly better than GBScleanR.

## Discussion

We have developed MagicImpute for genotype imputation in connected multiparental populations, especially increasing computational efficiency when there are a large number of founders. By partitioning founders into blocks, MagicImpute increases computation efficiency in 2 aspects. First, in each iteration the number of calculations for different founder haplotypes is much smaller than that of MagicImpute_mma, even though a few more iterations are required for MagicImpute. Second, conditional on the current founder haplotypes in each iteration, MagicImpute performs the standard HMM algorithms (Rabiner 1989; Zheng et al. 2015) for each subpopulation, whereas MagicImpute_mma analyzes the whole population jointly. The computational efficiency for MagicImpute is mainly determined by the block size $N_{b}$ and the numbers of founders for each subpopulation, rather than the total number of founders for the whole population for MagicImpute_mma.

The benchmarking studies have shown that MagicImpute was usually more accurate than Beagle 5.5 (Browning et al. 2018), MagicImpute_mma (Zheng et al. 2018a), and GBScleanR (Furuta et al. 2023), in terms of genotypic discordance and parental phasing. We chose Beagle 5.5 as a baseline for comparisons, but it is not the main competitor of MagicImpute. Beagle 5.5 shows an incapability to impute ungenotyped founders and to account for sequencing errors, although it is computationally efficient. In comparison with MagicImpute_mma and GBScleanR, MagicImpute scaled much better with the number of founders, allowing for integrating many bi- or multiparental populations. It is worth noting that accurate parental imputation and phasing are required for downstream haplotype reconstruction and QTL mapping.

MagicImpute is capable of imputing founders even when some or all founders’ genotypes are completely missing, as shown in the simulation studies. Few imputation methods have such capacity. AlphaFamImpute (Whalen et al. 2020) works for full-sib families with 2 outbred ungenotyped parents, and MagicImpute_mma works only for a limited number of ungenotyped founders. Assuming a genetically diverse population is derived a given number of generations ago, sequencing to imputation through constructing haplotypes (STITCH) (Davies et al. 2016) works for low-coverage sequences without requiring a reference panel. However, it needs a high-quality reference assembly. It is promising to further extend MagicImpute for genetically diverse populations by imputing virtual founders.

The evaluation by the real sorghum MAGIC has also shown the feasibility of QTL mapping after imputing ungenotyped founders, although a larger population size might be necessary for accurately imputing so many missing founders and possibly demonstrating the superior performance of QTL mapping over GWAS. Compared to GWAS, QTL mapping can also provide additional information such as the identification of potentially causal QTL haplotypes. However, a sophisticated QTL modeling for traits in multienvironments in multiparental populations is demanded.

MagicImpute has implemented various error models for sequence data, where each error parameter can be specified whether it is to be inferred or to be fixed to the default value. Our evaluations by simulation studies and real data have shown that a larger error model can increase imputation accuracy by deleting more erroneous markers. Miao et al. (2018) have shown that the Genotype-Corrector has larger correct rates of heterozygous genotype calls in F2 populations than those of Beagle and LinkImpute (Money et al. 2015). In comparison, GBScleanR (Furuta et al. 2023) retained all markers and resulted in low imputation accuracy.

In contrast to the consistent effects on imputation accuracy, the effects of error models on the estimated number of recombination breakpoints are different between the simulation studies and the evaluations by real data. The HMM framework of MagicImpute assumes independence of genotypic data, conditional on hidden ancestral states; and the differential effects are very likely because the assumption of conditional independence is valid in simulation studies, but it is violated for the real data. In the rice F2 and the apple CP, many reads span multiple markers within short sequence stretches. Apparently, the nonindependence of reads can be partly accounted for by increasing the number of error parameters, and partly accounted for by marker binning in MagicImpute. As shown in Supplementary Fig. S21, thinning markers by taking the first marker for each sequence stretch of length 75 bp prior to imputation greatly reduced the number of breakpoints (except for the apple CP with distance inference). A further extension to replace marker bins with multiallelic markers instead of representative markers is under development.

MagicImpute is suitable for cleaning data by deleting markers or offspring with large estimated error rates. If error parameters are not inferred, marker deletion mainly results from Vuong’s closeness test (Vuong 1989), which is controlled by the significance level. However, it is recommended to infer error parameters, and if so, the test-based marker deletion is not very necessary, because these markers usually have large error rates and then are deleted based on the hard thresholds. There are many optional parameters in MagicImpute, including marker binning, repeating founder analysis, and the soft and hard thresholds for each error parameter. However, the default values of these options have been carefully chosen based on extensive tests. When input data are allelic depths, Tukey’s fence instead of the soft thresholds is suggested for a fine control of marker deletion. See Supplementary Table S2 for a list of the main options of MagicImpute and Supplementary File S1 for guidance on calibrating them.

MagicImpute is also suitable for constructing a consensus genetic map in connected mapping populations, assuming the input physical map has high quality. The inflation of genomic length can be alleviated by marker deletion and error estimation; particularly, the individual specific error rate $\xi_{i}$ can account for different genotypic platforms and possible sample contamination. If the input physical map has low quality such as the apple CP, it is recommended to first construct a genetic map by the MagicMap (Zheng et al. 2019) and then refine it by MagicImpute.

MagicImpute has been developed and tested for (diploid) plant species, and it can be in principle applied to autosomal chromosomes of animal species such as the mouse collaborative cross population (Churchill et al. 2004). However, MagicImpute has not yet been implemented for sex chromosomes, even though MagicImpute_mma works for them.

In conclusion, MagicImpute is accurate for genotype imputation in connected multiparental populations with many subpopulations, and it is capable of imputing many missing founders provided there are a sufficient number of genotyped offspring, opening up new opportunities for downstream haplotype-based QTL mapping.

## Supplementary Material

iyag217_Supplementary_Data

iyag217_Peer_Review_History

## Data Availability

MagicImpute is implemented as a package in the RABBIT software written in the Julia language (Bezanson et al. 2017). RABBIT is available from the web site: https://github.com/biometris/RABBIT. There are also command line interfaces for running RABBIT in a Windows or Linux shell. The data simulations were performed using MagicSimulate in RABBIT, and the number of recombination breakpoints and the posterior origin–genotype probabilities were calculated from imputed genotypic data using MagicReconstruct in the RABBIT. The real data for the rice F2, the apple CP, and the sorghum MAGIC were downloaded based on the supplemental information of Furuta et al. (2017), Gardner et al. (2014), and Ongom and Ejeta (2018). Supplemental material available at GENETICS online.
