# Supplementary material for: Genotype imputation and error estimation in connected multiparental populations
Source: Genetics. 2026 Aug 17;234(2):iyag217. doi: 10.1093/genetics/iyag217 (PMC13643761; doi:10.1093/genetics/iyag217)
Supplement: Supplementary file 1 [file iyag217_peer_review_history.pdf]

GENETICS

Genotype imputation and error estimation in connected multiparental populations

Chaozhi Zheng, Eligio Bossolini, Nataša Formanová, Antje Rohde, Martin Boer, and Fred van Eeuwijk

NOTE: The reviews and decision letters are unedited and appear as submitted by the reviewers.
In extremely rare instances and as determined by a Senior Editor or the EIC, portions of a review may be redacted. If a review is signed, the reviewer has agreed to no longer remain anonymous.
The review history appears in chronological order.

Review Timeline:

Submission Date:	2025-10-08
Editorial Decision:	2025-11-21
Resubmission Received:	2026-03-20
Editorial Decision:	2026-04-15
Resubmission Received:	2026-06-12
Editorial Decision:	2026-06-27
Revision Received:	2026-07-10
Accepted:	2026-07-31

1st Editorial Decision: November 21, 2025

November 21, 2025

GENETICS-2025-308674
Genotype imputation and error estimation in connected multiparental populations

Dear Dr. Zheng:

Three experts in the field have reviewed your manuscript, and I have read it as well. While your manuscript is not currently acceptable for publication in GENETICS, we would welcome a substantially revised manuscript. Both reviewers have comments and concerns to be addressed in a revised manuscript. You can read their reviews at the end of this email. We look forward to receiving your revised manuscript. Please let the editorial office know approximately how long you expect to need for revisions.

Upon resubmission, please include:
1. A clean version of your manuscript;
2. A marked version of your manuscript in which you highlight significant revisions carried out in response to the major points raised by the editor/reviewers (track changes is acceptable if preferred);
3. A detailed response to the editor's/reviewers' feedback and to the concerns listed above. Please reference line numbers in this response to aid the editor and reviewers.

Your paper will likely be sent back out for review.

Additionally, please ensure that your resubmission is formatted for GENETICS
https://academic.oup.com/genetics/pages/general-instructions

Follow this link to submit the revised manuscript: Link Not Available

Sincerely,

Yun Li
Associate Editor
GENETICS

Approved by:
Hongyu Zhao
Senior Editor
GENETICS

Reviewer #1 :

This paper presents MagicImpute, a high-performance software tool for genotype imputation in connected multiparental populations. Written in Julia, it enhances a previous tool, MagicImpute_mma, by improving computational efficiency and robustness to sequencing errors. Key features include a novel algorithm for imputing missing founder genotypes, even when many are unsequenced, and a beta-binomial model to account for allelic bias and overdispersion. The method estimates error rates to filter problematic markers. Its accuracy was validated using extensive simulations and real datasets from sorghum, rice, and apple populations. The following points are raised out of my personal curiosity and are offered for the authors' consideration.

1. Threshold and marker-deletion sensitivity:
The current settings for soft/hard deletion thresholds and the default Tukey fence of 2 appear empirical while the authors explained that 1.5 tends to over-delete whereas 3 tends to under-delete. Besides, the iterative marker-deletion step based on the Vuong test (alpha = 0.01) is introduced without a systematic calibration. Therefore, I recommend adding a concise supplementary sensitivity analysis by performing a small grid scan over the combinations of hard thresholds, soft thresholds/Tukey fence values, and on/off use of the Vuong test. This addition would substantially improve reproducibility and strengthen the empirical justification of the pipeline's curation settings.
2. Insufficient and Unbalanced Benchmarking:
The current benchmarking of MagicImpute against existing methods, particularly GBScleanR, seems insufficient and unbalanced, preventing a clear assessment of the tool's practical advantages. Specifically, MagicImpute is shown to achieve lower genotypic disconcordance, but only after deleting approximately 45% of markers, whereas GBScleanR retained all markers. This constitutes an unequal comparison, essentially contrasting "high accuracy via aggressive filtering" with "high

marker retention." To enhance the benchmarking, I have the following suggestions.
A. Establish Fair Baseline Comparisons: It would be better if the authors could discuss more fully why their comparison is practically reasonable, useful, and informative. Alternatively, the authors could apply a uniform post-imputation quality filter to the GBScleanR output (e.g., matching the final marker density of MagicImpute) before comparing accuracy on the commonly retained markers. Either approach would be acceptable to me.
B. Expand Benchmarking Scope: Are there other applicable baseline tools beyond GBScleanR? If so, would the authors add them-or at least one previously strong model-to the comparison? If not, please add a paragraph explaining why other existing models are not applicable to this task.
C. Assess Computational Overhead: Based on my understanding, computational efficiency is a key advantage of MagicImpute. Therefore, it would be better if the authors reported key computational performance metrics, including wall-clock time and peak memory usage, for the compared software.
D. Demonstrate Downstream Utility: The authors have demonstrated the accuracy of MagicImpute, especially relative to other tools. It would now be helpful to add at least a brief discussion (or example) showing that the accuracy gains from MagicImpute translate into gains in downstream biological analyses.
3. Address the Violation of the HMM Independence Assumption:
The manuscript appropriately notes a limitation of the HMM framework-namely, the assumption of conditional independence across markers, which can be violated when reads span multiple polymorphic sites. It would be helpful to modestly expand the Discussion to reflect on the potential impact of this violation in practice and to clarify how far the current strategies (richer error modeling and marker binning) are expected to mitigate it. A brief qualitative comparison of why the error models appear to influence recombination breakpoint estimates differently in simulated versus real datasets would add useful context for readers and practitioners.

Reviewer #2 :

The manuscript presents an extension to a previously published method for genotype imputation in a multiparental population. The new approach contains two main features. First, it partitions founders into blocks. This increases computational efficiency when the number of founders is large and allows for greater missingness in founder genotypes. Second, it uses a beta-binomial model to account for genotype-calling errors more accurately. The authors evaluated the new method with extensive simulation and applied it to three experimental datasets.

The study addresses a crucial topic in genetics with implications for agricultural applications. The proposed framework appears to be a useful addition to existing genetic methods for multiparental populations.

According to the guidelines (https://academic.oup.com/genetics/pages/guidelines-for-reviewers), I have the following comments.

Major points:
1. I was wondering if the authors could clarify whether it would be possible and useful to directly compare the previous method, MagicImpute_mma (Zheng et al., 2018), and the current method, MagicImpute, using datasets to which both methods can be applied. I would like to see such analyses, if this did not require an unreasonable amount of additional work. In particular, it might be informative to compare computational requirements between the previous and current methods.
2. It might not be readily obvious to non-specialists why the current method is suited for connected multiparental populations. A further explanation might be helpful.
3. In line 316, it is stated that "$N_b = 1$ often did not work well for bi-parental populations (results not shown)." It would be ideal to provide the results in the supplementary material, if possible.
4. In lines 322-324, it is stated that "As shown in Figure S2, the
estimation bias, the average difference between estimated and true values, decreased asymptotically into zero with population size and read depth." While this appears true for allelic bias, allelic overdispersion, and sequence-base error, the offspring allelic error appears to approach -0.002, not zero. This issue might need to be clarified.

Minor points:
5. Adding a summary of simulation results to the abstract might be helpful.
6. In the Methods section, it might be useful to briefly describe the process model and offspring imputation for completeness, although these steps have been detailed in previously published manuscripts.
7. It might be useful to provide a DAG of the HMM, particularly for non-specialists.
8. As in Table 1 in Zheng et al. (2018), a table containing all symbols and their descriptions might be useful.
9. In line 124, the subscript $t \in \Omega$ might have to read $f \in \Omega$, if I understand correctly.
10. In line 125, the subscript t might have to read $f \in \Omega$, if I understand correctly.
11. In line 243, the full words for the abbreviation RILs should be added.
12. In lines 297 and 320, "Evaluate" may be changed to "Evaluation" for consistency.
13. According to line 268, the mean of the exponential distribution was set to 5, 10, 20, and 40. However, in Figure 3, results for a depth of 40 are not shown. This point might need to be clarified.
14. Figure S10 does not appear to be referred to in the manuscript.
15. In line 303, "local maximums" should read "local maxima."

Reviewer #3 :

The authors describe a method for genotype imputation in the case of multiple multi-parent populations that have some founder genotype sharing but where genotypes for some founders may be missing. The authors perform simulation studies and test their method in three real data applications. Their method, which fits within the existing software RABBIT and is implemented in the high-performance Julia language, expands upon previous work that performed HMM-based imputation to allow for multiple founders to have high levels of missing data while still being computationally efficient.

This paper seems novel and provides an improvement to the field by providing a framework for imputing genotypes and ancestral haplotype probabilities from sequencing data, accounting for missing founder genotypes, and possible sequencing errors.

Major Comments:

1. MagicImpute has a lot of tuning parameters (5 error parameters, whether or not to bin markers, whether or not to repeat founder analysis, block size, whether or not to refine the map, soft- and hard-threshold cut-offs for deletion of erroneous markers, etc). These all have default values, and I would expect that, given the large number of options, most users would stick with the defaults. If a user were to start with such defaults, how would they know whether additional tuning or non-default settings are needed? Are there diagnostic tests to identify when the inference has gone awry, and how to rescue it? It would be good if the software could provide some sort of quality score or metric to indicate when it is and isnÕt working well on the data itÕs applied to.

2. The methods spends little time on motivationÑonly a few sentencesÑbefore diving into model specifics. It would be helpful to the reader to begin with a motivating example and/or figure showing a hypothetical example of a suitable target population (ie, shared founders and missing genotypes for some).

3. It would be helpful to discuss more where this paper and method fit into the overall RABBIT and Magic* ecosystem. If itÕs possible to compare your results with past versions of MagicImpute (in terms of accuracy and/or computation time) that would be helpful.

4. This method and paper seem geared toward plants, but can it be applied in non-diploid plants or in diploid animals? This would be good to discuss in the introduction and/or discussion, especially because in the original RABBIT paper (PMID: 26048018), the real data that they use includes the Collaborative Cross (CC) and Diversity Outbred (DO) mouse populations.

5. The authors mention R/qtl2 being a multiparent population (MPP) imputation tool that fails here, but one tool that wouldnÕt necessarily fail is HAPPY (PMID: 11050180). HAPPY was used to analyze one of the first plant MPPs (PMID: 11050180) and allows for encoding a kind of missingness in the founder genotype. Specifically, rather than specifying the genotype emitted by each strain, it allows the user to specify the probability each strain emits each possible allele, allowing for missingness by, for example, allowing a strain to emit any allele. At a minimum, it would be good to mention HAPPY in the introduction and/or discussion.

6. It would be helpful to provide a table with a list of symbols and their brief descriptions, preferably in the main paper but otherwise potentially in the supplement or appendix.

7. Neither the main text nor the Figure 1 caption provide specific details on when iterations end and what the convergence criteria is. I was not sure what "the founder analysis is iterated several times until the run with the largest likelihood is close enough to the second best run so far" (lines 109-110) means practically.

8. The authors include an assumption that offspring are independent conditional on the imputed phased founder genotypes, but what if this is not the case, such that individual specific error terms ξ_i are correlated between individuals due to, eg, sequencing batch?

9. Genotype discordance is a primary evaluation metric, and, from my understanding, it comes from masking 10% of the input genotypic data with read depth greater than or equal to 10 and using these as pseudo-true genotypes. I would be interested to know what effect varying which 10% of the input genotypic data was masked and/or using estimated imputation metrics on the purely imputed genotypes would have on these comparisons.

10. You didnÕt discuss your ability to resolve haplotype uncertainty. It has been shown that haplotype uncertainty can derail haplotype effect estimation if that estimation does not model their uncertainty probabilistically (eg, PMID:25236455). When you estimate haplotype effects for the QTL mapping results shown in Figure 5, do you account for haplotype uncertainty? (This can be done using, eg, PMID:25236455, or PMID:33082282).

11. The discrepancies between the estimation of recombination density and average genetic lengths and what has been reported in the literature warrant additional discussion.

Minor Points:

12. There are several typos throughout and sentences with grammatical errors that need to be corrected. A few examples: "disconcordance" should be "discordance", in line 80 "efficiency depends many factors" should be "efficiency depends on many factors", in line 100 "A subdivided population is also termed connected populations" should be "A subdivided population is also termed a connected population", etc.

13. Lines 41-43 about multi-parental populations are missing some citations about the original multi-parental populations, including the CC (PMID: 11050180) and DO mouse panels (PMID: 22345611), HS rat panels (reviewed in PMID: 26048018), etc.

14. Lines 51-53 are missing citations (recent imputation methods in humans reviewed in PMID: 22345611).

15. Figure S10 was never mentioned in the main text.

Associate Editor Comments:

Dear Prof. Li

Thank you for your letter concerning our manuscript entitled "Genotype imputation and error estimation in connected multiparental populations" (GENETICS-2025-308674). We are grateful for the time and efforts that you and the reviewers spent on our manuscript. We have now prepared a revision, closely following comments and concerns given by the reviewers. In particular, we have made the following main changes:

- New benchmarking MagicImpute again the previous methods: Bealge5.5, GBSceanR, and MagicImpute_mma in two simulated populations.
- New benchmarking MagicImpute again GBScleanR in the real apple CP, in addition to the rice F2. Calculate the accuracies based on the same set of markers.
- New sensitivity analyses for the significant level used in the test-based deletion and Tukey's fence in the soft-threshold-based deletion
- Update MagicImpute to calculate and export AR2, a metric for imputation accuracy, as did in Beagle 4.1.

We hope that you find the revision suitable for publication in Genetics.

Sincerely,

Chaozhi Zheng

On behalf of Eligio Bossolini, Nataša Formanová, Antje Rohde, Martin P. Boer, and Fred A. van Eeuwijk

Biometris

Wageningen UR, The Netherlands

chaozhi.zheng@wur.nl

Responses to the reviewers

We thank the reviewers for their constructive comments. Below, our point-by point responses are shown in blue. Line numbers in our responses refer to the clean version of the manuscript (rather than the marked version).

Reviewer #1

1. Threshold and marker-deletion sensitivity:
The current settings for soft/hard deletion thresholds and the default Tukey fence of 2 appear empirical while the authors explained that 1.5 tends to over-delete whereas 3 tends to under-delete. Besides, the iterative marker-deletion step based on the Vuong test (alpha = 0.01) is introduced without a systematic calibration. Therefore, I recommend adding a concise supplementary sensitivity analysis by performing a small grid scan over the combinations of hard thresholds, soft thresholds/Tukey fence values, and on/off use of the Vuong test. This addition would substantially improve reproducibility and strengthen the empirical justification of the pipeline's curation settings.

Thank you for this suggestion. We have added sensitivity analyses for Tukey fences 1.5, 2, and 3; see the new Figures S10-S11. We have also added sensitivity analyses for the significance levels 0.001, 0.01, and 0.05; see new Figures S12-S13. See also the new lines 347-369 for the results on sensitivity analyses. We have chosen only three typical optional values because of computational time, and it seems they provide sufficient guides.

2. Insufficient and Unbalanced Benchmarking:
The current benchmarking of MagicImpute against existing methods, particularly GBScleanR, seems insufficient and unbalanced, preventing a clear assessment of the tool's practical advantages. Specifically, MagicImpute is shown to achieve lower genotypic disconcordance, but only after deleting approximately 45% of markers, whereas GBScleanR retained all markers. This constitutes an unequal comparison, essentially contrasting "high accuracy via aggressive filtering" with "high marker retention." To enhance the benchmarking, I have the following suggestions.
A. Establish Fair Baseline Comparisons: It would be better if the authors could discuss more fully why their comparison is practically reasonable, useful, and informative. Alternatively, the authors could apply a uniform post-imputation quality filter to the GBScleanR output (e.g., matching the final marker density of MagicImpute) before comparing accuracy on the commonly retained markers. Either approach would be acceptable to me.

We agree with you on the limitation of the previous benchmark, and thank you for the suggestion. We have performed two additional benchmark studies:

1. Benchmark in two simulated populations, where true values are known and marker deletion was disabled for MagicImpute. See Figures 2 and S1-S2, and see new lines 347-369 in the Results.

2. Benchmark in the apple CP beside the rice F2. We have calculated the accuracies of GBScleanR in two ways: based on all the markers, and based on the same set of markers retained by MagicImpute.

B. Expand Benchmarking Scope: Are there other applicable baseline tools beyond GBScleanR? If so, would the authors add them-or at least one previously strong model-to the comparison? If not, please add a paragraph explaining why other existing models are not applicable to this task.

We have performed additional benchmark in two simulated populations, where MagicImpute was compared with GBScleanR, Beagle, and MagicImpute_mma. See Figures 2 and S1-S2.

C. Assess Computational Overhead: Based on my understanding, computational efficiency is a key advantage of MagicImpute. Therefore, it would be better if the authors reported key computational performance metrics, including wall-clock time and peak memory usage, for the compared software.

We have reported computation time in the simulation benchmark studies. We have simulated two benchmarking population: a CP with 2 parents, and a connected multiparental populations with 5 parents, aiming to illustrate the different dependence of computational time on the number of founders. See Figures 2 and S1-S2.

We did not report the peak memory usage, because there is no way to record it in the Julia language, as far as I know. In addition, our rough estimation of memory usage is relatively small.

D. Demonstrate Downstream Utility: The authors have demonstrated the accuracy of MagicImpute, especially relative to other tools. It would now be helpful to add at least a brief discussion (or example) showing that the accuracy gains from MagicImpute translate into gains in downstream biological analyses.

We have followed the suggestions A, B and C, but not D, partly because the demonstration depends on downstream analyses, and partly because we have illustrated one application in the real Sorghum population.

3. Address the Violation of the HMM Independence Assumption:
The manuscript appropriately notes a limitation of the HMM framework-namely, the assumption of conditional independence across markers, which can be violated when reads span multiple polymorphic sites. It would be helpful to modestly expand the Discussion to reflect on the potential impact of this violation in practice and to clarify how far the current strategies (richer error modeling and marker binning) are expected to mitigate it. A brief qualitative comparison of why the error models appear to influence recombination breakpoint

estimates differently in simulated versus real datasets would add useful context for readers and practitioners.

We have expanded the discussion with one additional Figure S19, where we have kept only the first markers in each sequence stretch of length 75bp prior to imputation. The results have shown that this thinning did greatly reduce the number of breakpoints, although it may confound with the effects of the number of markers. The effects of current strategies have been shown in Figures7 & S18. The different impact of error models on #breakpoints is probably because of simulated data following the conditional independence assumption.

Reviewer #2

Major points

1. I was wondering if the authors could clarify whether it would be possible and useful to directly compare the previous method, MagicImpute_mma (Zheng et al., 2018), and the current method, MagicImpute, using datasets to which both methods can be applied. I would like to see such analyses, if this did not require an unreasonable amount of additional work. In particular, it might be informative to compare computational requirements between the previous and current methods.

Thank you for this suggestion. We have added benchmarking in simulated populations, where MagicImpute was compared with the previous methods including MagicImpute_mma. See Figures 2 and S1-S2, where computational time was also reported.

2. It might not be readily obvious to non-specialists why the current method is suited for connected multiparental populations. A further explanation might be helpful.

We have revised in new lines 82-84 in the Introduction: *Specifically, we partition founders into blocks and impute founder blocks alternately, and thus computational time depends mainly on the founder block sizes rather than the total number of founders.*

3. In line 316, it is stated that "$N_b = 1$ often did not work well for bi-parental populations (results not shown)." It would be ideal to provide the results in the supplementary material, if possible.

We have added Figure S4 to show the results for Nb=1.

4. In lines 322-324, it is stated that "As shown in Figure S2, the estimation bias, the average difference between estimated and true values, decreased asymptotically into zero with population size and read depth." While this appears true for allelic bias, allelic overdispersion, and sequence-base error, the offspring allelic error appears to approach -0.002, not zero. This issue might need to be clarified.

The very small negative value is due to marker deletion. We have added in new lines 397-399: *The negative estimation biases were mainly because of markers with large error rates being deleted, whereas the positive estimation biases were mainly because of the large prior means.*

Minor points

5. Adding a summary of simulation results to the abstract might be helpful.

We have revised accordingly. See new lines 37-38.

6. In the Methods section, it might be useful to briefly describe the process model and offspring imputation for completeness, although these steps have been detailed in previously published manuscripts.

We have briefly described the process model in new lines 129-131. We have added a subsection on offspring analysis in new liens 259-274.

7. It might be useful to provide a DAG of the HMM, particularly for non-specialists.

We did not provide a DAG of the HMM, partly because the DAG is too complicated to account for the population structure of connected population, and partly because the DAG would look like the previous one except for the population structure.

8. As in Table 1 in Zheng et al. (2018), a table containing all symbols and their descriptions might be useful.

We have added Table S1 accordingly.

9. In line 124, the subscript $t \in \Omega$ might have to read $f \in \Omega$, if I understand correctly.

We have revised accordingly.

10. In line 125, the subscript t might have to read $f \in \Omega$, if I understand correctly.

We have revised accordingly.

11. In line 243, the full words for the abbreviation RILs should be added.

We have revised accordingly.

12. In lines 297 and 320, "Evaluate" may be changed to "Evaluation" for consistency.

We have revised accordingly.

13. According to line 268, the mean of the exponential distribution was set to 5, 10, 20, and 40. However, in Figure 3, results for a depth of 40 are not shown. This point might need to be clarified.

We have explained the legend of new Figure 4 and S5: For the sake of visualization, results for depth 40 are not shown.

14. Figure S10 does not appear to be referred to in the manuscript.

We have cited it (new Figure S17) in new lines 470 & 475.

15. In line 303, "local maximums" should read "local maxima."

We have revised accordingly.

Reviewer #3

Major Comments

1. MagicImpute has a lot of tuning parameters (5 error parameters, whether or not to bin markers, whether or not to repeat founder analysis, block size, whether or not to refine the map, soft- and hard-threshold cut-offs for deletion of erroneous markers, etc). These all have default values, and I would expect that, given the large number of options, most users would stick with the defaults. If a user were to start with such defaults, how would they know whether additional tuning or non-default settings are needed? Are there diagnostic tests to identify when the inference has gone awry, and how to rescue it? It would be good if the software could provide some sort of quality score or metric to indicate when it is and isnÕt working well on the data itÕs applied to.

You have raised an important point. We have added sensitivity analyses for two tunning parameters: the significance level for the test-base deletion and Tukey's fence for the soft-threshold-based deletion, aiming to provide some guides for users. We have updated MagicImpute to export a metric for imputation accuracy at each marker, AR2 (allelic squared correlation), as Beagle 4.1 did. We have added one paragraph in the Discussion on tunning parameters; see new lines 535-544.

2. The methods spends little time on motivationÑonly a few sentencesÑbefore diving into model specifics. It would be helpful to the reader to begin with a motivating example and/or figure showing a hypothetical example of a suitable target population (ie, shared founders and missing genotypes for some).

We have added two example target populations in Figure 1A-B: a CP and a connected multiparental population. The second target population has three out of five founders with all genotypes missing.

3. It would be helpful to discuss more where this paper and method fit into the overall RABBIT and Magic* ecosystem. If itÕs possible to compare your results with past versions of MagicImpute (in terms of accuracy and/or computation time) that would be helpful.

We have compared MagicImpute with MagicImpute_mma and others in two simulated populations in terms of accuracy and computational time. See Figures 1 and S1-S2, and the first section "Comparison with previous methods" in the Results.

4. This method and paper seem geared toward plants, but can it be applied in non-diploid plants or in diploid animals? This would be good to discuss in the introduction and/or discussion, especially because in the original RABBIT paper (PMID: 26048018), the real data that they use includes the Collaborative Cross (CC) and Diversity Outbred (DO) mouse populations.

We have added one paragraph in the Discussion on the animal populations; see new lines 552-555. In short, the current MagicImpute works for autosomes in animal populations but not for sex chromosomes.

5. The authors mention R/qtl2 being a multiparent population (MPP) imputation tool that fails here, but one tool that wouldnÕt necessarily fail is HAPPY (PMID: 11050180). HAPPY was used to analyze one of the first plant MPPs (PMID: 11050180) and allows for encoding a kind of missingness in the founder genotype. Specifically, rather than specifying the genotype emitted by each strain, it allows the user to specify the probability each strain emits each possible allele, allowing for missingness by, for example, allowing a strain to emit any allele. At a minimum, it would be good to mention HAPPY in the introduction and/or discussion.

Yes, we have cited R/HAPPY in the Introduction; see new line 53. We did not cite it in the previous manuscript, because R/HAPPY is for haplotype reconstruction but not for genotype imputation, whereas R/qtl2 works for both.

6. It would be helpful to provide a table with a list of symbols and their brief descriptions, preferably in the main paper but otherwise potentially in the supplement or appendix.

We have added Table S1 accordingly.

7. Neither the main text nor the Figure 1 caption provide specific details on when iterations end and what the convergence criteria is. I was not sure what "the founder analysis is iterated

several times until the run with the largest likelihood is close enough to the second best run so far" (lines 109-110) means practically.

We have revised the text in new lines 118-126:

To increase the robustness of founder genotype estimation, the founder analysis is repeated when it is challenging to find the global optimal imputation. By default, founder analysis is repeated from three to six times if the following conditions are satisfied: founders are partitioned into at least two blocks, and either founders are outbred or there is at least one founder with a large missing fraction (> 0.6). During iterative repeating, we keep track of the repeat with the largest posterior probability and stop repeating if there are at least two other repeats satisfying either the log posterior probability difference with the largest one is less than $ln(10)$ or the founder genotype difference is less than 0.1%.

8. The authors include an assumption that offspring are independent conditional on the imputed phased founder genotypes, but what if this is not the case, such that individual specific error terms ξ_i are correlated between individuals due to, eg, sequencing batch?

Thank you for pointing this out. The assumption can be regarded as a prior distribution for xi_i. The posterior estimation of xi_i should reflect that offspring from the same batch have more similar error rates than those from different batches. It would be interesting to test this aspect in the future studies.

9. Genotype discordance is a primary evaluation metric, and, from my understanding, it comes from masking 10% of the input genotypic data with read depth greater than or equal to 10 and using these as pseudo-true genotypes. I would be interested to know what effect varying which 10% of the input genotypic data was masked and/or using estimated imputation metrics on the purely imputed genotypes would have on these comparisons.

We have revised the text to indicate the 10% masked genotype were chosen randomly.

We did not study the effect of masking patterns because the true values are not known and the effect might confound with the errors in masked genotypes. Instead, we have studied the effect of missing patterns in the simulated benchmarking populations (Figures 2 and S1-S2). There were two extreme missing patterns in simulated founders: half of the founders with all genotypes missing, and the others with genotypes missing at random with probability 0.05.

10. You didnÕt discuss your ability to resolve haplotype uncertainty. It has been shown that haplotype uncertainty can derail haplotype effect estimation if that estimation does not model their uncertainty probabilistically (eg, PMID:25236455). When you estimate haplotype effects

for the QTL mapping results shown in Figure 5, do you account for haplotype uncertainty? (This can be done using, eg, PMID:25236455, or PMID:33082282).

We have incorporated the haplotype uncertainty in the QTL mapping using statgenMPP (new Figure 6), where posterior origin-genotype probabilities were used as genetic predictors rather than the most probable origin-genotypes.

11. The discrepancies between the estimation of recombination density and average genetic lengths and what has been reported in the literature warrant additional discussion.

We have added the comparisons of our estimations with the previous reported values. See new lines 466-474.

Minor Points

12. There are several typos throughout and sentences with grammatical errors that need to be corrected. A few examples: "disconcordance" should be "discordance", in line 80 "efficiency depends many factors" should be "efficiency depends on many factors", in line 100 "A subdivided population is also termed connected populations" should be "A subdivided population is also termed a connected population", etc.

We have corrected them accordingly. We have reviewed the manuscript for grammatical accuracy.

13. Lines 41-43 about multi-parental populations are missing some citations about the original multi-parental populations, including the CC (PMID: 11050180) and DO mouse panels (PMID: 22345611), HS rat panels (reviewed in PMID: 26048018), etc.

We have cited the CC in new line 554 as an example animal population.

14. Lines 51-53 are missing citations (recent imputation methods in humans reviewed in PMID: 22345611).

We have cited a recent review in new line 59. The cited review seems more relevant than the suggested one.

15. Figure S10 was never mentioned in the main text.

We have cited it (new Figure S17) in new lines 470 & 475.

April 14, 2026

GENETICS-2026-309248
Genotype imputation and error estimation in connected multiparental populations

Dear Dr. Zheng:

Three experts in the field have reviewed your manuscript, and I have read it as well. All agree that the manuscript has improved substantially. While your manuscript is not currently acceptable for publication in GENETICS, we would welcome a revised manuscript. Two reviewers have comments and concerns to be addressed in a revised manuscript. You can read their reviews at the end of this email. Please address the remaining comments and we look forward to receiving your revised manuscript. Please let the editorial office know approximately how long you expect to need for revisions.

Upon resubmission, please include:
1. A clean version of your manuscript;
2. A marked version of your manuscript in which you highlight significant revisions carried out in response to the major points raised by the editor/reviewers (track changes is acceptable if preferred);
3. A detailed response to the editor's/reviewers' feedback and to the concerns listed above. Please reference line numbers in this response to aid the editor and reviewers.

Your paper will likely be sent back out for review.

Additionally, please ensure that your resubmission is formatted for GENETICS
https://academic.oup.com/genetics/pages/general-instructions

Follow this link to submit the revised manuscript: Link Not Available

Sincerely,

Yun Li
Associate Editor
GENETICS

Approved by:
Hongyu Zhao
Senior Editor
GENETICS

Reviewer #1 :

Thank you for the careful revision and the detailed point-by-point response. The manuscript has been substantially strengthened. In particular, the added benchmarking against previous methods, the fairer comparisons using matched retained-marker sets for GBScleanR, the new sensitivity analyses for Tukey's fence and the Vuong-test significance level, and the expanded discussion of the HMM conditional-independence assumption have adequately addressed my main concerns. I have no further substantive comments.

Reviewer #2 :

The authors have addressed my major comments. I now only have the following minor comments.

Minor points:
1. In lines 94-102, it might be useful to refer to the newly added benchmarking analyses.
2. In lines 129-133, an alternative expression, such as the following, might be clearer: "consisting of three components: initial, transition, and emission probabilities. The initial and transition probabilities describe the hidden origin process along chromosomes and are calculated by a recursive algorithm using pedigree information (ZHENG et al. 2014, 2018b). The emission probability describes the genotypic data model (ZHENG et al. 2015, 2018a)."
3. In line 283, "recombination inbred lines" may have to read "recombinant inbred lines."
4. In the "Comparison with previous methods" section, it might be useful to distinguish further between what was already known

and what the current benchmarking analysis revealed. For example, the former and latter can be described in the present and past tenses, respectively.

Reviewer #4 :

The authors have improved the quality of the manuscript by addressing reviewers' comments. In particular, the authors added sensitivity analyses, expanded benchmarking analyses to more simulation settings, and provided additional discussion on model misspecification. Overall, MagicImpute could be a potentially useful tool for MPPs genotype imputation, and it enabled flexible modeling of complicated error structures. My main concerns are about the practical utility of MagicImpute regarding its computational efficiency, parameter tuning, and biological implications. Please see below for details:

Major:
1. The author claimed that one major advantage of MagicImpute is its scalability. However, for the three scenarios shown in figure 2, MagicImpute is slower than Beagle in the first panel, slower than MagicImpute_mma in the second panel when population size is smaller than 50 or equal to 200, and slower than MagicImpute_mma in the third panel. Also, it is unclear whether the claimed computation efficiency stemmed from algorithmic innovation or merely implementing by a more efficient language. Moreover, in line 315, the author mentioned that MagicImpute_mma did not use multiple threading while MagicImpute used five threads. Thus, an equal wall time for MagicImpute_mma and MagicImpute actually means MagicImpute costs five times more CPU computation than MagicImpute_mma. Overall, it is hard to justify MagicImpute is more computationally efficient than other methods based on the current manuscript.
2. In the revised manuscript, the authors provided additional sensitivity analysis in Figure S10-13 regarding parameter choice. The authors also added AR2 as a performance metric for parameter selection. While these additions are useful and should be commended, there is still no systematic user guidance on how each parameter should be changed if the imputation results are not desirable. The author should provide a more detailed model diagnostics guidance. Moreover, the authors should also systematically calibrate all tunable parameters in the model instead of selecting a subset to perform sensitivity analysis.
3. The authors did not address the biological utility of MagicImpute sufficiently. Reviewer 1 explicitly asked for further biological validations. The author's response was simply that they followed suggestions A-C "but not D", because it depends on downstream analysis and the because they have illustrated one application example in Sorghum population. The author should at least explain what new biological findings are enabled by MagicImpute but not by previous methods. In short, without a concrete downstream demonstration, it remains unclear whether the reported gains in discordance or breakpoint behavior meaningfully improve biological conclusions.
Minor:
1. The author claimed in the rebuttal letter that they are unaware of any ways to measure memory usage for Julia. However, a simple command like "/usr/bin/time -v julia hello_world.jl" will print peak memory usage. I suggest the author to add memory usage and CPU time for complete computational performance benchmark.
2. A concise summary table listing the main tunable parameters, their defaults, and their intended effects would improve readability and usability.
3. The manuscript would benefit from a clearer separation between improvements due to better modeling and improvements due to marker deletion. At present, these two effects are sometimes discussed together, which makes it harder to interpret where the gains are coming from.
4. Currently the figure legend position is not consistent. For example, figure 2 has all legends presented in only the middle panels, but figure 3 and figure 4 have legend only at the lower left panel. I suggest the authors to add legend in each panel or put an overall legend outside for better figure readability.

Associate Editor Comments:

Dear Prof. Li

Thank you for your letter concerning our manuscript entitled "Genotype imputation and error estimation in connected multiparental populations" (GENETICS-2026-309248). We are grateful for the time and efforts that you and the reviewers spent on our manuscript. We have now prepared another revision, closely following comments and concerns given by the reviewers.

We hope that you find the revision suitable for publication in Genetics.

Sincerely,

Chaozhi Zheng

On behalf of Eligio Bossolini, Nataša Formanová, Antje Rohde, Martin P. Boer, and Fred A. van Eeuwijk

Biometris

Wageningen UR, The Netherlands

chaozhi.zheng@wur.nl

Responses to the reviewers

We thank the reviewers for their constructive comments. Below, our point-by point responses are shown in blue. Line numbers in our responses refer to the clean version of the manuscript (rather than the marked version).

Reviewer #2 :

Minor:

1. In lines 94-102, it might be useful to refer to the newly added benchmarking analyses.

We have revised in lines 94-97: "Then we evaluate MagicImpute through extensive simulation studies, including benchmarking MagicImpute against previous software Beagle 5.5 \citep{Browning2018}, MagicImpute$_$mma \citep{Zheng2018b}, and GBScleanR \citep{Furuta2023}, and testing the imputation of many ungenotyped founders and the estimation of various error rates".

2. In lines 129-133, an alternative expression, such as the following, might be clearer: "consisting of three components: initial, transition, and emission probabilities. The initial and transition probabilities describe the hidden origin process along chromosomes and are calculated by a recursive algorithm using pedigree information (ZHENG et al. 2014, 2018b). The emission probability describes the genotypic data model (ZHENG et al. 2015, 2018a)."

We have revised it accordingly.

3. In line 283, "recombination inbred lines" may have to read "recombinant inbred lines."

We have corrected it accordingly.

4. In the "Comparison with previous methods" section, it might be useful to distinguish further between what was already known and what the current benchmarking analysis revealed. For example, the former and latter can be described in the present and past tenses, respectively.

We have revised the section accordingly.

Reviewer #4 :

Overall, MagicImpute could be a potentially useful tool for MPPs genotype imputation, and it enabled flexible modeling of complicated error structures. My main concerns are about the practical utility of MagicImpute regarding its computational efficiency, parameter tuning, and biological implications. Please see below for details:

Major:

1. The author claimed that one major advantage of MagicImpute is its scalability. However, for the three scenarios shown in figure 2, MagicImpute is slower than Beagle in the first panel, slower than MagicImpute_mma in the second panel when population size is smaller than 50 or equal to 200, and slower than MagicImpute_mma in the third panel. Also, it is unclear whether the claimed computation efficiency stemmed from algorithmic innovation or merely implementing by a more efficient language. Moreover, in line 315, the author mentioned that MagicImpute_mma did not use multiple threading while MagicImpute used five threads. Thus, an equal wall time for MagicImpute_mma and MagicImpute actually means MagicImpute costs five times more CPU computation than MagicImpute_mma. Overall, it is hard to justify MagicImpute is more computationally efficient than other methods based on the current manuscript.

We have added a new benchmarking study as shown in Figure S3, where we compared MagicImpute with MagicImpute_mma and GBScleanR for three populations with GBS data and increasing numbers of founders: 2, 5, and 7. Previously we failed for the simulated

populations with 7 founders and 5 linkage groups. Thus, we analysed only one linkage group in current version. See lines 364-369:

"In comparison with GBScleanR and MagicImpute$_$mma, the computation time and peak memory usage of MagicImpute increased much more slowly with the number of founders (Figure S3). For the benchmarking population with seven founders and one linkage group, GBScleanR took 62 hours, much longer than the 0.29 hour of MagicImpute, and MagicImpute$_$mma used 18 Gibibyte (GiB) memory, much more than the 3.5 GiB of MagicImpute (Figure S3)."

We have found out that Mathematica adopted multithreading automatically for MagicImpute_mma. We have re-run MagciImpute_mma for all simulations with explicit 5 threads for consistency. The new benmarking study in Figure S3 is conservative for MagicImpute, since it was performed without parallel processing in the case of one linkage group, whereas MagicImpute_mma and GBScleanR were performed with five parallel threads.

2. In the revised manuscript, the authors provided additional sensitivity analysis in Figure S10-13 regarding parameter choice. The authors also added AR2 as a performance metric for parameter selection. While these additions are useful and should be commended, there is still no systematic user guidance on how each parameter should be changed if the imputation results are not desirable. The author should provide a more detailed model diagnostics guidance. Moreover, the authors should also systematically calibrate all tunable parameters in the model instead of selecting a subset to perform sensitivity analysis.

We have added Table S2 for the main options of MagicImpute and added a section on "Guidance on MagicImpute options" in File S1 for calibrating the main options.

3. The authors did not address the biological utility of MagicImpute sufficiently. Reviewer 1 explicitly asked for further biological validations. The author's response was simply that they followed suggestions A-C "but not D", because it depends on downstream analysis and the because they have illustrated one application example in Sorghum population. The author should at least explain what new biological findings are enabled by MagicImpute but not by previous methods. In short, without a concrete downstream demonstration, it remains unclear whether the reported gains in discordance or breakpoint behavior meaningfully improve biological conclusions.

We have added in Discussion one paragraph on the capabilities of MagicImpute and the incapabilities of the previous methods (line500-508):

"The benchmarking studies have shown that MagicImpute was usually more accurate than Beagle 5.5 \citep{Browning2018}, MagicImpute$_$mma \citep{Zheng2018b}, and

GBScleanR \citep{Furuta2023}, in terms of genotypic discordance and parental phasing. We chose Beagle 5.5 as a baseline for comparisons, but it is not the main competitor of MagicImpute. Beagle 5.5 shows an incapability to impute ungenotyped founders and to account for sequencing errors, although it is computationally efficient. In comparison with MagicImpute$_$mma and GBScleanR, MagicImpute scaled much better with the number of founders, allowing for integrating many bi- or multi-parental populations. It is worth noting that accurate parental imputation and phasing are required for downstream haplotype reconstruction and QTL mapping."

Minor:

1. The author claimed in the rebuttal letter that they are unaware of any ways to measure memory usage for Julia. However, a simple command like "/usr/bin/time -v julia hello_world.jl" will print peak memory usage. I suggest the author to add memory usage and CPU time for complete computational performance benchmark.

Thanks for the suggestion. We have presented the memory usages in Figure S3.

2. A concise summary table listing the main tunable parameters, their defaults, and their intended effects would improve readability and usability.

We have added Table S2 accordingly.

3. The manuscript would benefit from a clearer separation between improvements due to better modeling and improvements due to marker deletion. At present, these two effects are sometimes discussed together, which makes it harder to interpret where the gains are coming from.

Marker deletion was disabled for MagicImpute in all simulation benchmarking studies. In addition for the real rice and apple, we have added Figure S20, similar to Figure 7 except that marker deletion was disabled for MagicImpute.

4. Currently the figure legend position is not consistent. For example, figure 2 has all legends presented in only the middle panels, but figure 3 and figure 4 have legend only at the lower left panel. I suggest the authors to add legend in each panel or put an overall legend outside for better figure readability.

We have consistently put legends on the top panels in Figures 2-5.

June 27, 2026
RE: GENETICS-2026-309607

Dear Dr. Zheng:

I am pleased to accept your manuscript titled "Genotype imputation and error estimation in connected multiparental populations" for publication in GENETICS, pending minor revision.

Please submit your revision along with a brief description of how you modified the manuscript in response to the reviewers' concerns and suggestions.

I expect you should be able to submit a revised manuscript within 30 days. A suitably revised manuscript will be acceptable for publication; I don't expect to send it out for review.

Prior to uploading your revised manuscript please format it according to GENETICS style and ensure you have all required elements. Author Guidelines are at https://academic.oup.com/genetics/pages/author-guidelines#section-12. These guidelines have been updated with new requirements and your careful attention is required to avoid delays at the source file stage post-acceptance.

When revising the ms., please make an effort to shorten it, because that almost always improves a manuscript. We urge authors to heed the advice of Strunk and White: "omit needless words"1. Follow this link to submit the revised manuscript: Link Not Available

Thank you for submitting this story to Genetics.

Sincerely,

Yun Li
Associate Editor
GENETICS

Approved by:
Hongyu Zhao
Senior Editor
GENETICS

Reviewer comments:
Reviewer #2 :

The authors have addressed all my comments. I have a few minor comments that can be addressed optionally.

1. In Figures S3E and S3F, the numbers on the top of the bars can be moved upward to avoid overlap, which may improve readability. Alternatively, they can be omitted.

2. In Figure 7, S19, S20, and S21, the label "GBScleanR" can be moved downward to avoid the overlap with the x-axis, which may improve readability.

3. On line 473 in the marked-up document, "anallysis" should read "analysis."

Reviewer #4 :

The authors have addressed all my comments from the previous round, and I have no further comments.

Associate Editor comments:

Dear Prof. Li

Thank you for your letter concerning our manuscript entitled "Genotype imputation and error estimation in connected multiparental populations" (GENETICS-2026-309607). We are grateful for the time and efforts that you and the reviewers spent on our manuscript. We have now prepared another revision according to the comments of Reviewer 2.

We hope that you find the revision suitable for publication in Genetics.

Sincerely,

Chaozhi Zheng

On behalf of Eligio Bossolini, Nataša Formanová, Antje Rohde, Martin P. Boer, and Fred A. van Eeuwijk

Biometris

Wageningen UR, The Netherlands

chaozhi.zheng@wur.nl

Responses to the reviewers

We thank the reviewers for their constructive comments. Below, our point-by point responses are shown in blue.

Reviewer #2 :

1. In Figures S3E and S3F, the numbers on the top of the bars can be moved upward to avoid overlap, which may improve readability. Alternatively, they can be omitted.

We have omitted the number annotations.

2. In Figure 7, S19, S20, and S21, the label "GBScleanR" can be moved downward to avoid the overlap with the x-axis, which may improve readability.

We have moved the label downward in the four figures.

3. On line 473 in the marked-up document, "anallysis" should read "analysis."

We have corrected it in new line 449.

July 31, 2026
RE: GENETICS-2026-309607R1

Dr. Chaozhi Zheng
Wageningen University & Research
Mathematical & Statistical Methods - Biometris
P.O. Box 16, 6700 AA Wageningen
Wageningen 6708 PB
Netherlands

Dear Dr. Zheng:

Congratulations, your manuscript titled "Genotype imputation and error estimation in connected multiparental populations" is accepted for publication in GENETICS! Many thanks for submitting your research to the journal.

To Proceed to Publication:
1. Format your article according to GENETICS style: https://academic.oup.com/genetics/pages/author-guidelines

2. Ensure that you comply with data and community resource citation guidelines:
https://academic.oup.com/genetics/pages/author-guidelines#section-5-9-2

3. Upload your final files at https://genetics.msubmit.net

4. Add oupsupport@scipris.com and genetics.oup@novatechset.com (or the domains @scipris.com and @novatechset.com) to your email program's "safe senders" list. You will be contacted by both at various points during the production process.

Notes:
- Your currently-accepted manuscript (unedited, as submitted, reviewed, and accepted) will be published at GENETICS and deposited into PubMed as an Advance Access article. Notify sourcefiles@thegsajournals.org before signing your license if you do not wish to publish your article via Advance Access.

- We invite you to submit an original color figure related to your paper for consideration as cover art. Please note that submissions created with AI image generation tools are not eligible. Please email your submission to the editorial office or upload it with your final files. You can submit a small-sized image for evaluation, and if selected, the final image must be a TIFF file 2513px wide by 3263px high (8.375 by 10.875 inches; resolution of 600ppi). Please avoid graphs and small type.

- After files are sent to Oxford University Press we use SciPris to manage article licensing and payment. If you do not have a SciPris account, you will receive an email from no-reply@scipris.com to sign up to use Oxford University Press' author portal. After logging in, follow the online instructions to sign your license and arrange any payment due.

If you have any questions or encounter any problems while uploading your accepted manuscript files, please email the editorial office at sourcefiles@thegsajournals.org.

Sincerely,

Yun Li
Associate Editor
GENETICS

Approved by:
Hongyu Zhao
Senior Editor
GENETICS

Review comments (if applicable):
