# Supplementary material for: Genotype imputation and error estimation in connected multiparental populations
Source: Genetics. 2026 Aug 17;234(2):iyag217. doi: 10.1093/genetics/iyag217 (PMC13643761; doi:10.1093/genetics/iyag217)
Supplement: Supplementary file 2 [file iyag217_supplementary_data.pdf]

Genotype imputation and error estimation in connected multiparental populations — Supplemental Material

Chaozhi Zheng[1,*], Eligio Bossolini[2], Nataša Formanová[2], Antje Rohde[2,3], Martin P. Boer[1], Fred A. van Eeuwijk[1]

[1] Mathematical & Statistical Methods - Biometris, Wageningen University & Research, 6708PB Wageningen, The Netherlands

[2] BASF Belgium Coordination Center CommV, Technologiepark-Zwijnaarde 101, 9052 Ghent, Belgium

[3] Current address: KWS Saat SE, 37574 Einbeck, Germany

Details on data analysis

We performed data analyses using RABBIT v1.3.7. See the online manual `https://github.com/Biometris/RABBIT` for all RABBIT functions. We used GBScleanR v2.5.5 for analyzing the rice F2. See Table S2 for the options of MagicImpute.

Rice F2

The genotypic data file "`gbs_nbolf2.vcf.gz`" was downloaded online `https://github.com/tomoyukif/GBSR_SupFiles`. We analyzed the data by the following steps.

We first filtered the data by function `vcffilter` in the RABBIT

```
vcffilter(genofile;
    delsamples = ["03_F1"],
    maxmiss = 0.99,
    minmaf = 0.05,
    deldupe = true,
)
```

where option `delsamples` deletes the sample "`03_F1`", `maxmiss` filters for markers with genotype missing fraction <= 0.99, `minmaf` filters for markers with minor allele frequency >= 0.05, and `deldupe` deletes successive markers having exactly the same genotypic data. After filtering, the number of markers was reduced from 20,224 to 11,398, and the filtered data was saved in `genofile2`.

We then filtered the new `genofile2` by function `magicfilter` in the RABBIT

```
magicfilter(genofile2,designcode;
    isfounderinbred = true,
    minmaf = 0.1,
    missfilter = (f,o) -> o < 0.95
)
```

where `designcode` is given by "`01_NB/02_OL=>1`", denoting the two parents "`01_NB`" and "`02_OL`" were first crossed and the resulting F1 was then self-fertilized for one generation. Here `isfounderinbred` specifies whether parents are inbred, `minmaf` filters for markers with

minor allele frequency $>= 0.1$, and `missfilter` filters for markers with genotype missing fraction in offspring < 0.95 regardless of missing in parents. The number of markers was further reduced to 8372, and a new `genofile3` and a pedigree file `pedfile` were output.

To calculate imputation accuracy, we masked the filtered data by function `magicmask` in the RABBIT

```
magicmask(genofile3,pedfile;
    isfounderinbred = true,
    minread=10
)
```

where `minread` denotes masked read depths must be no less than 10. By default, the read depths at each marker for each individual (founder of offspring) were masked with probability 0.1 if they met the `minread` requirement. The read depths were converted into genotypes with threshold 0.9 and used as ground truths for calculating genotypic discordances. In the genotype calling, the default sequence error rates were used: no allelic bias, no overdispersion, and sequence base error is set to 0.001. A new `genofile4` was output.

Next, genotype imputation was performed by function `magicimpute` in the RABBIT

```
magicimpute(genofile4,pedfile;
    isfounderinbred = true,
    isphysmap = true,
    recomrate = 4,
    likeparam,
    threshlikeparam,
    softlikeparam,
    tukeyfence = 2,
    iscorrectfounder = true,
    isspacemarker = true,
    skeletonsize = 100,
)
```

where `isphysmap` and `recomrate` denotes transforming the input physical map into a genetic map with recombination rate 4 cM/Mbp, `iscorrectfounder` denotes whether to perform founder error correction, `isspacemarker` denotes whether to infer inter-marker distances, `tukeyfence` denotes Tukey's fence 2, and `skeletonsize` denotes 100 skeleton markers being used for re-scaling estimated inter-marker distances. A new `genofile5` was output.

We set `likeparam = LikeParam(peroffspringerror=0.005, offspringerror = 0.005, allelicbias = 0.5, allelicoverdispersion = 0, baseerror = 0.001)` for null model $M0$. The keywords `peroffspringerror`, `offspringerror`, `allelicbias`, `allelicoverdispersion`, and `baseerror` correspond to the symbols ξ_i, ϵ_t, μ_t, ϕ_t, and η_t in the body text, respectively. Setting the value of an error parameter to `nothing` will infer the error parameter. For example for error model $M3$, `likeparam = LikeParam(peroffspringerror=0.005, offspringerror=nothing, allelicbias = nothing, allelicoverdispersion = nothing, baseerror = 0.001)`.

The option `threshlikeparam` specifices hard thresholds. By default, `threshlikeparam = ThreshLikeParam(peroffspringerror = 0.25, offspringerror = 0.25, allelicbias = 0.9, allelicoverdispersion = 1.0, baseerror = 0.05)`. The `softthreshlikeparam` specifices soft thresholds. By default, `softthreshlikeparam = SoftThreshLikeParam(peroffspringer`

= 0.025, offspringerror = 0.025, allelicbias = 0.67, allelicoverdispersion = 0.01, baseerror = 0.01).

Finally, we run function magicreconstruct in the RABBIT

```
magicreconstruct(genofile5,pedfile;
    isfounderinbred = true,
    hmmalg = "viterbi",
)
```

where hmmalg denotes using the Viterbi algorithm for haplotype reconstruction. The number of recombination breakpoints was calculated by the number of change-points in the inferred Viterbi path of ancestral origins for each offspring in each linkage group.

The genofile4 output by magicmask was loaded by GBScleanR to produce object gdata. It was first filtered by

```
gdata <- setCallFilter(gdata,
    ref_qtile = c(0, read_quantile),
    alt_qtile = c(0, read_quantile))
```

where read_quantile = 0.9 is used to discard read depths that are too high to be reliable. Genotype imputation was then performed by function estGeno in GBScleanR

```
gdate <- estGeno(object = gdata,
    recomb_rate = 0.04,
    iter = 4,
    error_rate = 0.0025,
    het_parent = FALSE,
    call_threshold = 0.9,
    optim = TRUE)
```

where recomb_rate is in unit of Morgan/Mbp so that the same value of 4 cM/Mbp is specified, iter = 4 specifies the number of iterative parameter updates (2 by default), and the remaining options are set by default.

Apple CP

The apple CP data was analyzed using the same steps for the rice F2 except the following different option values.

Set isfounderinbred = false in functions magicfilter, magicmask, magicimpute, and magicreconstruct. Set designcode to "Golden/Scarlet=>0" for F1 cross without selfing. Set delsamples = nothing (no sample deletion) and maxmiss = 0.9 in function vcffilter. Set missfilter = (f,o) -> o <= 0.8 in magicfilter for filtering for markers with genotype missing fraction in offspring ≤ 0.8, since the apple CP has much smaller population size. Set recomrate = 2.5 and isrepeatimpute = true in magicimpute; the default of not repeating founder analysis was used in the rice F2.

Sorghum MAGIC

A genotypic data file in the VCF format was prepared based on the online supplemental data. The function vcffilter was not performed and magicfilter was performed with options minmaf = 0.05 and missfilter = (f,o) -> f < 0.8 && o < 0.8. Since the breeding pedigree is not known, the population design was set as follow

```
sorghum_design="nfounder=29||ibd=1.0||mapexpansion=11"
```

where the 29 founders must come before offspring in the VCF file, `ibd=1.0` denotes all offspring are assumed to be completely inbred, and `mapexpansion=11` denotes the prior expected breakpoint density is 11 per Morgan per offspring. The function `magicmask_impute` in the RABBIT, a combination of `magicmask` and `magicimpute`, was performed

```
magicmask_impute(sorghum_genofile,sorghum_design;
     isfounderinbred = true,
     model = "depmodel",
     isphysmap = true,
     recomrate = 2.3,
     byfounder = 5,
     isrepeatimpute = true,
     tukeyfence = 2,
     iscorrectfounder = true,
     isspacemarker = true,
     skeletonsize = nothing,
)
```

where `model` is set to "depmodel" since the sorghum population is a homozygous population; general default "jointmodel" was used for the rice F2 and the apple CP. The opitons `isphysmap` and `recomrate` were used to obtained initial genetic map before inferring inter-marker distances. The options `likeparam`, `threshlikeparam`, and `softhreshlikeparam` are set to the default values, where only `offspringerror` is relevant for "depmodel". The option `byfounder` specifies the upper bound block size 5, since there are 10 founders being completely missing. The option `skeletonsize = nothing` indicates the number of skeleton markers is internally determined by the number of different genetic positions in each chromosome. A new `sorghum_genofile2` was output.

We then run function `magicreconstruct` in the RABBIT

```
magicreconstruct(sorghum_genofile2,sorghum_design;
 isfounderinbred = true,
 hmmalg = "forwardbackward",
)
```

where `hmmalg` denotes the forward backward algorithm for haplotype reconstruction. The resulting posterior ancestral haplotype probabilities were saved in a text file `ancestryfile`.

Lastly, we performed QTL mapping using the `statgenMPP` package.

```
sorghum_mqm <- selQTLMPP(sorghum_mpp,
    trait = height,  threshold = 5, maxCofactors = NULL,
    computeKin = true, parallel = TRUE)
```

where object `sorghum_mpp` was produced from `ancestryfile`, `trait` denotes the mean height data between 2013 and 2014, `maxCofactors` denotes the maximum number of co-factors to include in the model, `threshold` denotes the threshold of $-log10$ p-value for detecting a QTL, and `computeKin` denotes whether to calculate chromosome specific kinship matrices and include them in the model.

Guidance on MagicImpute options

The main options of MagicImpute are listed in Table S2, together with default values and descriptions. Here we provide a detailed guidance on calibrating these parameters.

isfounderinbred The default `isfounderinbred`=true refers to completely inbred founders. Set `isfounderinbred`=false if there are many residual heterozygous genotypes in inbred founders.

likeparam By default, infer `offspringerror` for called genotypes, and infer `offspringerror`, `allelicbias`, and `allelicoverdispersion` for GBS data. To reduce computation time, set `likeparam=LikeParam(allelicbias=0.5,allelicoverdisersion=0)` for input GBS data without allelic bias and overdispersion. Infer `peroffspringerror`, i.e. set `likeparam=LikeParam(peroffspringerror=nothing)`, if input genotypic data were obtained from different genotyping platforms. Infer `baseerror` if sequence base error is expected to be much larger than 0.001.

threshlikeparam We have set quite high hard thresholds by default, although threshold 1.0 for allelicoverdispersion may be increased if there are too many deletions due to large overdispersion.

softthreshlikeparam and tukeyfence Increase `softthreshlikeparam` or `tukeyfence` if there are too many deleted markers. If stringent marker filtering is desired, decrease `softthreshlikeparam` or `tukeyfence` instead of `threshlikeparam`.

isinfererror To reduce computation time, set `isinfererror` = false and `offspringerror` to a small fixed value, e.g. `likeparam=LikeParam(offspringerror=0.005)` for homozygous mapping populations with a high quality of called genotypes.

isdelmono Keep default true to reduce computational time.

isbinning and bincm By default, `isbinning` is internally set to true if average bin size > 1.5, which usually holds for high density markers. Here bin size is defined as the number of markers in a bin. For medium marker density with small bin size, increasing `bincm`, e.g. from default 0.001 to 0.005 cM, would increase the average bin size.

isrepeatimpute and nrepeatmin and nrepeatmax By default, `isrepeatimpute` is internally set to true if there is a founder with missing fraction > 0.6, where missing fraction is calculated in such a way that heterozygous genotypes are regarded as missing because of unknown phases. The maximum number of parallel workers is given by the number of chromosomes times `nrepeatmin` if `nrepeatmin` equals `nrepeatmax`, and otherwise it is given by the number of chromosomes.

iscorrectfounder It is recommended to keep default true unless there are too many suspicious false corrections.

isdelvuong and desiglevel Unimportant when inferring error parameters. If `isinfererror` = false, set `isdelvuong` = true, and set `desiglevel` to 0.001 to reduce marker deletion and to 0.05 to increase marker deletion.

isspacemarker and trimcm To reduce computational time, set `isspacemarker` = false. Increase `trimcm` from 20 to 40 cM If many markers are trimmed.

threshar2 Keep default 0.3 to maintain many variants. Increase to 0.7-0.9 for stringent quality control.

blockdelmarker A quick way to disable all kinds of marker deletion, mainly for studying the effects of marker deletion.

Supplementary Tables

Table S1: List of symbols and their brief descriptions

Symbol	Description
$P(x), P(y\|x)$	Probability of x, conditional probability of y given x
Beta(α, β)	Beta distribution with two shape parameters α and β
t	Subscript for a marker
i	Subscript for an offspring
f	Superscript for a founder
F	Superscript for a set of all founders
Ω	Superscript for a subset of founders
Ω_b	Founder block b in a partition of founders
y_{ti}	Observed genotypic data of offspring i at marker t
z_{ti}	Hidden genotype of offspring i at marker t,
x_{ti}	Hidden origin-genotype of offspring i at marker t,
ϵ_{ti}	Allelic error probability of offspring i at marker t,
ϵ^F	Allelic error probability in founders, fixed $\epsilon_F = 0.005$
ϵ_t^F	Allelic error probability in founders at marker t in simulations
y_t^f	Observed genotypic data of founder f at marker t, $\boldsymbol{y}_t^\Omega = \left\{y_t^f\right\}_{f\in\Omega}$
h_t^f	Hidden genotype of founder f at marker t, $\boldsymbol{h}_t^\Omega = \left\{h_t^f\right\}_{f\in\Omega}$, $\boldsymbol{h}^\Omega = \left\{h_t^\Omega\right\}_t$
$\hat{\boldsymbol{h}}_t^F$	Estimated phased founder genotypes at marker t, $\hat{\boldsymbol{h}}^F = \left\{h_t^F\right\}_t$
r_0	Number of sequence reads for allele 0
r_1	Number of sequence reads for allele 1
ξ_i	Allelic error probability of offspring i
ϵ_t	Allelic error probability at marker t
η_t	Sequence base error probability at marker t
μ_t	Mean allelic balance at marker t, $\mu_t - 1/2$ measures allelic bias
ϕ_t	Overdispersion of allelic depths at at marker t
l_{ti}	Likelihood of genotype y_{ti}
N_b	Upper bound of founder block sizes in a founder partition

Table S2: List of main options of MagicImpute

Option	Description
isfounderinbred	Default: true. If true, all founders are completely inbred.
isphysmap	Default: false. If true, transform physical map into genetic map using recomrate.
recomrate	Default: 1.0. Constant recombination rate in cM/Mbp.
likeparam	Default: LikeParam(0.005,nothing,0.0,0.001,nothing,nothing,0.0)*. Infer parameters with values being nothing, and otherwise they are fixed.
threshlikeparam	Default: ThreshLikeParam(0.25,0.25,0.25,0.05,0.9,1.0,0.1)*. Delete marker with estimated values greater than the thresholds.
softthreshlikeparam	Default: SoftThreshLikeParam(0.025,0.025,0.025,0.01,0.67,0.5,0.01)*. Delete marker with estimated values greater than the thresholds only if they are also outliers.
tukeyfence	Default: 2. Outlier marker detection is based on the Tukey's fence.
isinfererror	Default: true. If true, infer parameters specified by likeparam.
isdelmono	Default true. If true, delete monomorphic or uninformative markers.
isbinning	Default: true if average bin size > 1.5. If true, bin markers prior to imputation.
bincm	Default: 0.001. Bin markers with distance < bincm in cM if isbinning.
isrepeatimpute	Default: true if there is a founder with missing fraction > 0.6. If true, repeat founder imputation from npreatmin to nrepatmax times.
nrepeatmin	Default: 3. Minimum number of repeating imputation.
nrepeatmax	Default: 6. Maximum number of repeating imputation.
iscorrectfounder	Default: true. If true, perform founder error correction.
isdelvuong	Default: true. If true, perform marker deletion based on Vuong's closeness test.
delsiglevel	Default: 0.01. Significance level for marker deletion if isdelvuong.
isspacemarker	Default: isphysmap. If true, refine inter-marker distances.
trimcm	Default: 20. Delete a segment at chromosome ends with distance gap > trimcm in cM. The fraction of markers in the segment must $< 2.5\%$.
threshar2	Default 0.3. Delete markers with AR2 being NaN or < threshar2. If threshar2 equals 0, no deletion.
blockdelmarker	Default: false. If true, reset relevant options to block marker deletion: isdelmono = false, isdelvuong = false, threshar2 = 0, trimcm = Inf, tukeyfence = Inf, threshlikeparam = ThreshLikeParam(Inf,Inf,Inf,Inf,Inf,Inf,Inf).

* LikeParam, ThreshLikeParam, SoftThreshLikeParam are keyword structs with keywords being the likelihood parameters: foundererror, offspringerror, peroffspringerror, baseerror, allelicbias, allelicoverdispersion, and allelicdropout. The first six parameters corresponding to ϵ_t^F,ϵ_t, ξ_i, η_t, μ_t, and ϕ_t, respectively. By default, allelicdropout is set to 0.0 such that there is no allelic dropout, and this was not investigated in current study.

Supplementary figures

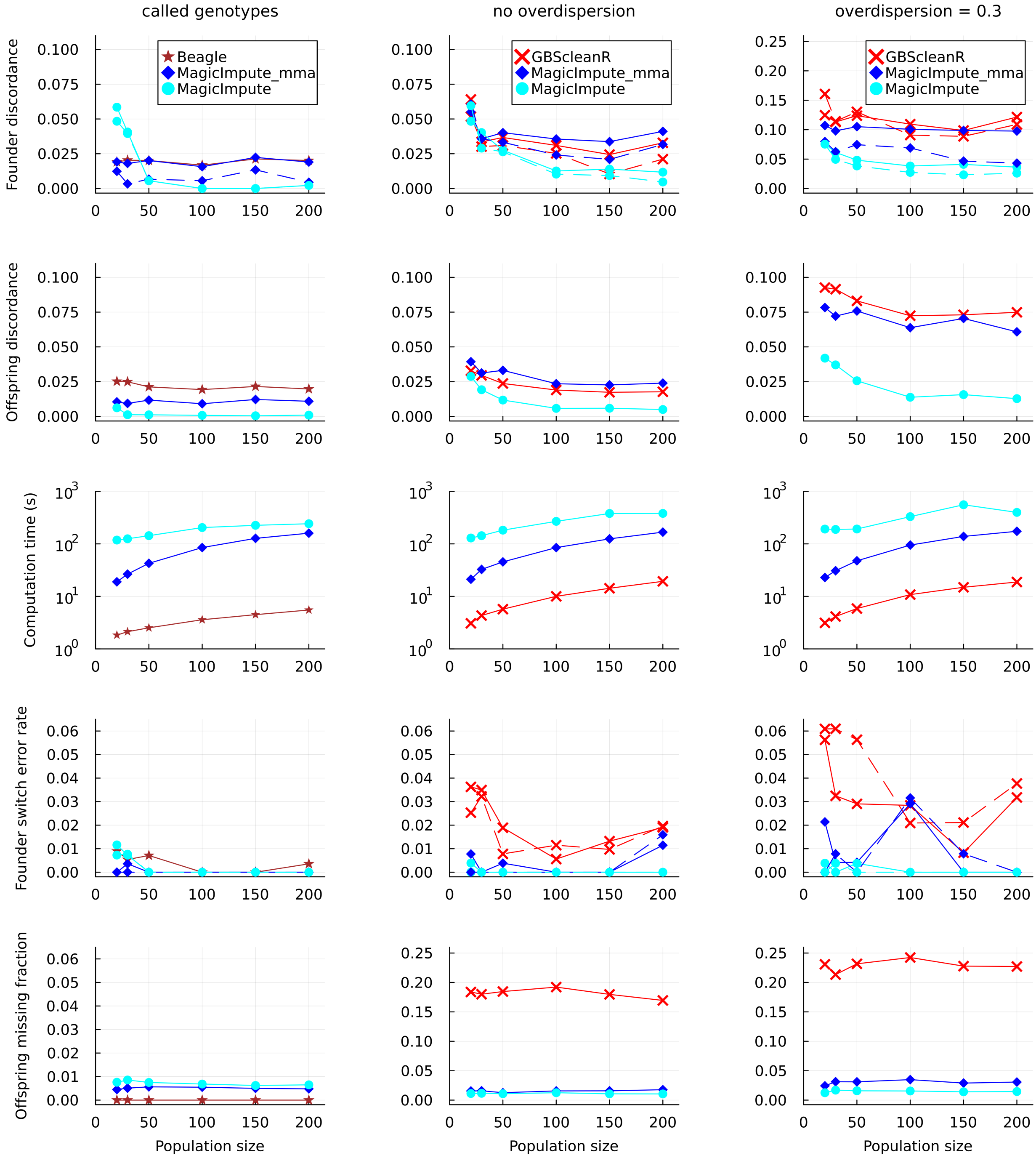

Figure S1: Comparisons of MagicImpute with MagicImpute_mma, Beagle, and GBScleanR in the simulated CP populations with two founders (see Figure 1A). Beagle worked only for called genotypes in the left panel and GBScleanR worked only for GBS data in the middle and right panels. In the first and fourth rows, the dashed lines refer to the founders with all genotypes missing, and the solid lines refer to the other founders.

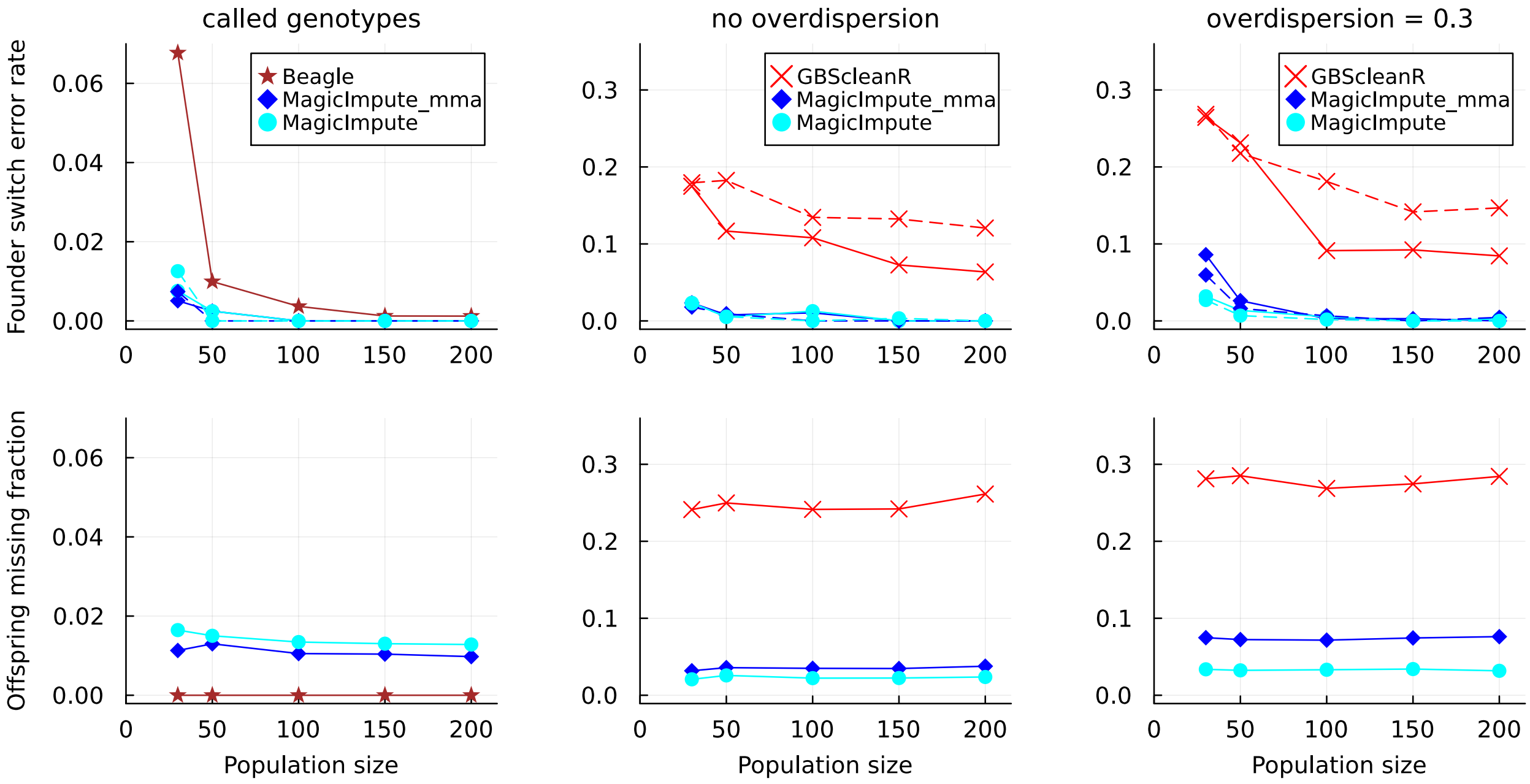

Figure S2: Similar to Figure 2 but from the perspective of parental phasing and offspring imputation.

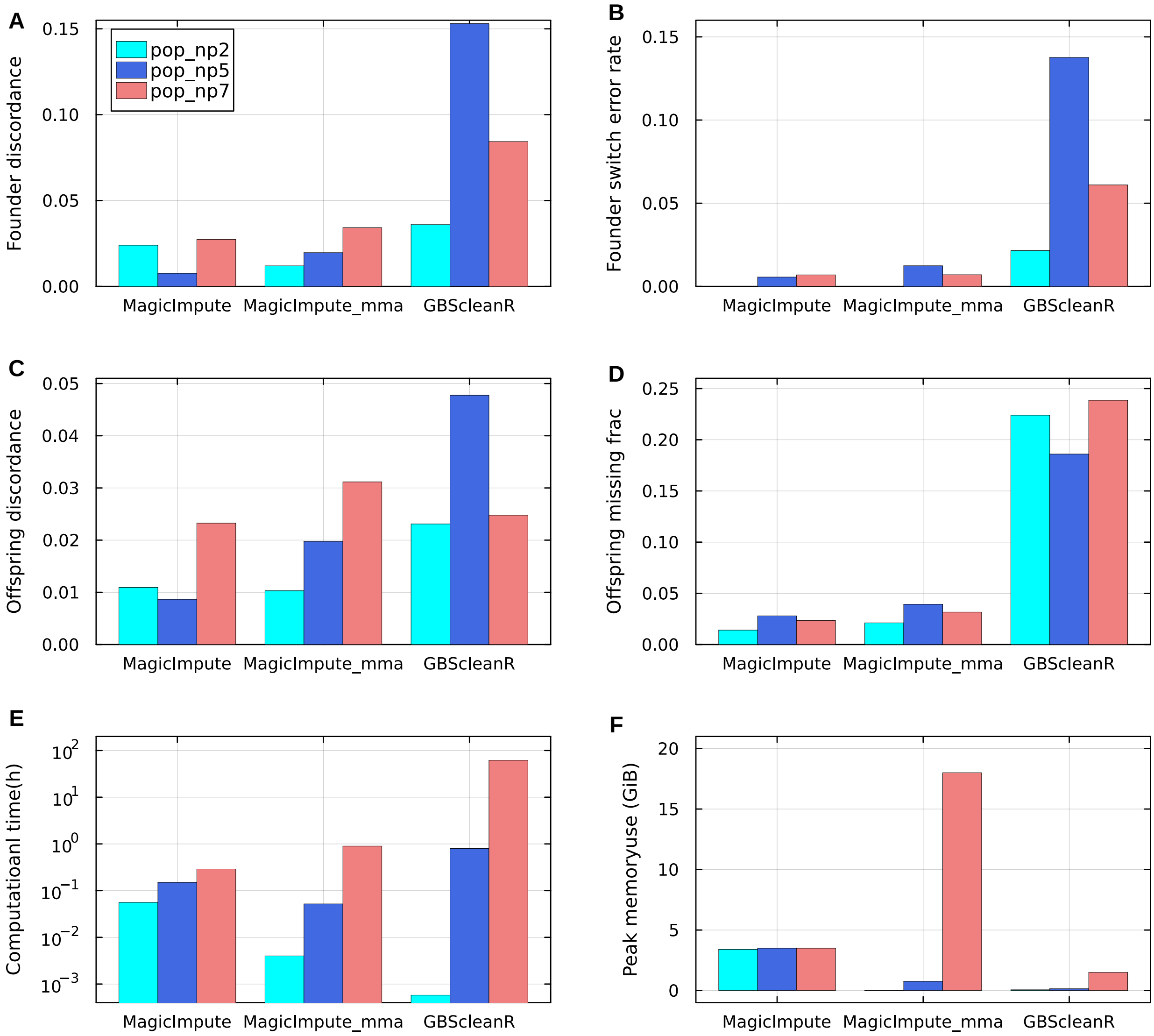

Figure S3: Comparisons of MagicImpute with MagicImpute_mma, Beagle, and GBScleanR in the three simulated populations of size 100 (Figure 1A-C). In the legend, pop_np2, pop_np5, and pop_np7 denote the three populations with the numbers 2, 5, and 7 of founders, respectively. The genotypic data were called genotypes without overdispersion. Only one linkage group was analyzed such that MagicImpute was performed without parallel processing, whereas MagicImpute_mma and GBScleanR were performed with five parallel threads. In panel D, the peak memory usages of MagicImpute were measured at the operating system level, which included items like shared memory that were excluded for MagicImpute_mma and GBScleanR.

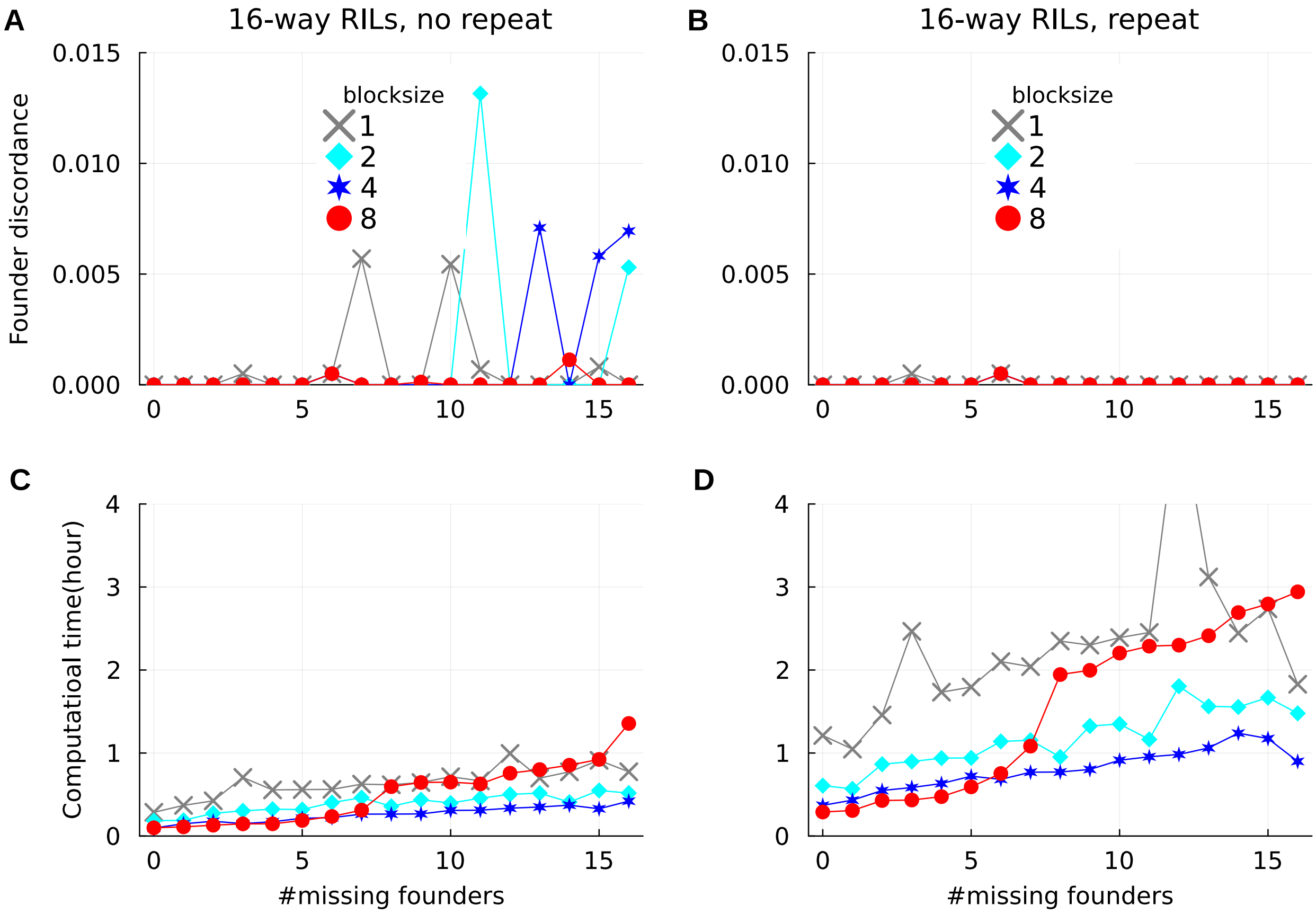

Figure S4: Founder imputation in the simulated 16-way RILs with population size 500. The x-axis denotes the number of founders with all genotypes missing.

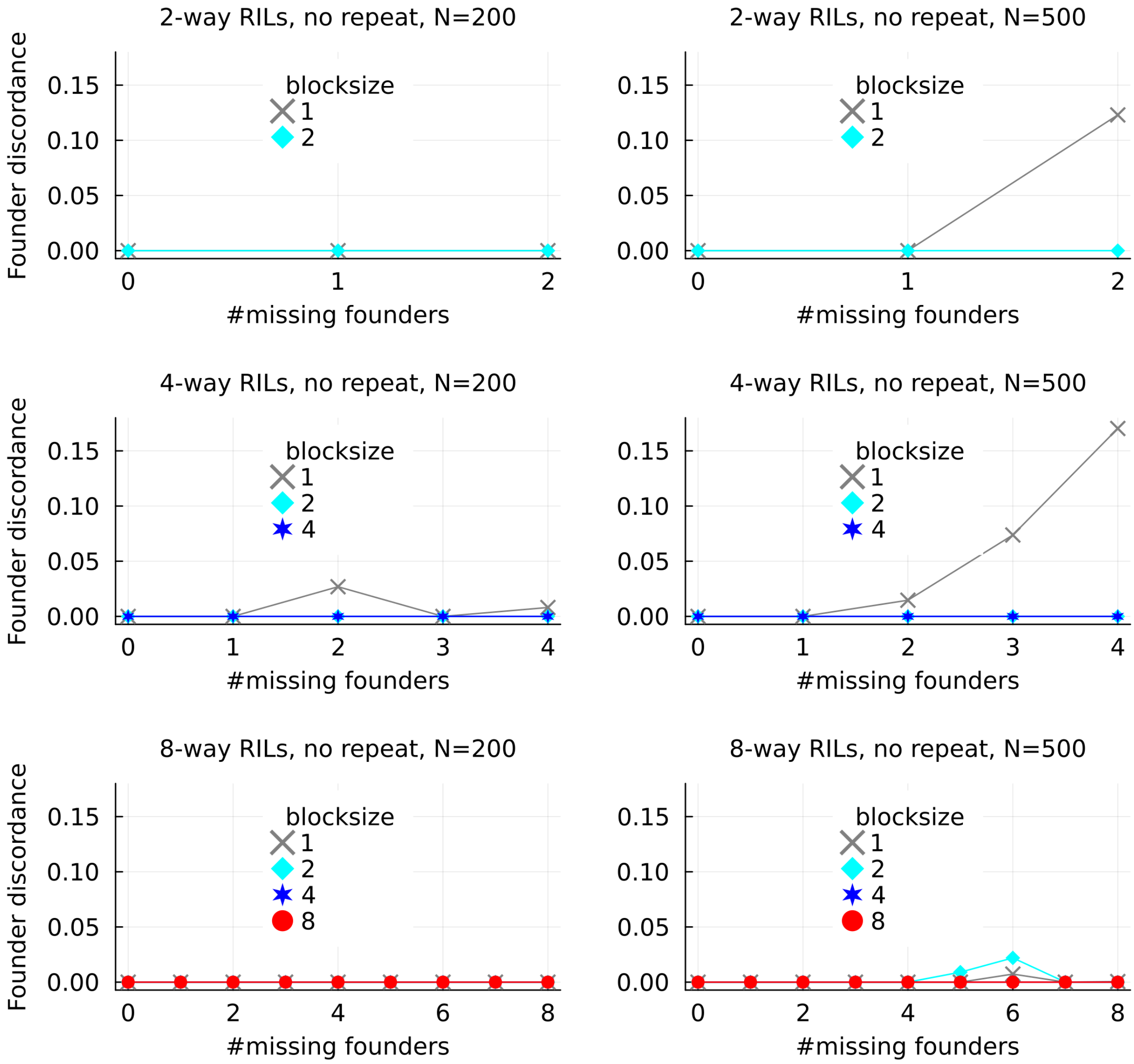

Figure S5: Founder imputation in the simulated 2, 4, and 8-way RILs without repeating imputation. The x-axis denotes the number of founders with all genotypes missing.

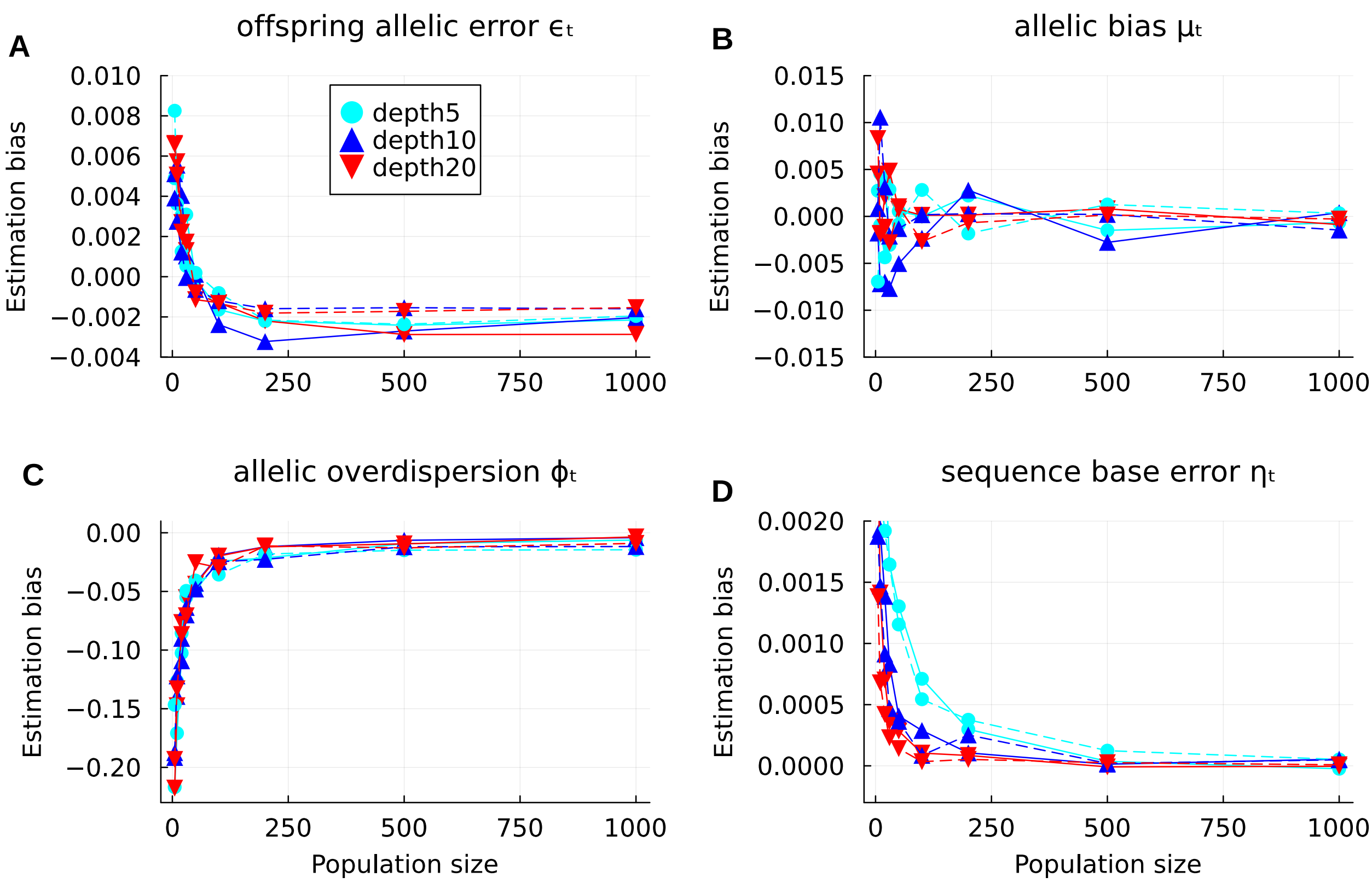

Figure S6: Estimation bias for the error rates in the simulated F2 (dashed lines) and CP (solid lines). For the sake of visualization, results for depth 40 are not shown.

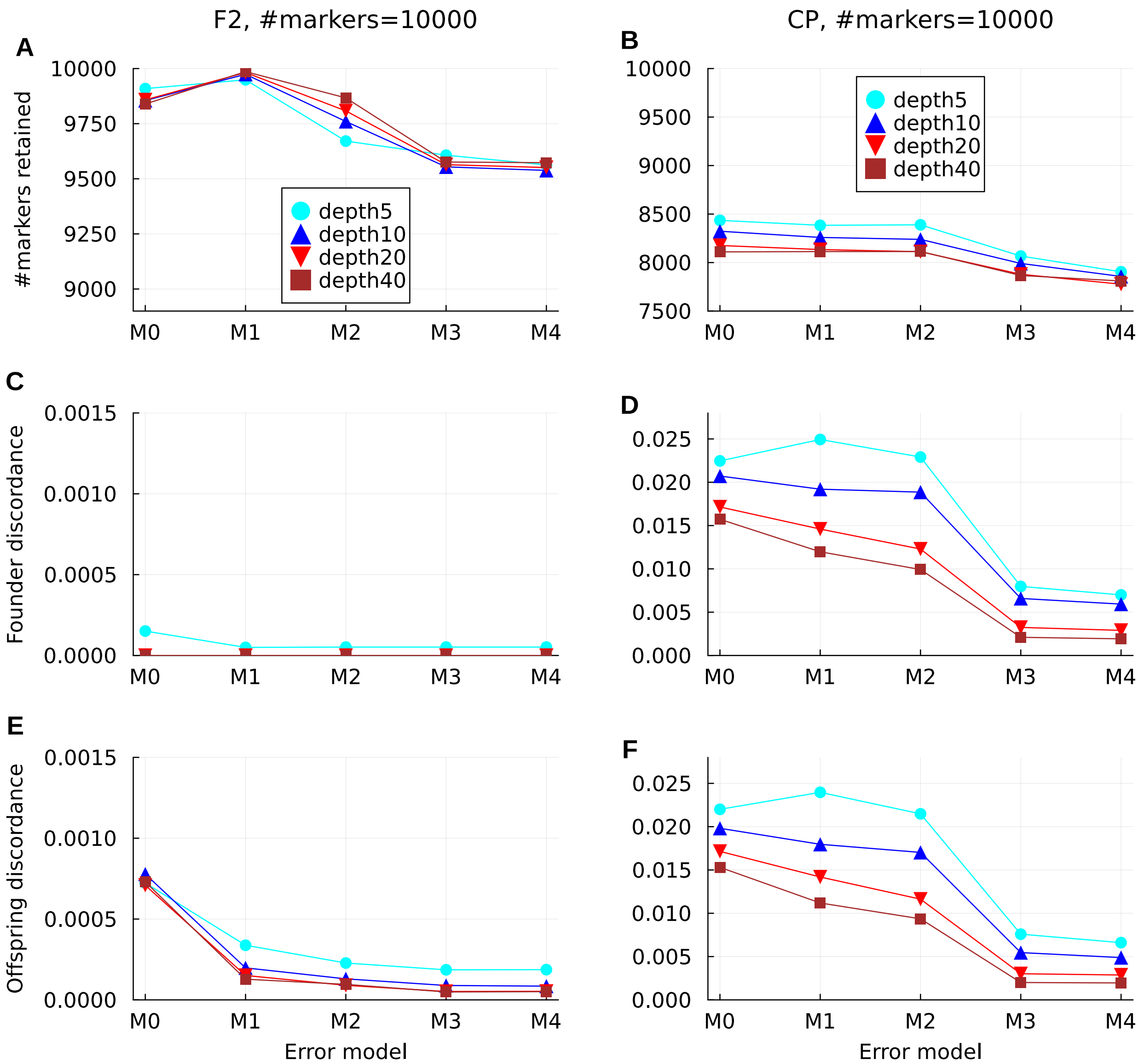

Figure S7: Genotype estimation in simulated F2 and CP without the founder error correction. The results were obtained with the default soft- and hard-threshold-based marker deletion.

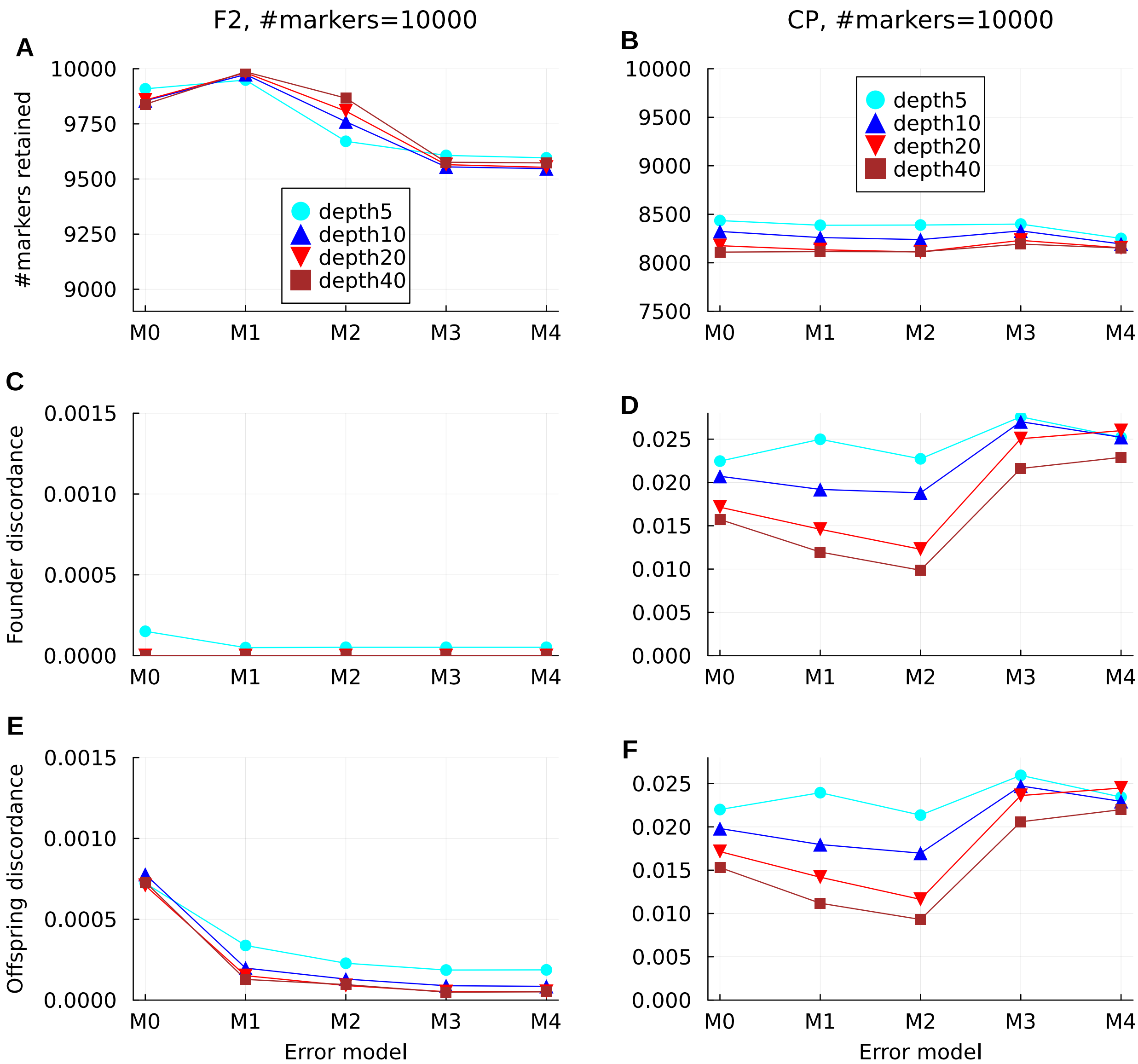

Figure S8: Genotype estimation in simulated F2 and CP with no founder error correction and no soft-threshold-based marker deletion. The results were obtained with the default hard-threshold-based deletion.

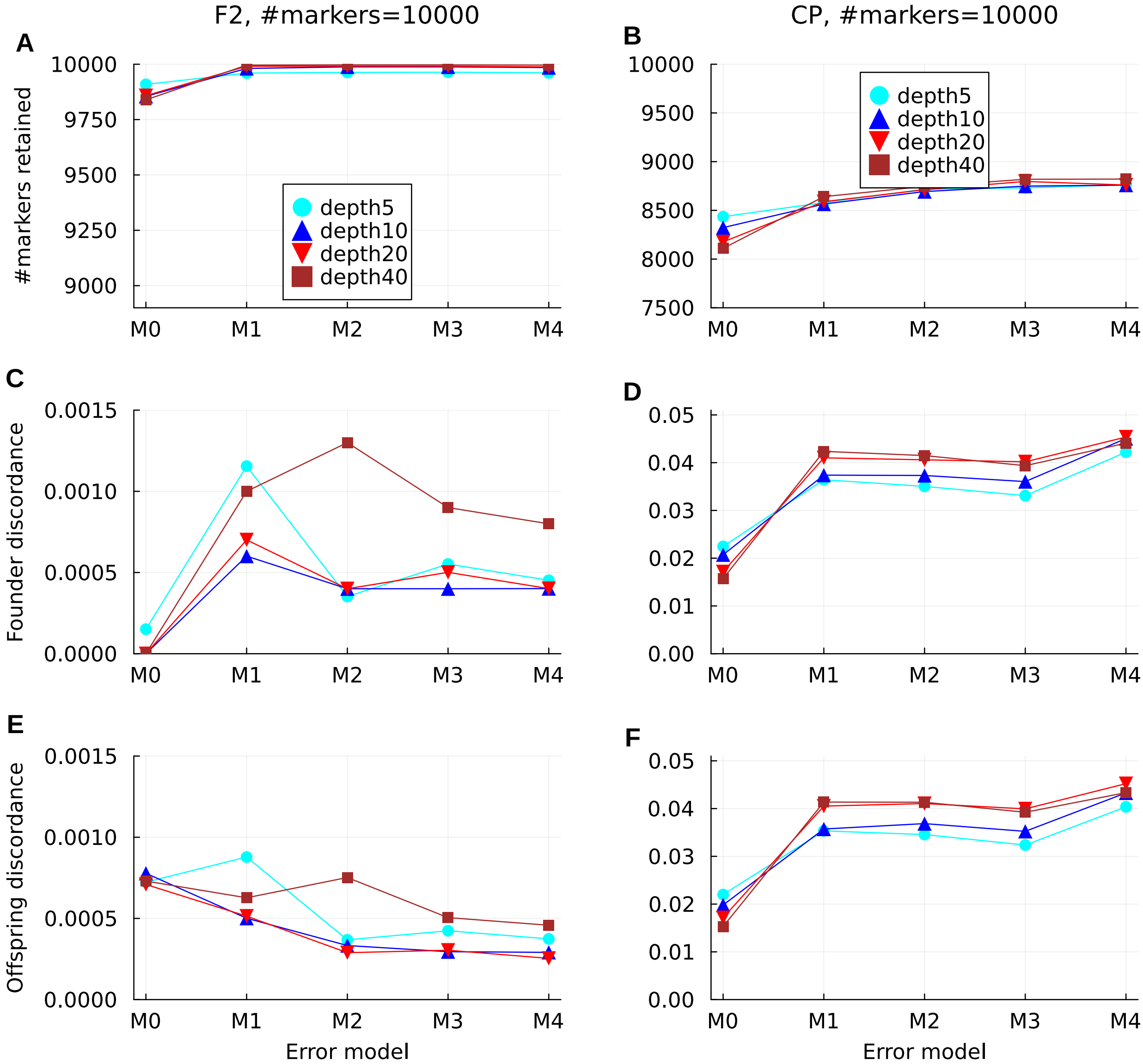

Figure S9: Genotype estimation in simulated F2 and CP with no founder error correction and no soft- or hard-threshold-based deletion.

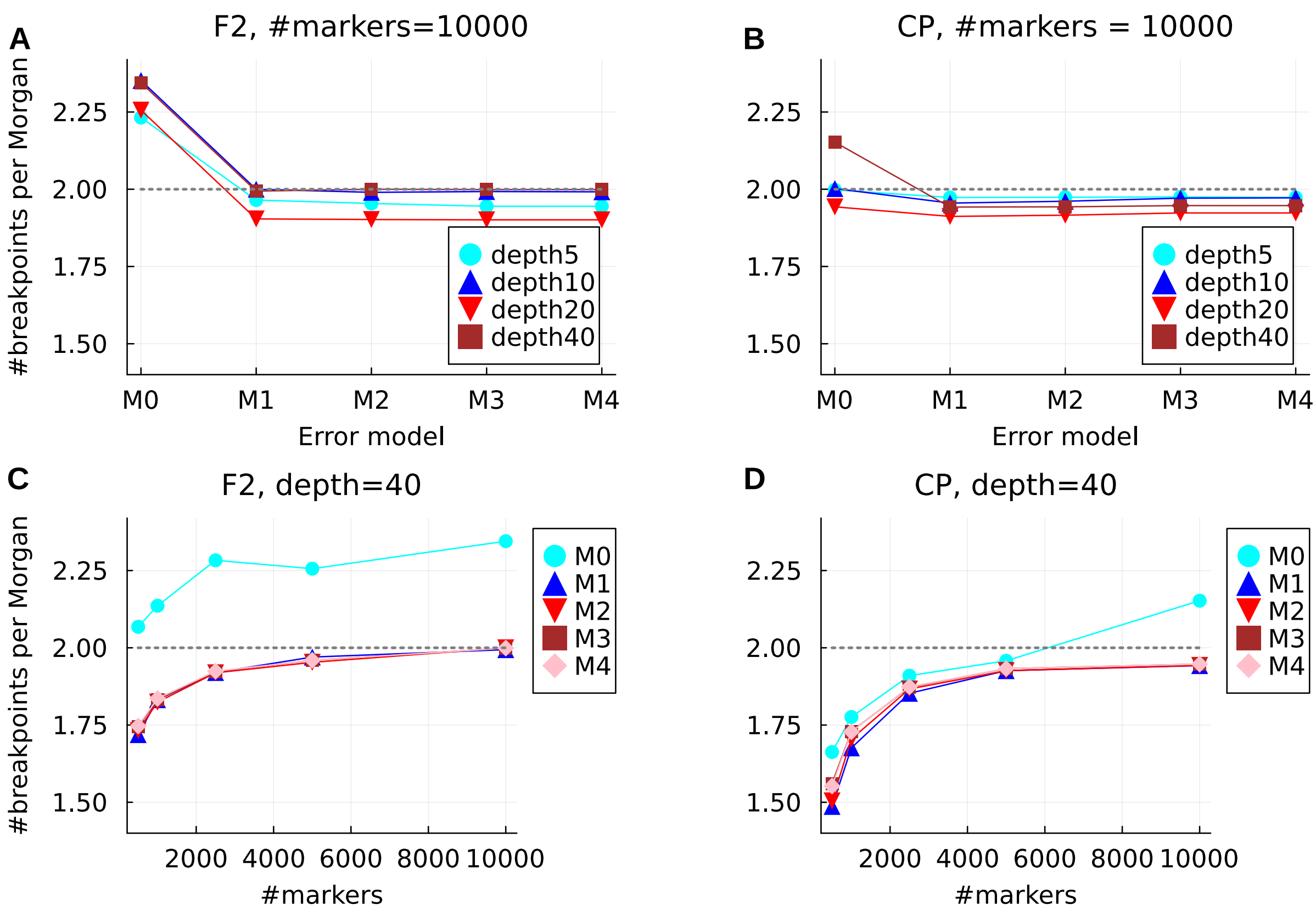

Figure S10: Estimated breakpoint density from the imputed genotypic data in the simulated F2 and CP. The error models are defined in Table 1. The horizontal dotted lines denote the prior number of breakpoints per Morgan per offspring.

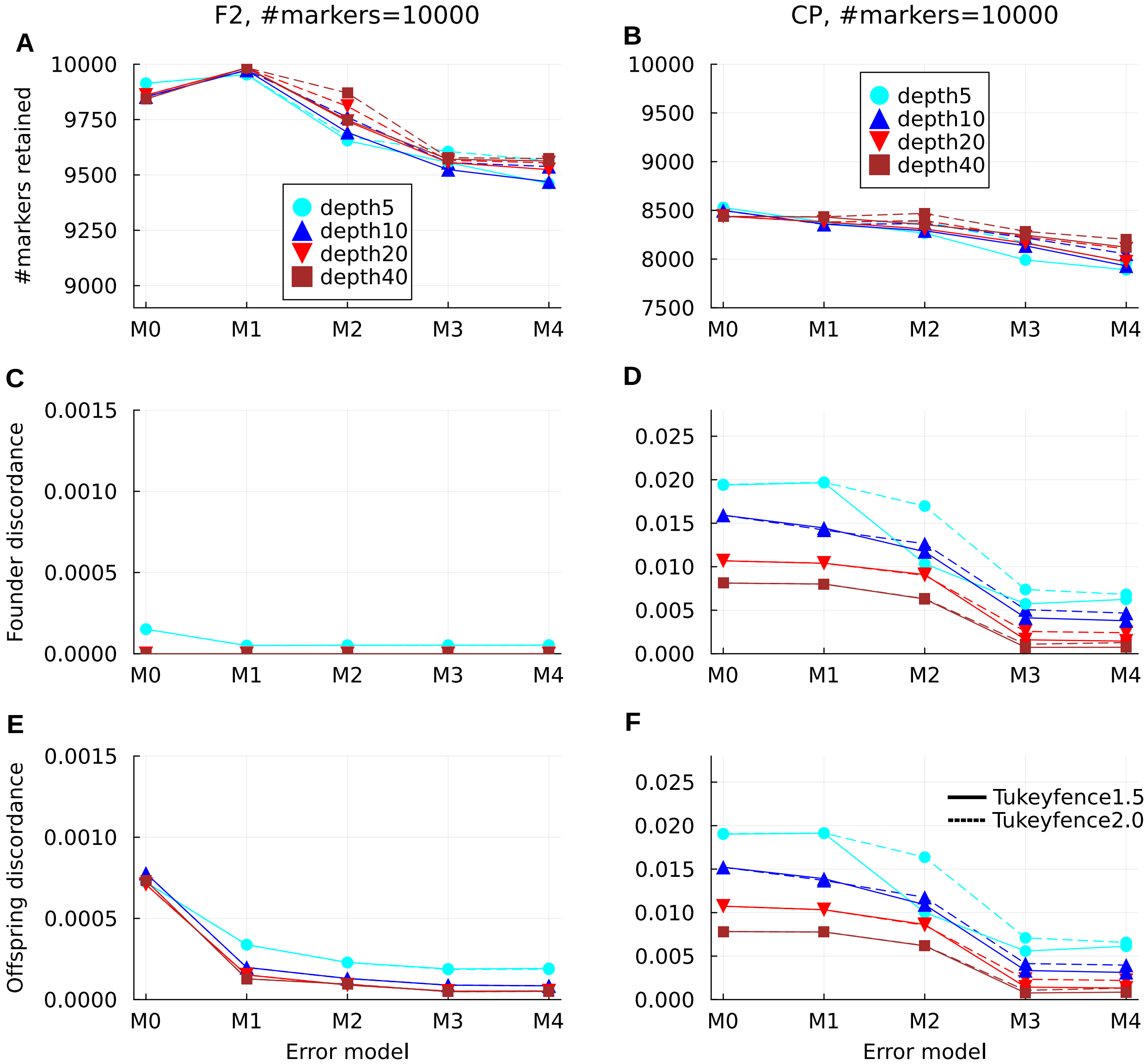

Figure S11: Sensitivity analysis in simulated F2 and CP with Tukey's fence 1.5 (solid lines). The dashed lines denote the same as Figure 5 with default Tukey's fence 2.

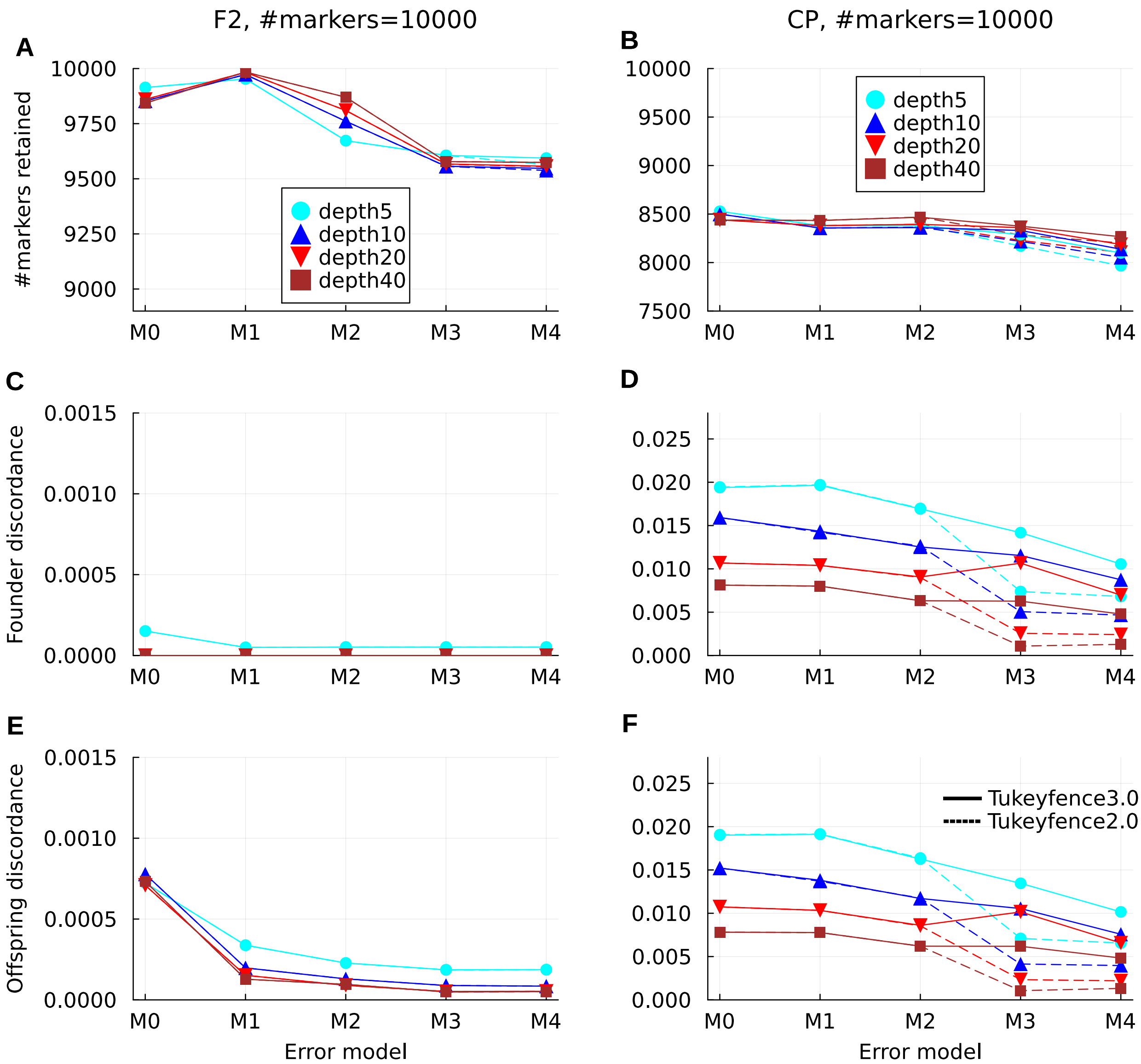

Figure S12: Sensitivity analysis in simulated F2 and CP with Tukey's fence 3 (solid lines). The dashed lines denote the same as Figure 5 with default Tukey's fence 2.

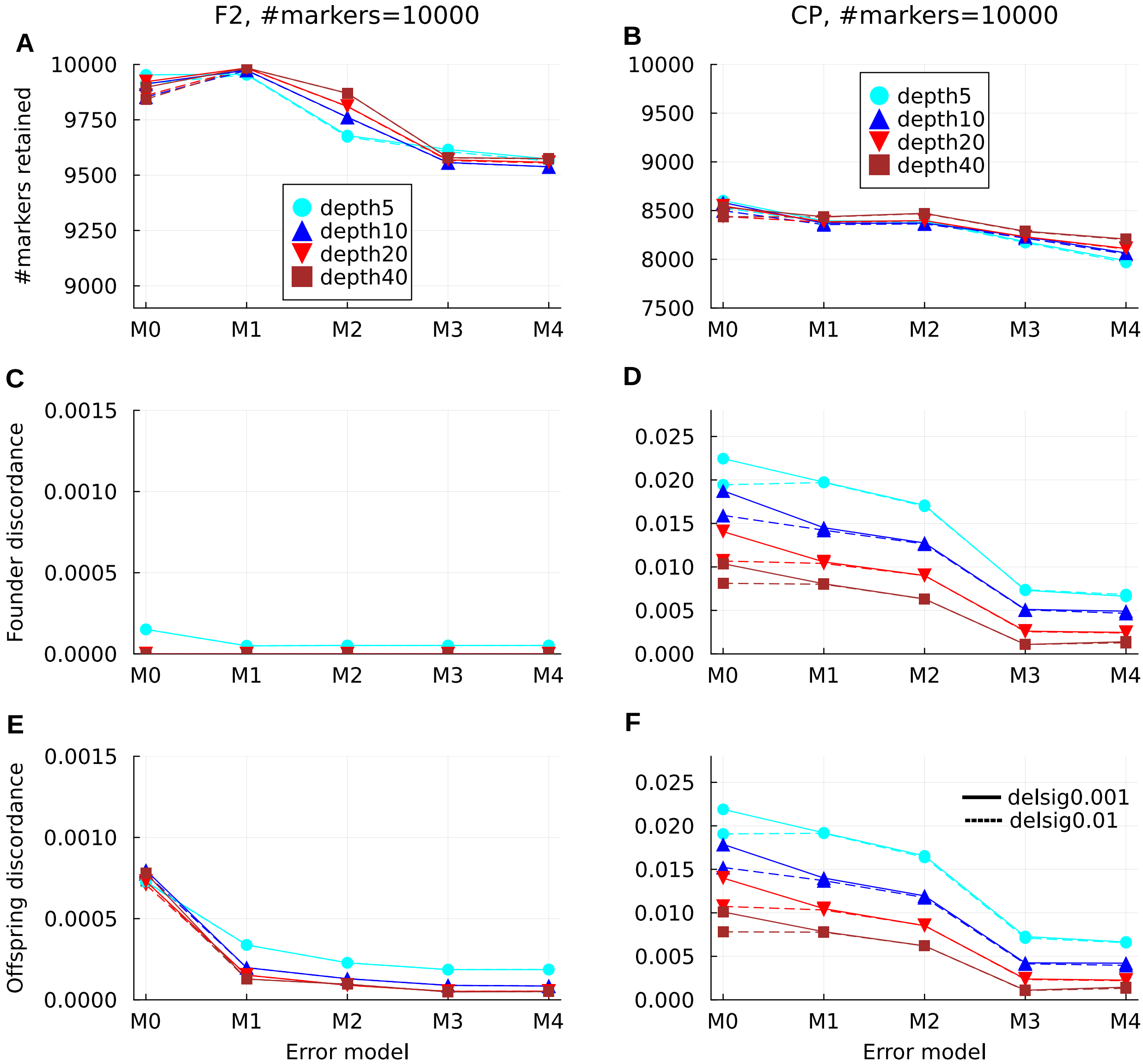

Figure S13: Sensitivity analysis in simulated F2 and CP with significance level 0.001 for the Vuong's test based marker deletion (solid lines). The dashed lines denote the same as in Figure 5 with default significance level 0.01.

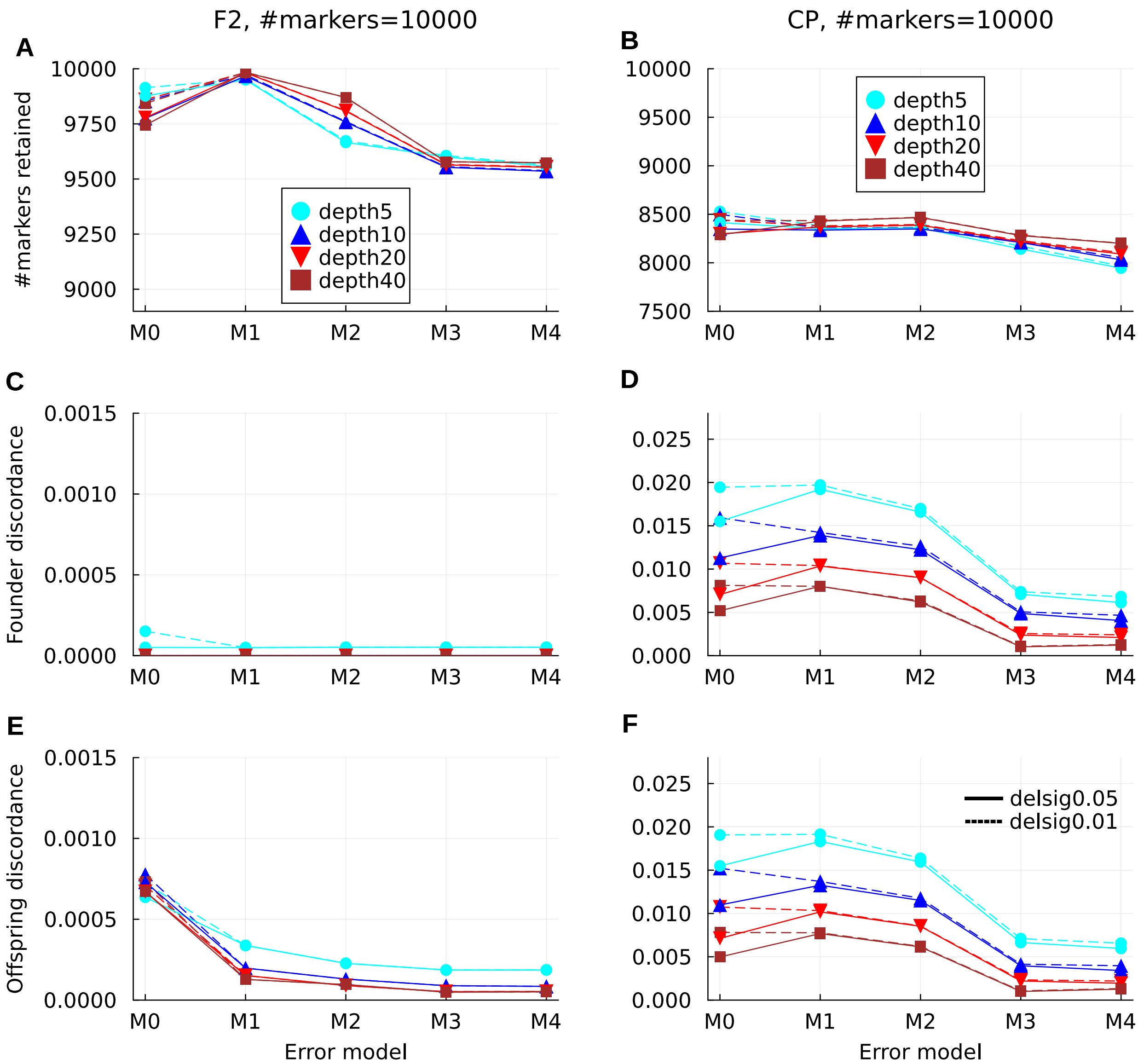

Figure S14: Sensitivity analysis in simulated F2 and CP with significance level 0.05 for the Vuong's test based marker deletion (solid lines). The dashed lines denote the same as in Figure 5 with default significance level 0.01.

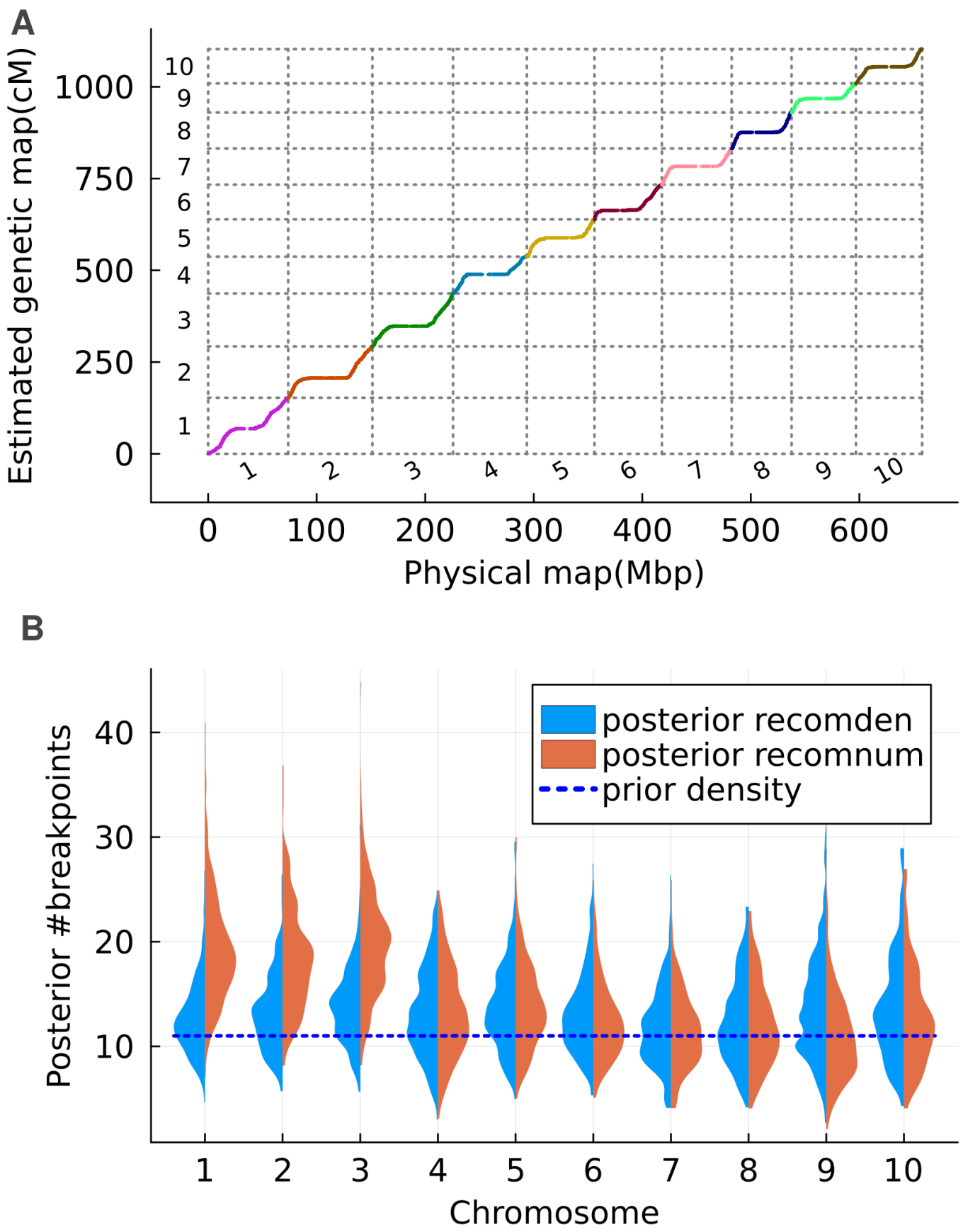

Figure S15: Estimated genetic map and number of breakpoints per chromosome in the real sorghum MAGIC. In panel B, "recomnum" denotes the number of breakpoints per offspring, and "recomden" denotes the number of breakpoints per Morgan per offspring.

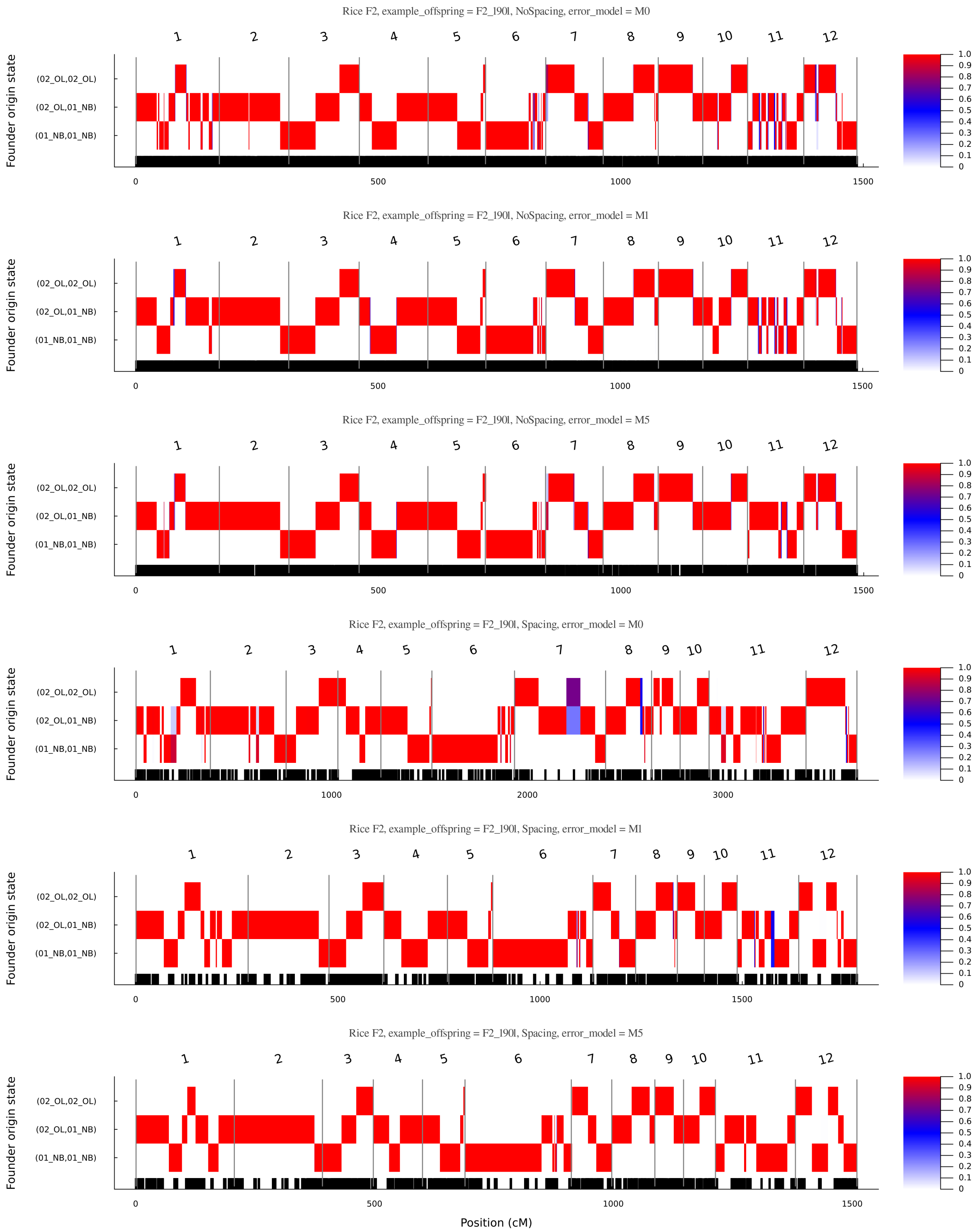

Figure S16: Posterior ancestral probabilities for an example offspring in the real rice F2. The first three panels refer to the results without inferring inter-marker distances, and the remaining panels refer to the results with inferring distances.

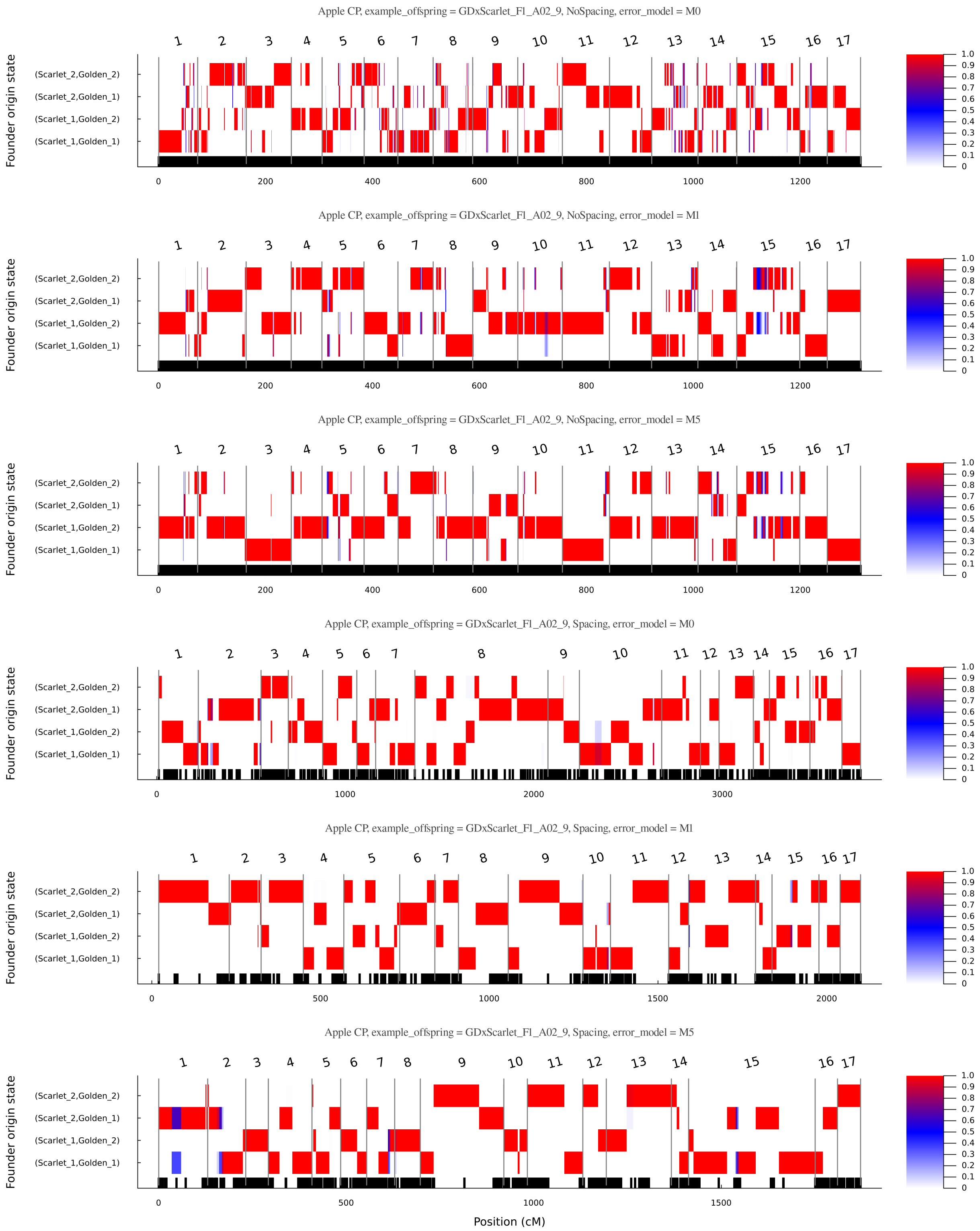

Figure S17: Posterior ancestral probabilities for an example offspring in the real apple CP. Note that the ordering of the two homologs for each parent in each chromosome is non-identifiable and thus the two homologs are arbitrarily labeled.

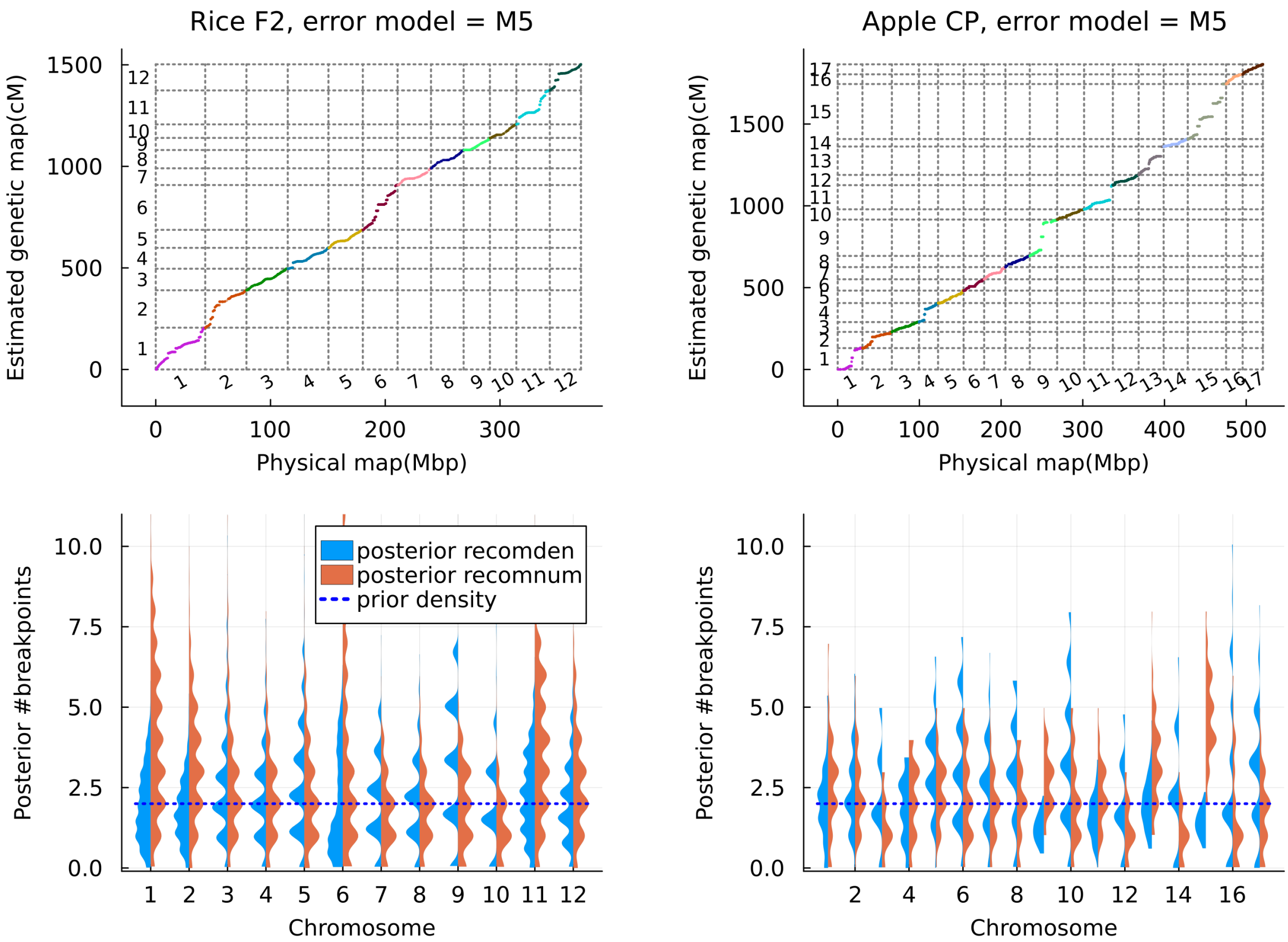

Figure S18: Estimated genetic map and number of breakpoints per chromosome in the real rice F2 and apple CP. In the bottom panels, "recomnum" denotes the number of breakpoints per offspring, and "recomden" denotes the number of breakpoints per Morgan per offspring.

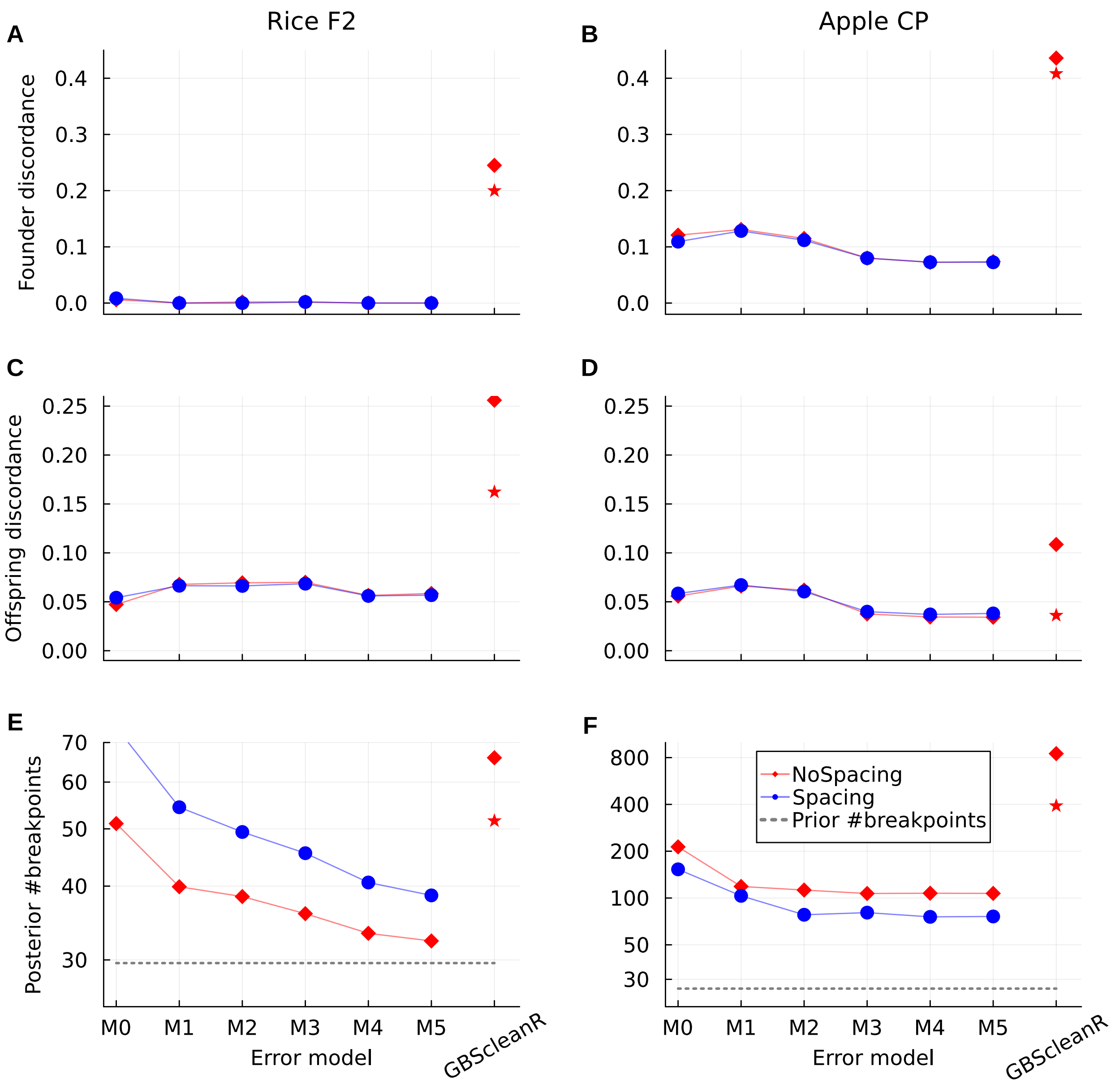

Figure S19: Similar to Figure 7 except that markers were not binned during imputation.

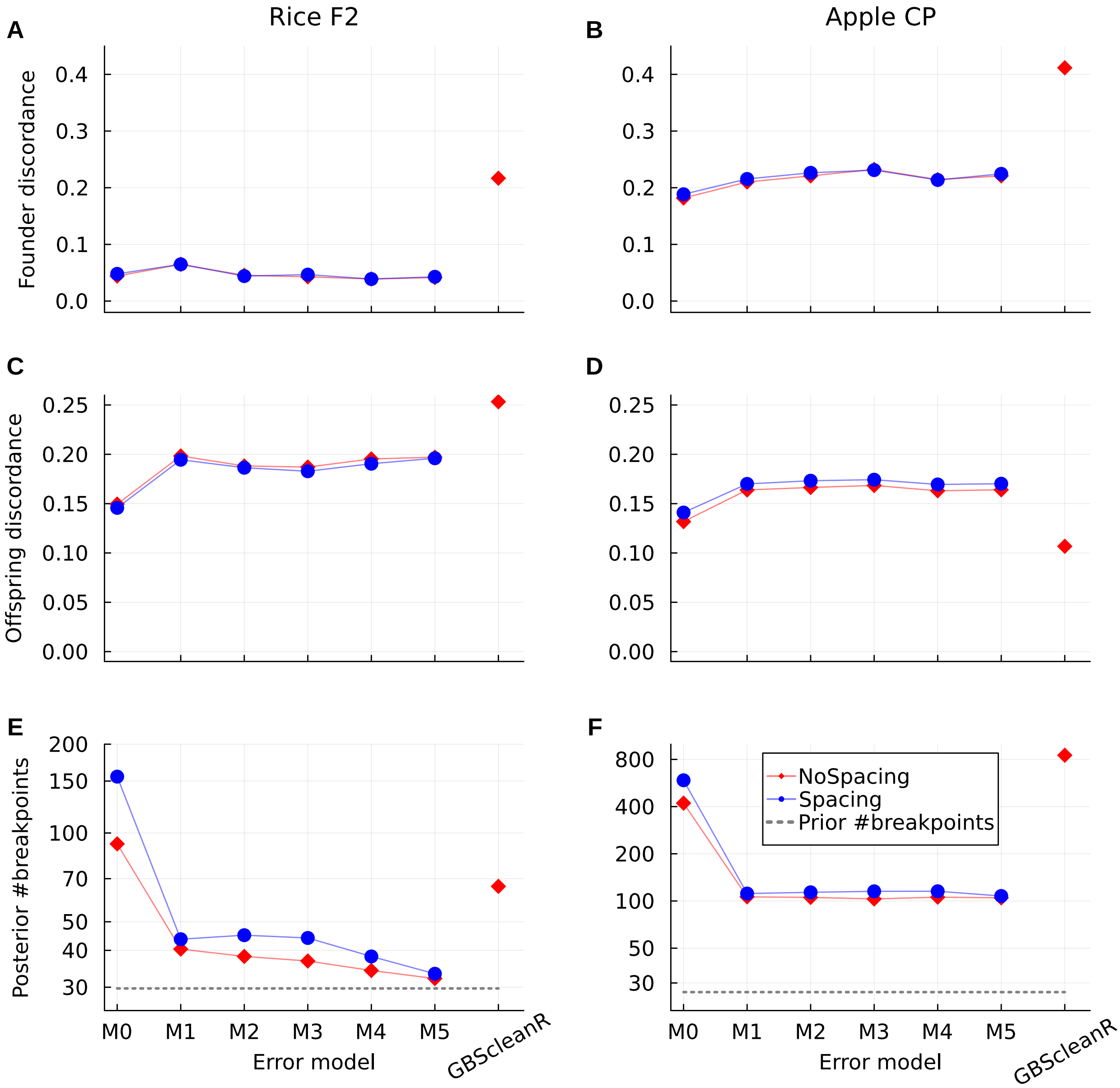

Figure S20: Similar to Figure 7 except that marker deletion was disabled for MagicImpute.

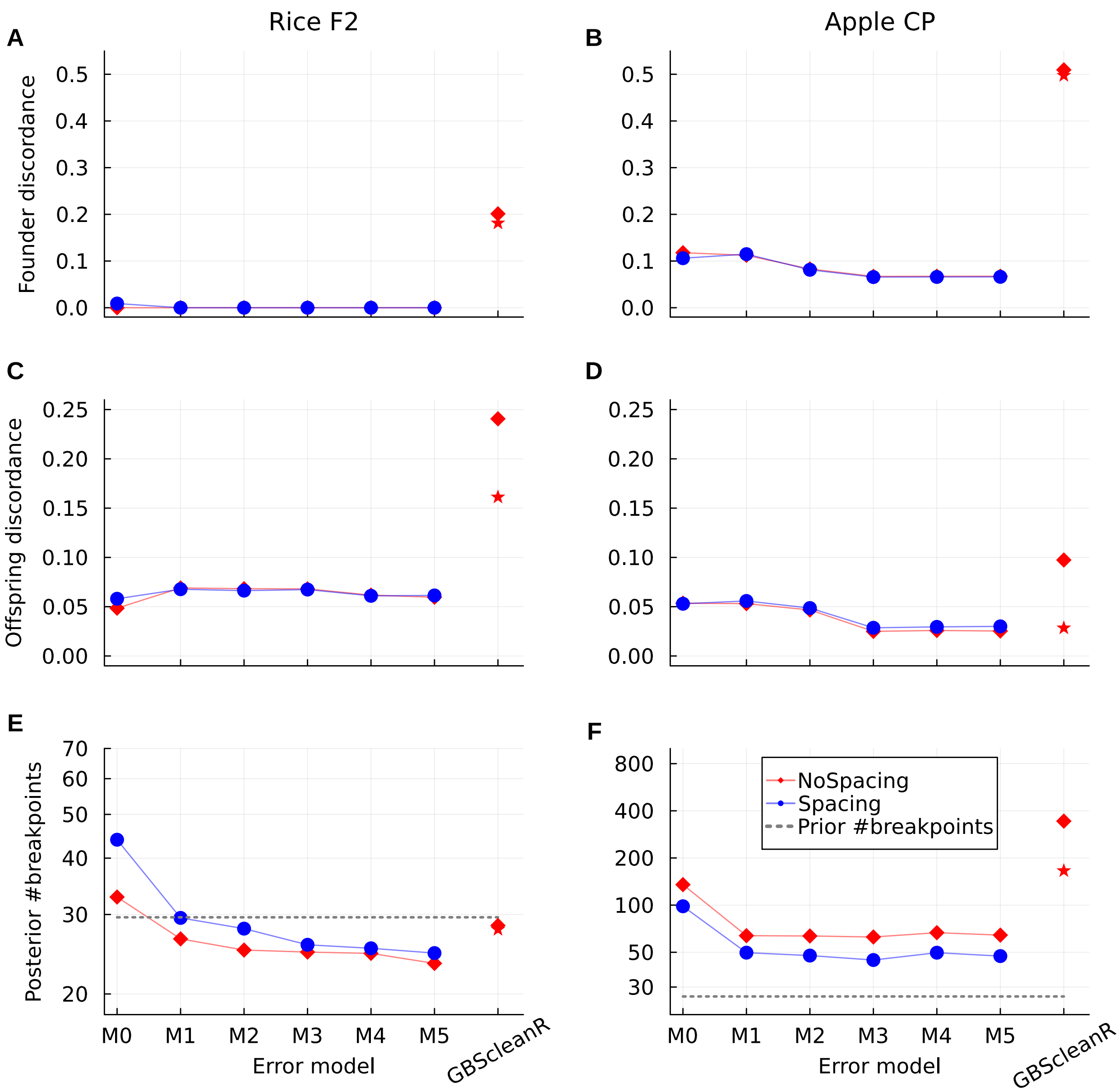

Figure S21: Similar to Figure 7 except that markers were thinned by taking the first marker within each sequence stretch of length 75 bp prior to imputation. The number of markers was reduced by around half in either population.
